# Methods for incorporating test result information within the high-dimensional propensity score framework: application in UK electronic health record data

**DOI:** 10.1186/s12874-026-02926-w

**Published:** 2026-06-30

**Authors:** John Tazare, Jeremy P. Brown, Daniel R. Morales, Liam Smeeth, Stephen J. W. Evans, Ian J. Douglas, Elizabeth J. Williamson

**Affiliations:** 1 Faculty of Epidemiology and Population Health, London School of Hygiene and Tropical Medicine, Keppel St, London, WC1E 7HT UK; 2 Department of Epidemiology, Harvard T.H. Chan School of Public Health, Boston, MA USA; 3 Usher Institute, University of Edinburgh, Edinburgh, UK

**Keywords:** Pharmacoepidemiology, Confounding, Laboratory tests, Propensity scores

## Abstract

**Background:**

Growing availability of laboratory test result information in large healthcare databases presents opportunities to improve confounding control. Whilst information on the ordering of tests and the resulting continuous test values are available, their optimal integration into data-driven confounding control strategies like the high-dimensional propensity score (HDPS) remains unclear.

**Methods:**

We propose methods to incorporate test-related data into HDPS, addressing key concerns around data quality and missing data. We illustrate these approaches using UK Clinical Practice Research Datalink GOLD data comparing COPD-specific mortality in new-users of proton pump inhibitors (PPIs) and H2-receptor antagonists (H2RAs). Hazard ratios (HRs) were estimated using Cox models weighted by inverse HDPS. Integration of 35 blood-test values was achieved via biologically informed cut-offs and using a missing-indicator approach for continuous data. Results were benchmarked against a HDPS model derived from clinical, referral, and prescription data dimensions.

**Results:**

Among 733,885 new PPI and 124,410 H2RA users, the adjusted HR for PPI use and COPD mortality was 1.36 (95% CI: 1.14–1.64) in the primary HDPS analysis. Incorporating test-requested data and continuous blood test values attenuated estimates towards the expected null association (HR 1.24; 95% CI: 1.02–1.56). Of the 500 HDPS covariates selected in the final model, 46% were derived from test-related data.

**Conclusion:**

We empirically evaluated methods for incorporating laboratory test results into the HDPS framework, demonstrating their potential to reduce residual confounding in UK EHR studies. We propose principled approaches for incorporating laboratory test result data into data-driven confounding control strategies. Results from our case study highlight the potential for these data to reduce residual confounding in UK EHR studies. When available, test information should be considered within HDPS.

**Supplementary Information:**

The online version contains supplementary material available at https://doi.org/10.1186/s12874-026-02926-w.

## Background

The high dimensional propensity score (HDPS) procedure creates a scalable framework for data-driven confounder generation and selection in large healthcare databases [20, 23, 25]. Whilst data-driven covariates are usually derived from the presence of codes recorded in the healthcare database, this does not preclude the incorporation of other types of data in the HDPS procedure [20, 23, 37]. For example, Rassen et al. [19] investigated the use of free-text information within the HDPS framework.

Laboratory test results, including other routine clinical measurements (e.g. blood pressure (BP) measurements), are commonly available in electronic health records (EHRs) and, in comparison to administrative claims databases, can be available for a larger proportion of individuals [24]. There is increased availability of linked laboratory test result data in healthcare databases, which may include data on whether a particular test has been requested and the subsequent continuous test result value [18, 27]. However, they are rarely considered for confounder adjustment amid concerns surrounding the completeness and quality of data available [26]. Furthermore, inclusion of continuous values is not readily supported by the HDPS and how this framework extends to incorporate these data is unclear [25].

To address this gap, we make three methodological contributions to the HDPS framework by: (1) representing requested laboratory tests within HDPS data dimensions, (2) proposing multiple strategies for incorporating continuous test values, including via clinical cut-offs, and (3) empirically comparing the potential for each strategy to improve confounding control. The proposed methods are illustrated using a recent cohort study, which used UK EHR data to investigate the association between proton pump inhibitor (PPI) use and all-cause and cause-specific mortality.

### High-dimensional propensity scores

We briefly summarise the generic five steps of the HDPS procedure, below [25]:

- Step one, investigators specify the data structure. This involves identifying data dimensions capturing different aspects of care in the database under investigation (e.g. prescriptions).
- Step two, pre-exposure features within a specific assessment window are identified, and a prevalence filter is typically applied (e.g. selecting the top 200 most common features from each dimension). Features are usually in the form of codes and grouped at a specific granularity level (e.g. information on clinical diagnoses may be recorded using International Classification of Diseases, 10th edition (ICD-10) codes and truncated to the first three digits of this hierarchy).
- Step three, the recurrence of features is assessed in a pre-exposure covariate assessment window, creating binary covariates based on a set of frequency-based cut-offs [25]. These are defined as ‘Once’ if the feature is recorded ≥ once, ‘Sporadic’ if the feature is recorded ≥ median number of times, and ‘Frequent’ if the feature is recorded ≥ 75th percentile number of times.
- Step four, the large pool of HDPS covariates generated in step three is ranked by priority. The default method is to use values of the Bross formula, which uses univariate associations between the HDPS covariate and treatment/outcome to rank covariates based on their potential to bias the treatment-outcome relationship [25].
- Step five, a number of HDPS covariates (often the top 200 or 500) [20, 23] are selected to augment a set of investigator chosen confounders. This combined set of covariates is used to obtain estimated propensity scores. Standard propensity score methods (e.g., matching or weighting) are used to estimate treatment effects [35, 36].

### Incorporating types of laboratory test information

We describe types of laboratory test information available in large healthcare databases, describe their indication for use within confounder adjustment strategies and propose how they can be incorporated within the HDPS framework.

#### Tests-requested

The presence/absence of a request for a test is captured in many EHR datasets and can be incorporated into the HDPS. Whether a test is requested and conducted is usually captured by the presence/absence of a particular code(s). Requests for testing signals active contact and engagement of the patient with the healthcare system and is itself a marker of healthcare utilisation and potentially reflective of underlying health status.

The reason a specific test is requested relates to many factors, which are likely to vary depending on the specific test and the health condition of the patient. While certain tests have a specific indication, and will relate to either the monitoring of a current diagnosis or the potential discovery of a new diagnosis, other tests will not. For example, a test of the level of creatinine in the blood might be requested due to a potential or confirmed diagnosis of decreased kidney function. Conversely, cholesterol may often be routinely tested but not necessarily for a specific indication.

Data on the number of events recorded is incorporated as standard in the HDPS for other code-based data dimensions, for example, the presence or absence of a prescription code. To incorporate these data, we can apply the same steps for these other data dimensions. Namely, in Step 3, we define three binary covariates capturing whether a test is requested ≥ once, ≥ median number of times, and ≥ 75th percentile number of times. The consideration of these data across the other steps of the HDPS procedure is unchanged.

#### Continuous test results

Continuous test result values may more directly reflect the underlying health status of an individual. However, the use of these data within the HDPS framework (and confounder adjustment strategies more generally) is complicated by two key issues.

First, these data are not automatically checked for implausible or impossible values when entered into the database and results for a given test may be recorded using a range of heterogeneous measurement units. Data cleaning is typically required to remove unlikely values and harmonise the units before test result values can be incorporated into an analysis [34].

The second issue relates to missing data. For continuous test values, data is missing either because the test has not been requested, or because the result is not provided, or is implausible. So, whilst each person has an underlying biological value, these are only observed in people for whom a test is conducted to ascertain (with measurement error) this value [7, 26]. As with missing data generally, consideration of possible missingness mechanisms is key to ensure appropriate analysis [5, 28].

Before incorporating continuous test result values, it is necessary to remove implausible values and harmonise the units of measurement to obtain a set of cleaned values. This can be achieved by applying reference ranges used in clinical practice (as presented in our Case Study). For an individual, the closest cleaned value prior to cohort entry will likely be most relevant to capturing current health status and the treatment decision of interest. However, this could be extended to handle repeated instances of the same test for an individual over time, where appropriate.

We propose defining an additional data dimension identifying the most recent cleaned test value for each specific test across a covariate assessment window defined on or prior to cohort entry (Step 1). We consider two ways to incorporate these data into the HDPS procedure.

First, we propose generating cut-offs based on whether an individual’s most recent test result falls within a reference range (Step 3). This generates a set of binary covariates, conceptually consistent with the standard HDPS approach, that can be prioritised and selected from in the usual way.

Second, since the dichotomisation of continuous variables reduces statistical power and can result in residual confounding [3, 9, 22, 29], we also consider continuous modelling. For any dichotomised test result highly ranked by the Bross formula (in the case study, this was defined as in the top 100), we propose additionally including the corresponding continuous test value (in a linear form) and a missing indicator in the HDPS model. For continuous variables, the missing indicator approach involves setting missing values to a(ny) fixed value, for example 0 and defining a binary missingness indicator variable [2], [8].

The proposed approach leverages work by Blake et al. [2] showing that the missing indicator approach is unbiased under the assumption that the underlying biological value only contributes to the treatment decision if it is measured, i.e. the missing value does not inform the decision to initiate treatment. This is often likely to hold (at least approximately) when considering many laboratory test result values since the prescribing clinician will not have a value recorded to inform the treatment decision. Furthermore, the approach is particularly valuable in this setting since the recording of some test values will be sparse and a complete case analysis, where only the subset of individuals with complete data on a set of continuous test result values, is likely to substantially reduce the sample size and could induce selection bias via M-bias or selection on effect measure modifiers [5, 12]. Multiple imputation (MI) is an alternative method and is widely used approach in other settings [5]. However, it poses several challenges in this context. Firstly, MI is valid under the missing at random assumption, which may be less plausible for laboratory values, especially if individuals with low or high levels are monitored [17]. Secondly, combining MI with the propensity score framework has additional complexities,these would be further complicated by nesting this within a data-driven variable selection procedure [11]. Finally, MI would also require correct specification of an imputation model for each laboratory test result under consideration.

### Application to case study

#### Summary

To illustrate the proposed methods, we used data from a study using the UK Clinical Practice Research Datalink (CPRD) GOLD database of primary care records linked to Office of National Statistics (ONS) death registration data, hospital admission data recorded in Hospital Episode Statistics Admitted Patient Care (HES APC) and Index of Multiple Deprivation (IMD) socioeconomic deprivation data [4]. The comparison of interest compared the hazard of all-cause and cause-specific mortality between new-users of PPIs and new-users of H_2_ receptor antagonists (H2RAs), two medications used for gastric acid suppression. Study entry was defined as first prescription date for PPI and H2RA users within the eligibility window, starting 2nd January 1998. Individuals were followed up until the earliest of death date, deregistration from general practice, date of last practice data collection, 17th April 2017 (end of linkage coverage), or date of first PPI prescription (H2RA users only).

On the basis of the study results, and given differences in prescribing of H2RAs and PPIs, Brown et al. [4] concluded that baseline differences between comparator groups were likely not fully accounted for, despite adjustment for HDPS covariates capturing clinical, referral and prescription information.

We investigated the ability of information relating to laboratory tests to further improve confounding control, focussing on the Chronic Obstructive Pulmonary Disorder (COPD) mortality, an outcome where elevated hazard ratios (HR) were observed across investigator-led and HDPS analyses despite a causal effect of PPI treatment being considered unlikely to based on disease [4]. The hypothesis of residual confounding was further supported by a negative control outcome analysis which observed no evidence of an association between PPI use and accidental trauma mortality (excluding falls), and the COMPASS trial which found no evidence of increased risk of COPD mortality in PPI users [14].

#### Laboratory test information processing

Test requests were identified using the Read coding system. In CPRD GOLD, Read codes successfully captured distinct tests so mapping or truncation of codes was not required.

For the continuous test results, we identified all values for 35 commonly recorded blood tests in the 1-year prior to cohort entry. We applied previously developed cleaning rules to harmonise units of measurement and remove implausible values [13, 15]. The distribution of each was compared before and after the cleaning rules were applied.

For a given individual and blood test, we selected the cleaned value closest to cohort entry. Where multiple cleaned values existed for an individual the lowest test result was selected; this decision could be investigated in future work.

In the context of blood tests, reference ranges are either bounded in one direction or can plausibly take values either side of the specified interval. Cut-offs were therefore defined as follows. Where the reference range was bounded in one direction, we generated a single binary covariate capturing whether the test result value was within this reference range. Where the reference range was not bounded in one direction, we defined 3 binary covariates, representing: low (test value is below reference range), normal (test value within reference range) and high (test result value is above reference range). These cut-offs (summarised in Table 1) were specified based on reference ranges published by NHS Scotland and additionally checked by one of the authors—DRM [16]. Note, some are stratified by age or sex and this was incorporated when defining the variables.

**Table 1:** Cut-offs for generating binary test result HDPS covariates in case study

Test	Normal range definition	Number of binary covariates
Blood Pressure (BP)		
Systolic Blood Pressure	Age < 80: < 140 mmHg	1
	Age ≥ 80: < 150 mmHg	1
Diastolic Blood Pressure	< 90 mmHg	1
Calcium		
Calcium	2.15–2.65 mmol/L	3
Calcium Adjusted	2.20–2.60 mmol/L	3
Full Blood Count (FBC)		
Basophil	0–0.1 × 10^9^/L	1
Eosinophil	0–0.4 × 10^9^/L	1
Haemoglobin	Men: 135–180 g/L	3
	Women: 115–160 g/L	3
Lymphocyte	1.0–4.8 × 10^9^/L	3
Monocytes	0.2–0.8 × 10^9^/L	3
MCV^*^	80–100 fL	3
MCH^*^	27–34 pg	3
Platelet**s**	130–400 K/μL	3
RBC^*^	Men: 4.5–6.0 × 10^12^/L	3
	Women: 4.0–5.6 × 10^12^/L	3
WBC^*^	4.0–11.0 × 10^9^/L	3
Glucose		
Glucose	3.3–6.1 mmol/L	3
Glucose Fasting	3.3–6.1 mmol/L	3
Lipids		
Cholesterol	≤ 5 mmol/L	1
HDL Cholesterol	≥ 1 mmol/L	1
LDL Cholesterol	≤ 3 mmol/L	1
Triglycerides	0.85–2.0 mmol/L	3
Liver Function Tests (LFTs)		
Albumin	38–50 g/L	3
AST^*^	15–42 IU/L	3
ALT^*^	0–40 U/L	1
ALP^*^	Age: 17—60: 35–115 U/L	3
	Age: ≥ 60: 35–150 U/L	3
AKP^*^	Age: 17—60: 35–115 U/L	3
	Age: ≥ 60: 35–150 U/L	3
Bilirubin	2–20 μmol/L	3
Urea and electrolytes		
Creatinine	Age < 60: 20–120 μmol/L	3
	Age ≥ 60: 70–140 μmol/L	3
Potassium	3.6–5.4 mmol/L	3
Sodium	134–144 mmol/L	3
Urea	Age < 60: 3.0–8.5 mmol/L	3
	Age ≥ 60: 3.0–10.0 mmol/L	3
GFR^*^	≥ 60 mls/min	1
Other		
C-reactive Protein Test	< 5 mg/L	1
HbA1c	< 60 mmol/mol	1
Total Protein	60–80 g/L	3
Urate	Men: 0.12–0.42 mmol/L	3
	Women: 0.12–0.38 mmol/L	3

*Abbreviations*: *HDPS* High-dimensional propensity score

^*^Glomerular filtration rate (GFR), Alkaline Phosphatase (AKP), Alkaline Phosphatase Level (ALP), Alanine Aminotransferase (ALT), Aspartate Aminotransferase (AST), Mean Corpsucular Volume (MCV), Mean Corpsucular Haemoglobin (MCH), Red Blood Count (RBC), White Blood Count (WBC)

#### Statistical analysis

As with the original study, all analyses estimated HRs targeting the average treatment effect in the treated (ATT) using standardised mortality ratio weighted Cox regression models [4, 6]. For all analyses, we inspected the propensity scores distributions and assessed covariate balance before/after weighting using standardised mean differences (SMDs), with values < 0.1 taken to indicate good balance.

The investigator analysis estimated propensity scores using logistic regression based on a set of pre-defined covariates. Our primary HDPS analysis follows our previously proposed approach for CPRD GOLD with HDPS parameters from the original study [4, 30]. Defining Clinical, Referral and Prescription data dimensions, we identified the top 200 most prevalent codes from each dimension and selected the top 500 Bross formula-ranked covariates to augment the investigator-specified propensity score model.

**Fig. 1:**
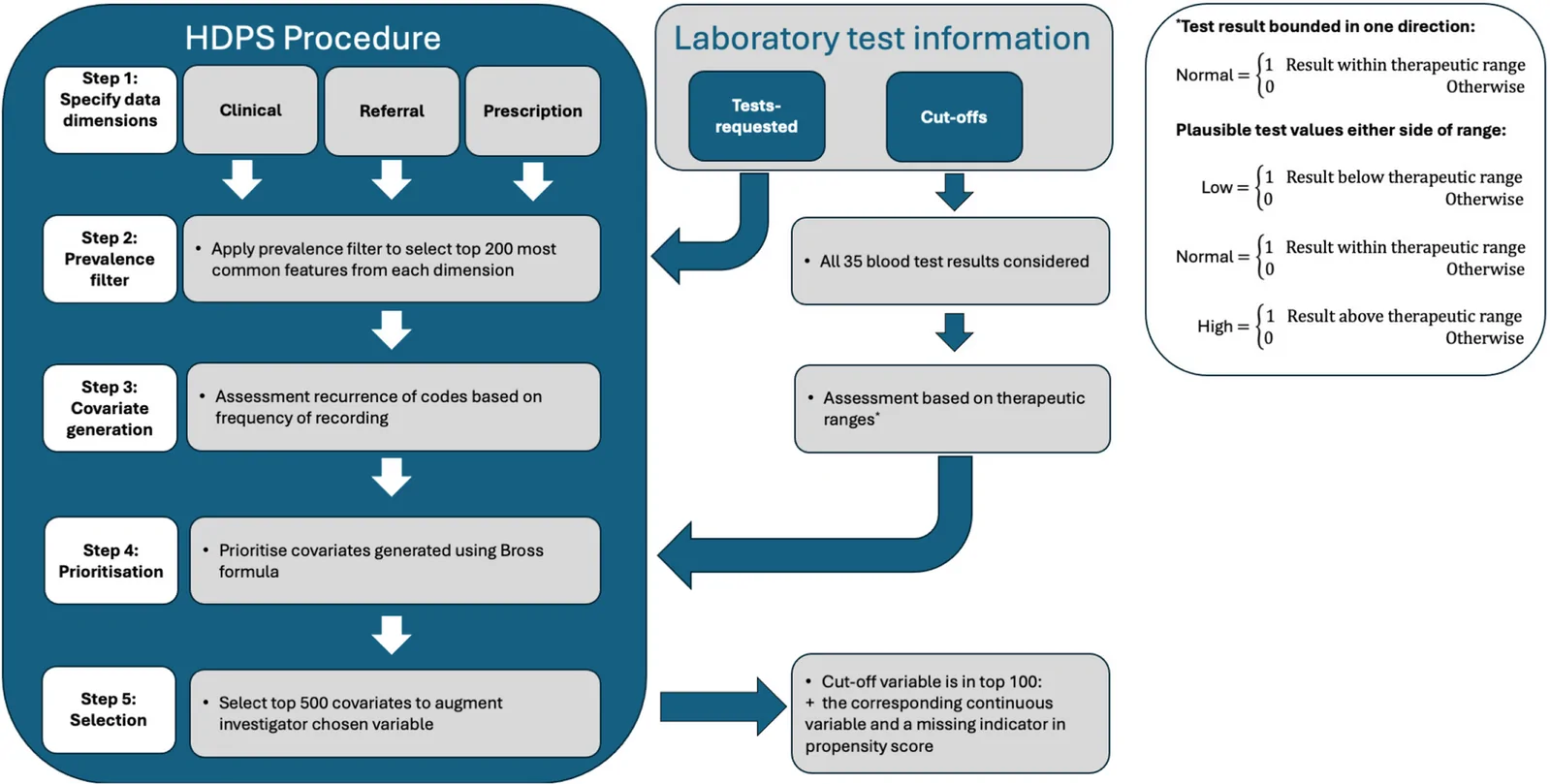
Summary of HDPS procedure and integration of laboratory test information. Abbreviations: HDPS; high-dimensional propensity score

We incorporated testing data sequentially, as follows. First, we included a test-requested dimension, capturing all tests requested in the 1-year prior to cohort entry (labelled ‘test-requested’). Second, we additionally generated a set of binary variables defined by cut-offs applied to the available blood test information (labelled ‘cut-offs’ dimension) and added these for prioritisation at Step 4 of the HDPS procedure. Third, for each test with a cut-off indicator selected in the top 100 HDPS covariates, we additionally incorporated the standardised continuous test result variable linearly and a missing indicator in the propensity score model. Consistent with the original study, we considered the top 500 Bross formula-ranked covariates to be our primary analysis. At each stage, we investigated the robustness of results to the number of covariates selected and used diagnostics to characterise the types of covariates selected [31]. We additionally described the univariable associations between the testing variables and both treatment and outcome status. Implementation of these approaches alongside the standard HDPS procedure is summarised in Fig. 1.

All analyses were conducted using Stata 17 [32]. Analytical code for implementing the approaches presented is available at https://github.com/johntaz/HDPS-Test-Results.

## Results

The study cohort included 733,885 new users of PPIs and 124,410 new users of H2RAs. Of these individuals, 7,846 (1.1%) PPI users and 538 (0.4%) of H2RA users had COPD-specific mortality. A summary of the results from the original study and a HDPS analysis not considering any test information is presented in Table 1. Briefly, compared to H2RA users, PPI users tended to be older (mean age 54.9 years vs 51.2 years in H2RAs) and had a higher prevalence of comorbidities and medication use at baseline [4]. The crude HR comparing COPD-mortality between PPI and H2RA users without weighting was 1.83 (95% CI: 1.65–2.03). The HR from the investigator-specified analysis was 1.37 (95% CI: 1.14–1.66). Results from the primary HDPS analysis (incorporating clinical, referral and prescription information) were largely consistent, with the addition of 500 HDPS covariates resulting in a HR of 1.36 (95% CI: 1.14–1.64).

### Tests requested

Initially, we incorporated test result information by adding an additional data dimension capturing tests requested in the year prior to cohort entry.

We identified a total of 1,060 unique test codes within the covariate assessment period. A summary of the top 50 most prevalent tests requested is provided in Appendix Table A1. We observed that a high proportion of patients have test information recorded in the 1-year prior to cohort entry, with 50% of the study population having at least one test-requested for the top 20 tests.

In the primary analysis, selecting the top 500 HDPS covariates, 38% of the covariates selected originated from the test-requested dimension (including 53 out of the top 100 covariates) (Table 2).

**Table 2:** Summary of HDPS analysis, without test information dimension, for association between PPI prescription and COPD-mortality among PPI and H2RA users

Analysis	Number of variables included	Hazard ratio (95% CI)
Unweighted	-	1.83 (1.65—2.03)
Investigator-specified	35	1.37 (1.14—1.66)
HDPS Analysis^*^	+ 100	1.37 (1.14—1.65)
	+ 250	1.35 (1.13—1.63)
	+ 500	1.36 (1.14—1.64)
	+ 750	1.39 (1.16—1.67)

*Abbreviations*: *HDPS* High-dimensional propensity score, *CI* Confidence interval

^*^Based on Clinical, Referral and Prescription data dimensions, identifying the top 200 most prevalent codes from each dimension and selecting the top Bross formula-ranked covariates to augment the investigator-specified propensity score model

**Fig. 2:**
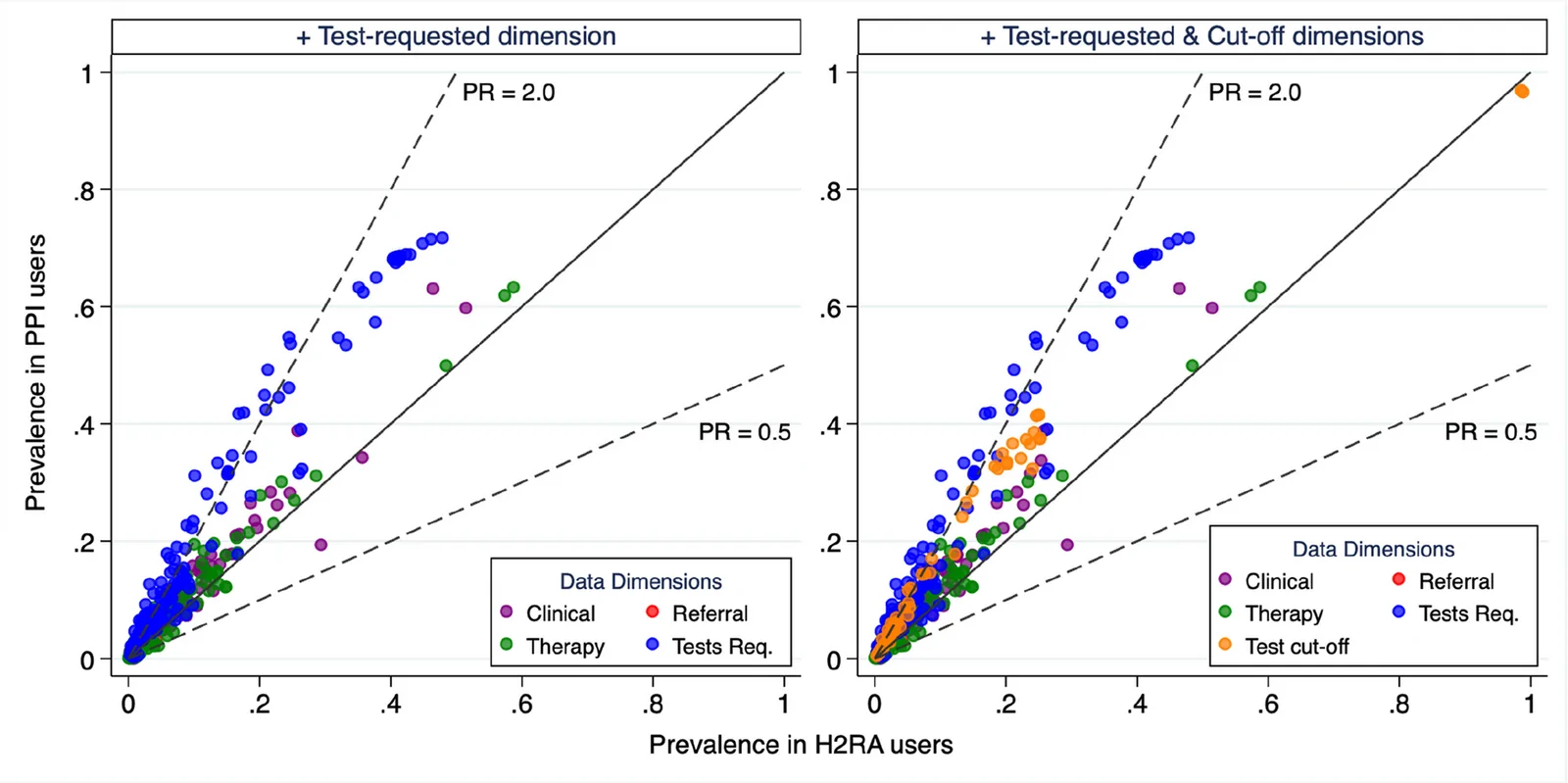
Prevalence of the top 500 Bross-ranked HDPS covariates by treatment group. All HDPS models contain the clinical, referral and prescription dimensions, plus the corresponding test-based dimensions (with test data assessed 1-year prior to cohort entry). Abbreviations: PR, prevalence ratio; PPI, proton pump inhibitor; H2RA, H_2_ receptor antagonist

**Fig. 3:**
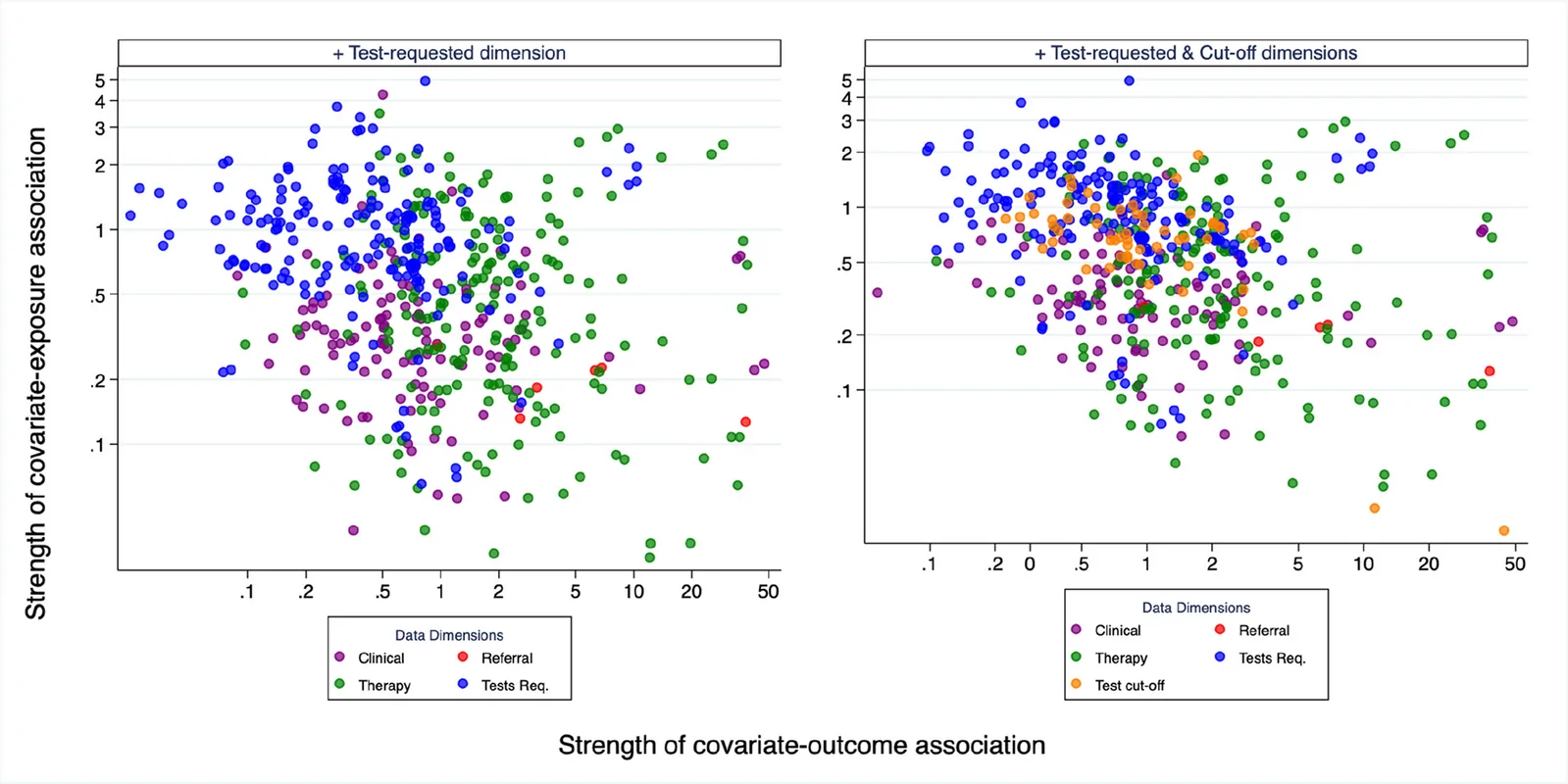
Comparison of the covariate-exposure and covariate-outcome associations for the top 500 Bross-ranked HDPS covariates. All HDPS models contain the clinical, referral and prescription dimensions, plus the corresponding test-based dimensions (with test data assessed 1-year prior to cohort entry). Abbreviations: Tests Req., Tests-requested

Compared to the primary HDPS model without considering test information (HR 1.36; 95% CI: 1.14—1.64), incorporation of the test-requested information appeared to further contribute to confounding control and obtained results closer to the expected null association (HR 1.25; 95% CI: 1.01—1.54). Figure 2 highlights that the covariates based on tests requested were typically more prevalent in PPI users compared to the H2RA users. These differences could be due to poorer health of PPI users, who had more comorbidities when examining investigator-specified covariates or due to calendar differences in the start of therapy; PPI initiation occurred more frequently in later periods where data may better capture current health status. Figure 3 highlights several covariates from the test-requested dimension with strong outcome associations and these all relate to respiratory tests and include: forced expiratory volume tests, forced vital capacity tests and spirometry tests. Descriptions for the top 50 HDPS covariates included in this model are presented in Appendix Table A2.

Despite the larger pool of covariates, in sensitivity analyses, selecting 750 covariates did not alter the estimates substantially (Table 3).

**Table 3:** Summary of HDPS analyses incorporating laboratory test information. All analyses contain the investigator covariates and consider HDPS covariates from the clinical, referral and prescription dimensions plus the corresponding test-based information

Number of variables included	** + Test-requested dimension**^**a**^ **HR (95% CI)**	** + Test-requested and cut-off dimensions**^**b**^ **HR (95% CI)**	** + Test-requested, cut-off dimensions and continuous test variables**^**c**^ **HR (95% CI)**
100	1.36 (1.12—1.66)	1.36 (1.11—1.68)	1.35 (1.10—1.67)
250	1.34 (1.09—1.63)	1.30 (1.05—1.62)	1.32 (1.06—1.64)
500	1.25 (1.01—1.54)	1.24 (1.00—1.54)	1.24 (1.00—1.54)
750	1.25 (1.00—1.55)	1.21 (0.97—1.52)	1.22 (0.97—1.53)

*Abbreviations*: *HDPS* High-dimensional propensity score, *HR* Hazard ratio, *CI* Confidence interval

^a^Test-requested dimension refers to the dimension capturing all tests requested in the 1-year prior to cohort entry

^b^Cut-off dimension refers to the dimension capturing the binary cut-off variables derived from the available blood test information

^c^Continuous test variables are included for any test result where a derived binary cut-off variable was selected in the top 100 Bross-ranked HDPS covariates

### Continuous test results

We applied previously developed cleaning rules to minimise implausible values and harmonise the units of measurement [15]. The proportion of patients with an eligible continuous test for each test in the 1-year prior to cohort entry is presented in Appendix Table A3. Raw and cleaned distributions for all the blood test results across the 1-year covariate assessment window are included in Appendix 2. The marked improvement across these distributions highlights the importance of this preliminary cleaning step and the risks of naïvely including raw measurements from EHR data.

These values were included as an additional data dimension alongside the clinical, referral, prescription and tests-requested dimensions. We generated the aforementioned reference-based binary cut-off variables and added these to the set to be prioritised in Step 4 of the HDPS procedure. In the primary analysis, selecting the top 500 HDPS covariates, 46% related to test-related covariates (35% from the test-requested dimension and 11% from the cut-offs dimension).

Compared to the HDPS model incorporating the tests requested dimension, additionally incorporating the blood test cut-offs obtained similar results (HR 1.25; 95% CI: 1.01–1.55). The covariates based on the cut-offs were typically more prevalent in PPI users compared to the H2RA users (Fig. 2). Furthermore, Fig. 3 highlights two covariates from the cut-offs dimension with strong outcome associations and these relate to patients with normal recordings for glomerular filtration rate and high-density lipoproteins. Descriptions for the top 50 HDPS covariates included in this model are presented in Appendix Table A4. In sensitivity analyses, the results were robust to the number of HDPS covariates selected.

For the blood test cut-offs selected in the top 100 HDPS covariates, we additionally incorporated the full continuous variable and a missing indicator. This resulted in the inclusion of 19 continuous blood test variables (listed in Appendix 3). Including these alongside the top 500 HDPS covariates, did not additionally alter the results, HR 1.24 (95% CI: 1.02—1.56) (Table 3).

**Fig. 4:**
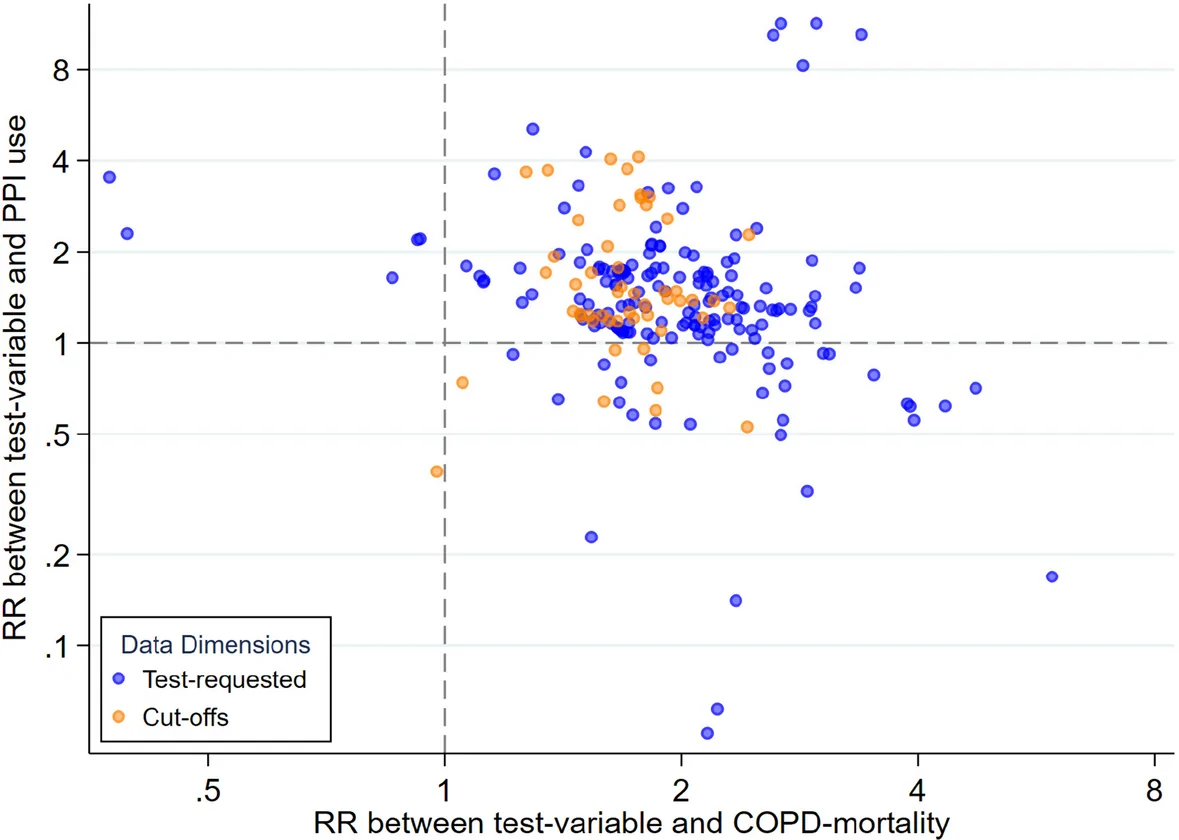
Univariable relative risks between test-variables and both PPI use and outcome status for the top 500 Bross-ranked HDPS covariates. Abbreviations: Tests Req., Tests-requested; RR, Relative risk; COPD, Chronic Obstructive Pulmonary Disorder; PPI, Proton Pump Inhibitor

Finally, Fig. 4 presents univariable relative risks for the test-based variables. Across both test-requested and cut-off variables, testing was predominately positively associated with PPI use and COPD mortality.

### Propensity score diagnostics

Across all analyses, propensity score distributions demonstrated good overlap between the treatment groups and were consistent across the HDPS analyses considered (see Appendix 4, Figures A1-A5).

Propensity score models achieved good covariate balance, with SMDs below the threshold of 0.1 for all analyses (see Appendix 5, Figures A6-A10). In the HDPS analyses incorporating the test-requested and test cut-off dimensions, a greater number of variables exhibited large imbalances (SMD > 0.4) in the unweighted populations compared to those observed in the investigator-specified analysis. Successfully balancing these variables in the HDPS analyses likely contributed to the observed attenuation in treatment effect estimates with increasing adjustment for test-related variables.

## Discussion

We have addressed a key gap in the established HDPS framework by proposing and demonstrating principled approaches for incorporating laboratory test information, using the association between PPI use and COPD mortality as an illustrative example. Compared to a HDPS model incorporating clinical, referral and prescription information (HR 1.36; 95%: CI 1.14—1.64), the model incorporating all available test information (relating to tests requested, test cut-offs and continuous test variables) appeared to improve confounder adjustment and obtained results closer to the expected null association (HR 1.24; 95% CI: 1.02—1.56). Importantly, the standard HDPS did not result in different estimates relative to the investigator-specified propensity score model and only with the additional test covariates was the association attenuated, indicating potential residual confounding in the primary estimates. Furthermore, 46% of the top 500 covariates in the final model incorporating test information were derived from test-related data dimensions, highlighting the potential importance of these data for mitigating confounding bias in UK EHRs. Despite this, in this example, residual confounding is likely to remain, possibly due to unmeasured factors such as frailty and disease severity of PPI users [4]. Therefore, whilst consideration of a diverse range of data within the HDPS procedure can improve confounding control, it will not necessarily eliminate all sources of confounding if proxies of important confounders are not captured within the healthcare database.

### Comparison with previous work

The work presented extends the existing HDPS framework to consider test result information in two novel ways [25]. First, we have demonstrated how relatively simple methods, such as reference-based cut-offs can be used to incorporate continuous laboratory test data within the HDPS framework in a way that is consistent with the semi-automated nature of this approach. Second, we have proposed an approach to incorporate continuous variables using missing indicators, which is valid under assumptions that are plausible in large healthcare databases [2]. In this example, adding tests requested had the largest influence on estimates, highlighting the potential for these variables to capture differences in healthcare utilisation, clinical contact frequency, and disease severity not necessarily reflected in the test results themselves. The performance of the adapted framework and importance of different types of test information will vary by setting, likely depending on the treatment (e.g. acute vs. chronic exposures), outcome, confounding structure, and richness of data available. Application of the framework in other settings will increase our understanding of when these data can contribute to confounding control most meaningfully.

### Limitations

This work has highlighted several issues which could benefit from further exploration. Firstly, whilst we have focused on the inclusion of data from 35 commonly recorded blood tests, the developed framework could be expanded to incorporate additional test data, for example, spirometry results [21]. This could lead to creation of a standard battery of commonly recorded tests for consideration in the HDPS procedure. Secondly, increasing the number of data dimensions leads to a larger pool of covariates for prioritisation and selection. In this example, results were robust to the number of covariates selected, however, how to identify the optimal number of covariates to incorporate is unclear [10, 23]. Our proposal could also be considered in conjunction with machine-learning augmented variable selection procedures [10], which may offer opportunities to capture important non-linearities in the continuous variables considered. Generalisations of the Bross formula to accomodate continuous variables could also be considered for ranking of these covariates, under additional assumptions [33]. Thirdly, when selecting the cut-off covariates, we only incorporated the categories selected by the Bross formula i.e. if the ‘High’ variable was selected the ‘Low’ or ‘Normal’ variables were not necessarily included (unless independently selected). Future work could explore the selection of all categories if one of the categories for a certain test is selected. Finally, we focussed on including continuous variables in a linear form in the propensity score model. Whilst this approach can lead to residual confounding, it can perform well in many settings [9]. Future work could investigate the use of fractional polynomials and splines for more precise modelling of the relationship between treatment initiation and these continuous test variables [1, 29]. However, in this example, the use of cubic splines led to issues surrounding fitting of the propensity score models.

We used missing indicators to handle missing data arising from the incorporation of the continuous test results, highlighting that, despite previous criticism of this method in the context of incorporating test data for confounding control [26], it may prove a useful approach [2]. Whilst MI has gained popularity in other contexts and may seem an appealing alternative [5], it would add several complexities in this setting. For example, combining MI and propensity score models is not straightforward [11] and MI would also lead to specific issues surrounding increased computational burden and the need to specify imputation models [28]. Finally, despite a lot of methodological work in the context of HDPS [23], [20], implications surrounding the use of missing data methods when applying the HDPS are not well understood, and this highlights an important area for future research.

## Conclusion

In summary, we empirically investigated several methods for incorporating test results in the HDPS framework. Our study illustrates the potential for laboratory test result information to improve the ability of HDPS approaches to reduce residual confounding in UK EHRs. In any application of the HDPS, the performance will be constrained by the data available within the defined data dimensions. Therefore, whilst the incorporation of laboratory test data will help to minimise residual confounding arising from these factors (and associated proxies), residual confounding may remain from unrelated unmeasured factors.

## Supplementary Information

Supplementary Material 1.

## Data Availability

No additional data is available. Data management and analysis code are available on our GitHub repository: https://github.com/johntaz/HDPS-Test-Results

## Abbreviations

ATT: Average treatment effect in the treated
COPD: Chronic obstructive pulmonary disorder
CPRD: Clinical practice research datalink
EHR: Electronic health record
H2RA: H2-receptor antagonists
HES APC: Hospital episode statistics admitted patient care
HR: Hazard ratio
HDPS: High-dimensional propensity score
ICD-10: International classification of diseases, 10th edition
IMD: Index of multiple deprivation
MI: Multiple imputation
PPI: Proton pump inhibitor
SMD: Standardised mean differences
UK: United kingdom
