# Supplementary material for: Methods for incorporating test result information within the high-dimensional propensity score framework: application in UK electronic health record data
Source: BMC Med Res Methodol. 2026 Jun 30;26:208. doi: 10.1186/s12874-026-02926-w (PMC13584297; doi:10.1186/s12874-026-02926-w)

Appendix

Appendix 1: Descriptives of laboratory test information

Table A1: Read codes for the top 50 tests-requested in the case study

Read code	Description	Total*
423..00	Haemoglobin estimation	585,841
42P..00	Platelet count	582,052
42A..00	Mean corpuscular volume (MCV)	575,489
4433.00	Serum creatinine	559,046
42H..00	‘Total white cell count	558,474
426..00	Red blood cell (RBC) count	555,576
42M..00	Lymphocyte count	553,596
424.00	Neutrophil count	553,413
42N..00	Monocyte count	551,326
4415.00	Serum sodium	550,262
4414.00	Serum potassium	550,101
42K..00	Eosinophil count	549,551
428..00	Mean corpusc. haemoglobin(MCH)	546,025
421.00	Basophil count	523,588
44M4.00	Serum albumin	508,079
44F..00	Serum alkaline phosphatase	502,823
424..00	Full blood count - FBC	467,489
429..00	Mean corpusc. Hb. conc. (MCHC)	441,299
44P..00	Serum cholesterol	433,481
4258.00	Haematocrit	432,365
4439.00	Serum urea level	424,306
442W.00	Serum TSH level	387,705
44G3.00	ALT/SGPT serum level	369,175
44Q..00	Serum triglycerides	355,280
44P5.00	Serum HDL cholesterol level	355,196
44M3.00	Serum total protein	337,372
44D6.00	Liver function test	329,489
44EC.00	Serum total bilirubin level	327,003
4286.00	Erythrocyte sedimentation rate	319,506
4227.00	Red blood cell distribution width	275,417
44JB.00	Urea and electrolytes	273,437
466..00	Urine test for glucose	269,767
467..00	Urine protein test	264,306
44g..00	Plasma glucose level	261,667
44P6.00	Serum LDL cholesterol level	252,907
4418.00	Serum calcium	251,460
440..00	Serum lipids	248,893
451E.00	GFR calculated abbreviated MDRD	241,175
44E..00	Serum bilirubin level	226,428
441C.00	Corrected serum calcium level	220,891
44M5.00	Serum globulin	205,394

44G9.00	Serum gamma-glutamyl] transferase level	183,860
442V.00	Serum free T4 level	177,675
4419.00	Serum inorganic phosphate	174,968
535..00	Standard chest X-ray	156,372
44U..00	Blood glucose result	150,787
44PF.00	Total cholesterol:HDL ratio	148,586
442J.00	Thyroid function test	148,298
442A.00	TSH - thyroid stim. hormone	147,971
44CS.00	Serum C reactive protein level	138,351

[*] Total represents the number of patients with at least one test requested in the 1-year prior to cohort entry

Table A2: Top 50 HDPS covariates from the analysis considering the tests-requested dimension

Rank	Dimension	Code-Suffix	Description
1	Test-requested	Once	Peak expiratory flow rate measured using EN 13826 device
2	Clinical	Once	Other chronic obstructive pulmonary disease
3	Test-requested	Once	Forced vital capacity - FVC
4	Test-requested	Once	Spirometry
5	Prescription	Once	Antimuscarinic Bronchodliators
6	Test-requested	Once	FEV1/VC percent
7	Test-requested	Once	FEV1/FVC ratio after bronchodilator
8	Prescription	Once	Compond Bronchodilator Preparations
9	Test-requested	Once	Standard chest X-ray
10	Prescription	Spor	Antimuscarinic Bronchodliators
11	Clinical	Once	Personal history of certain other diseases
12	Test-requested	Once	Serum creatinine
13	Test-requested	Once	Serum potassium
14	Clinical	Once	Unspecified chornic bronchitis
15	Test-requested	Once	Serum sodium
16	Clinical	Once	Hypersensitivity pneumonitis due to organic dust
17	Clinical	Once	Cough
18	Clinical	Once	Heart Failure
19	Prescription	Freq	Antimuscarinic Bronchodliators
20	Prescription	Once	Osmotic Diuretics
21	Prescription	Once	Use of Corticosteroids
22	Prescription	Once	Antiplatelet Drugs
23	Test-requested	Once	Serum alkaline phosphatase
24	Test-requested	Once	Peak exp. Flow rate: PEFR/PFR
25	Test-requested	Once	Serum albumin
26	Prescription	Once	Mucolytics
27	Test-requested	Once	Liver function test
28	Test-requested	Once	Urea and electrolyes
29	Clinical	Once	Mental and behavioural disorders due to use of tobacco
30	Test-requested	Freq	Serum creatinine
31	Test-requested	Freq	Serum sodium

32	Test-requested	Freq	Serum potassium
33	Prescription	Once	Calcium-channel Blockers
34	Prescription	Once	Selective Beta2-Agonists
35	Test-requested	Once	Full blood count - FBC
36	Prescription	Once	Loop Diuretics
37	Prescription	Once	Lipid-Regulating Drugs
38	Prescription	Once	Opiod Analgesics
39	Prescription	Once	Bisphosphonates and Other Drugs
40	Test-requested	Once	Serum calcium
41	Test-requested	Freq	Serum urea level
42	Test-requested	Once	PEFR - peak exp. Flow rate
43	Prescription	Once	Theophylline
44	Test-requested	Once	Serum urea level
45	Prescription	Once	Vitamin D
46	Prescription	Once	Other Andrenoceptor Agnoists
47	Test-requested	Once	Serum cholesterol
48	Test-requested	Once	Chest X-ray - routine
49	Test-requested	Once	Prostate specific antigen
50	Prescription	Once	Corticosteroids (Respiratory)

Table A3: Summary of the 35 blood test results with the proportion of PPI and H2RA users with a cleaned and eligible continuous value

Blood test Result	Cleaning rules applied	Percentage with result within 1 year of cohort entry (%)
AKP*	Yes	36
ALP*	No	31
ALT*	Yes	31
AST*	Yes	8
Albumin	Yes	37
Basophil	Yes	22
Bilirubin	Yes	37
CRP*	Yes	11
Calcium	Yes	14
Calcium Adjusted	Yes	13
Cholesterol	Yes	29
Creatinine	Yes	43
Diastolic Blood Pressure	Yes	95
Eosinophil	Yes	36
GFR*	Yes	19
Glucose	Yes	22
Glucose Fasting	Yes	8
HDL*	Yes	23
Haemoglobin	Yes	40
HbA1c	Yes	8
LDL*	Yes	19
Lymphocyte	Yes	37
MCH*	No	36
MCV*	Yes	39
Monocytes	Yes	37
Platelets	Yes	39
Potassium	Yes	42
RBC*	Yes	1
Sodium	Yes	42
Systolic Blood Pressure	Yes	95
Total Protein	No	24
Triglycerides	Yes	22
Urate	Yes	1
Urea	Yes	34
WBC*	Yes	38

*Glomerular filtration rate (GFR), Alkaline Phosphatase (AKP), Alkaline Phosphatase Level (ALP), Alanine Aminotransferase (ALT), Aspartate Aminotransferase (AST), Mean Corpsucular Volume (MCV), Mean Corpsucular Haemoglobin (MCH), Red Blood Count (RBC), White Blood Count (WBC)

Table A4: Top 50 HDPS covariates from the analysis considering the tests-requested dimension and test cut-offs dimension

Rank	Dimension	Code-Suffix	Description
1	Test-requested	Once	Serum potassium
2	Test-requested	Once	Serum sodium
3	Test-requested	Once	Peak expiratory flow rate measured using EN 13826 device
4	Test-requested	Once	Serum creatinine
5	Test-requested	Once	Serum albumin
6	Test-requested	Once	Serum alkaline phosphatase
7	Test-requested	Once	Monocyte count
8	Test-requested	Once	Read blood cell (RBC) count
9	Test-requested	Once	Total white cell count
10	Test-requested	Once	Lymphocyte count
11	Test-requested	Once	Neutrophil count
12	Test-requested	Once	Mean corpuscular volume (MCV)
13	Test-requested	Once	Platelet count
14	Test-requested	Once	Eosinophil count
15	Test-requested	Once	Mean corpusc. Haemoglobin (MCH)
16	Test-requested	Once	Serum urea level
17	Test-requested	Once	Basophil count
18	Test-requested	Once	Haemoglobin estimation
19	Test-requested	Once	Liver function test
20	Test-requested	Once	Forced vital capacity - FVC
21	Test-requested	Once	Full blood count - FBC
22	Clinical	Once	Other chronic obstructive pulmonary disease
23	Test-requested	Once	Spirometry
24	Clinical	Once	Personal history of certain other diseases
25	Test-requested	Once	Serum cholesterol
26	Test-requested	Once	Haematocrit
27	Test-requested	Once	Mean corpusc. Hb. Conc. (MCHC)
28	Prescription	Once	Antimuscarinic Bronchodliators
29	Cut-offs	Normal	Potassium
30	Test-requested	Once	ALT/SGPT serum level
31	Test-requested	Once	Standard chest X-ray
32	Test-requested	Once	Serum TSH level

33	Clinical	Once	Persons encountering health services for other coneslling and medical advice, not elswhere classified
34	Cut-offs	Normal	Sodium
35	Test-requested	Once	Urea and electrolyes
36	Test-requested	Once	FEV1/VC percent
37	Test-requested	Once	Serum total protein
38	Test-requested	Once	FEV1/FVC ratio after bronchodilator
39	Prescription	Once	Compond Bronchodilator Preparations
40	Test-requested	Once	Serum HDL cholesterol level
41	Cut-offs	Normal	Bilirubin
42	Prescription	Spor	Antimuscarinic Bronchodliators
43	Test-requested	Once	Serum total bilirubin level
44	Clinical	Once	Cough
45	Test-requested	Once	Serum triglycerides
46	Clinical	Once	Unspecified chornic bronchitis
47	Test-requested	Once	Serum calcium
48	Cut-offs	Normal	Alkaine phosphatase
49	Prescription	Once	Osmotic Diuretics
50	Clinical	Once	Hypersensitivity pneumonitis due to organic dust

Appendix 2: Distribution of blood test results before and after cleaning and unit harmonisation rules were applied

Test results in the year prior to index

Calcium

After cleaning, 120244 (14.01%) of patients have a measurement of calcium in the 1 year prior to index

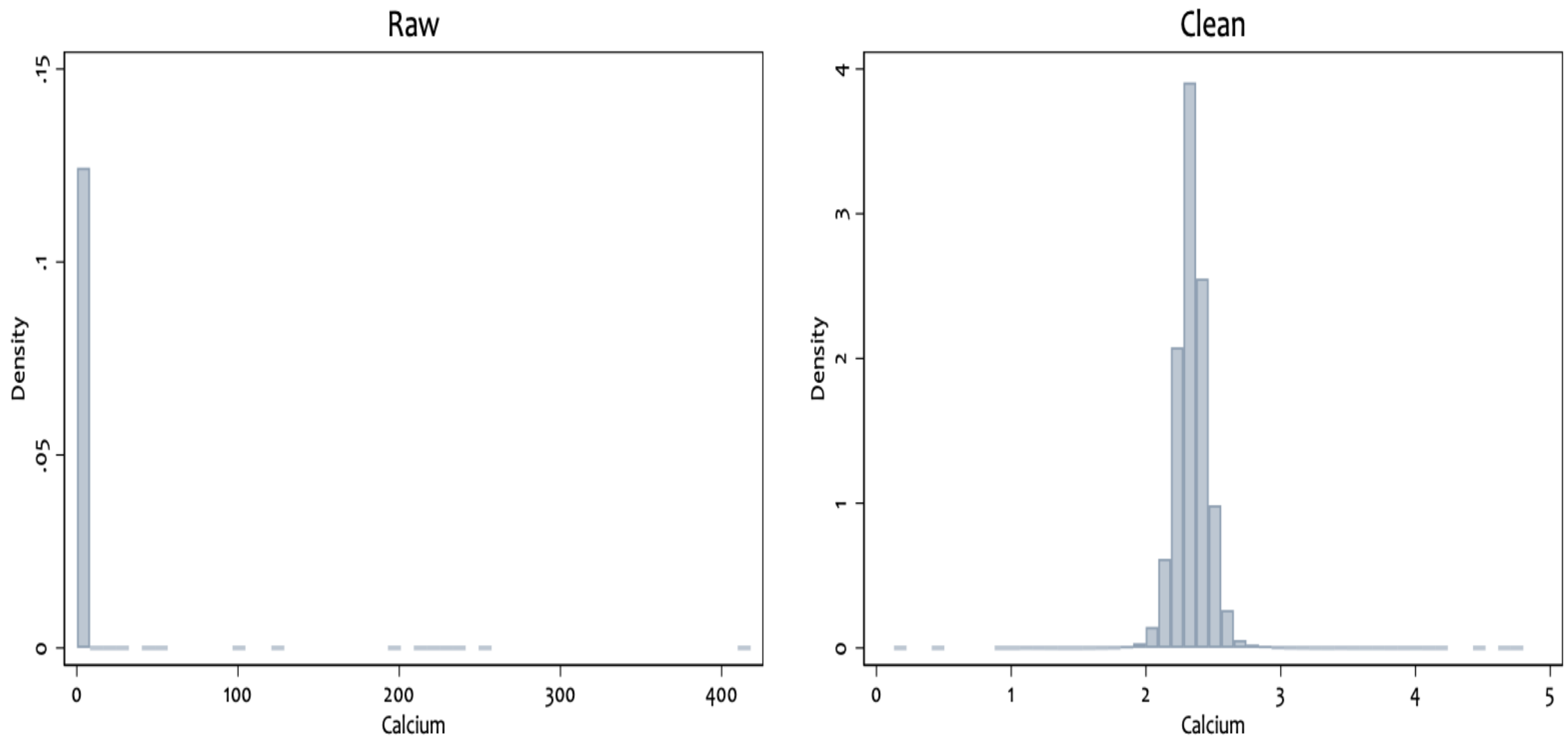

Calcium Adjusted

After cleaning, 107657 (13%) of patients have a measurement of calciumadjusted in the 1 year prior to index

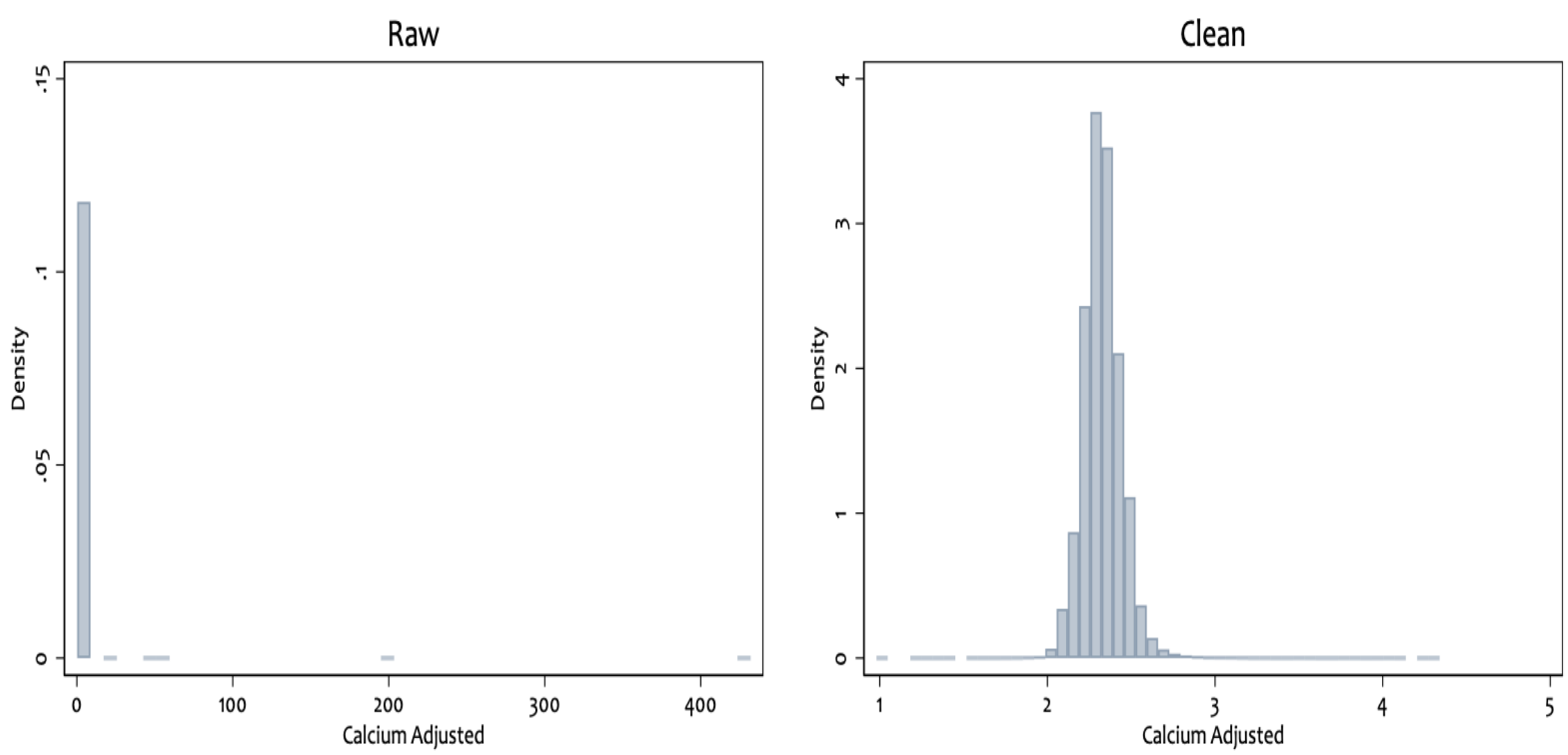

Basophil

After cleaning, 188444 (22%) of patients have a measurement of basophil in the 1 year prior to index

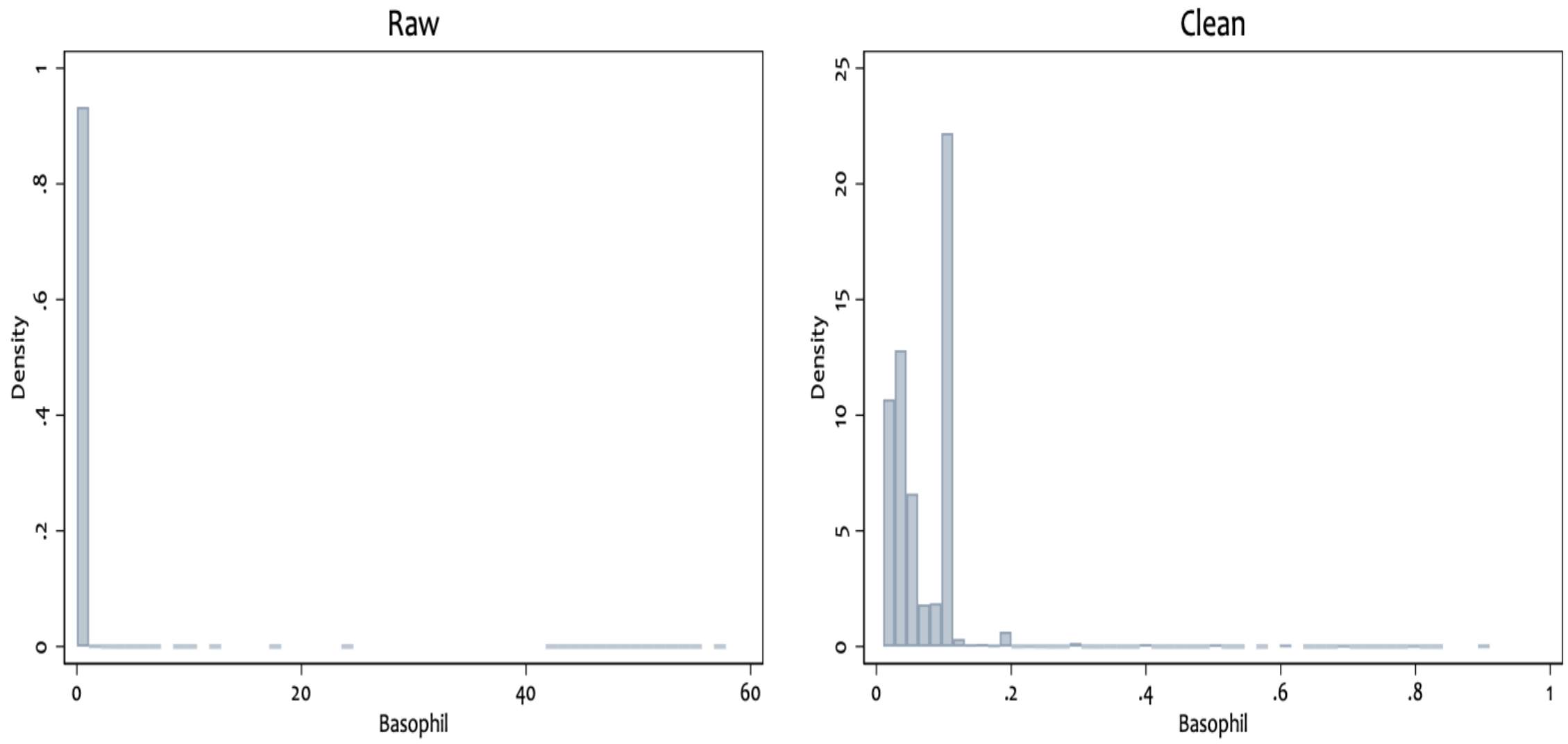

Eosinophil

After cleaning, 306517 (36%) of patients have a measurement of eosinophil in the 1 year prior to index

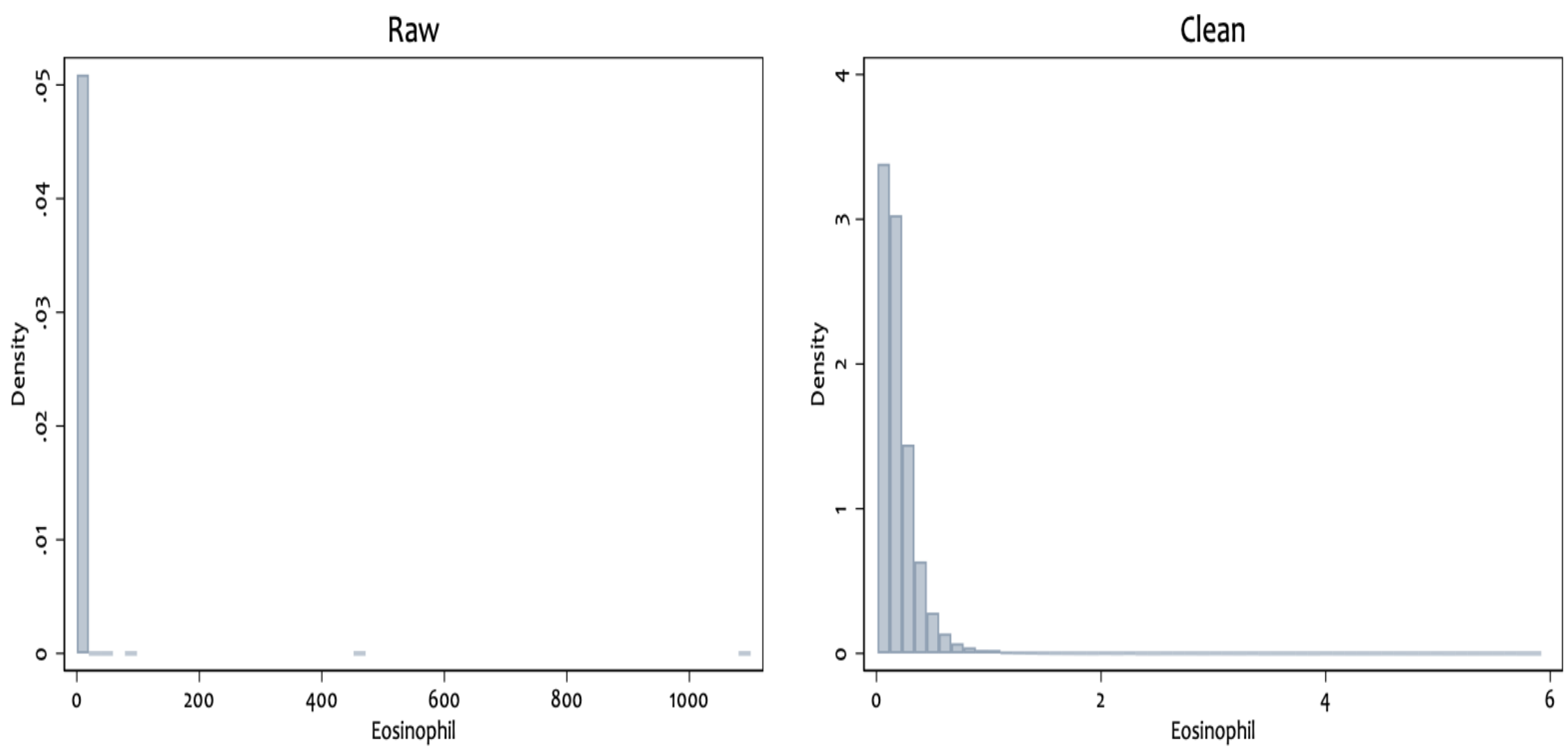

Haemoglobin

After cleaning, 343846 (40%) of patients have a measurement of haemoglobin in the 1 year prior to index

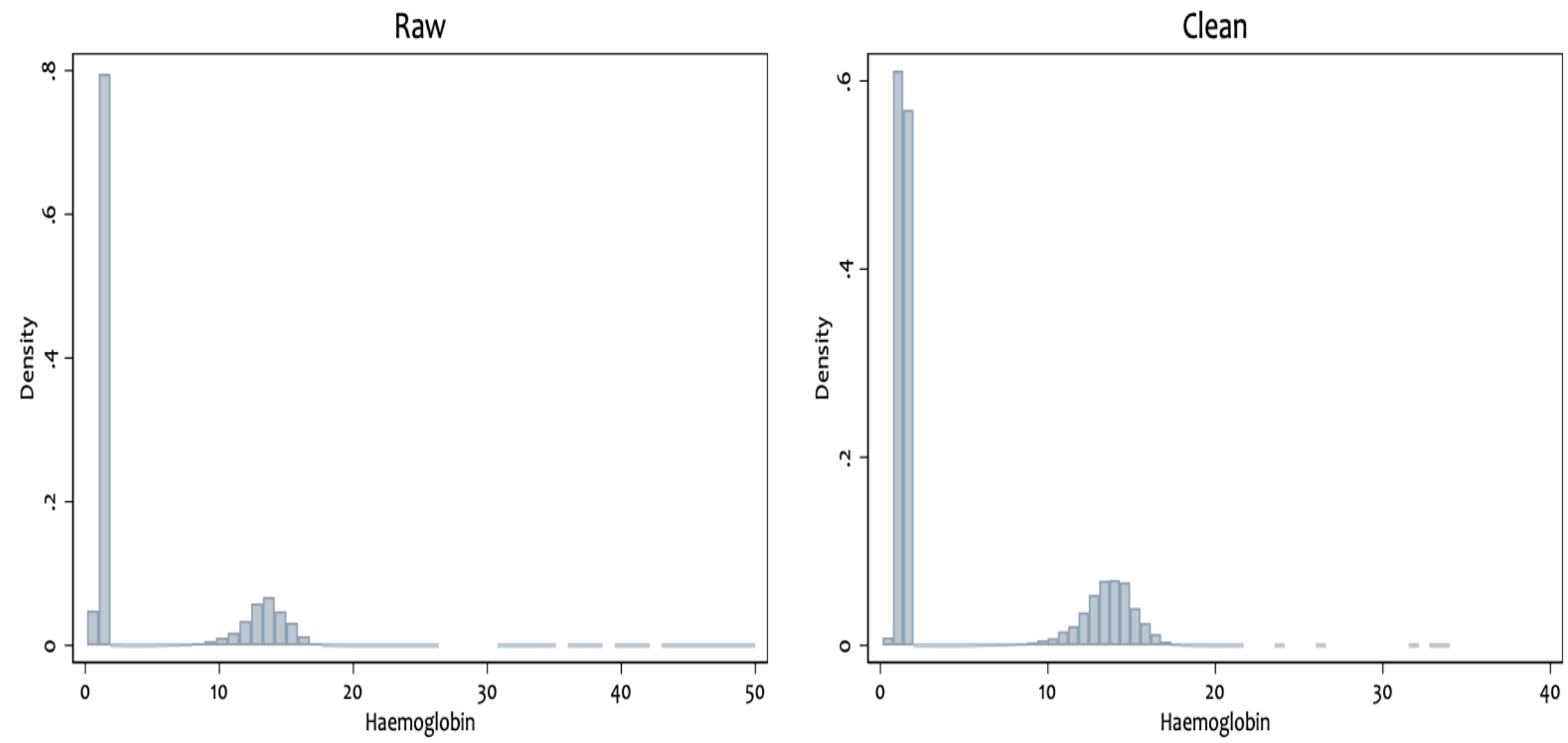

Lymphocyte

After cleaning, 318878 (37%) of patients have a measurement of lymphocyte in the 1 year prior to index

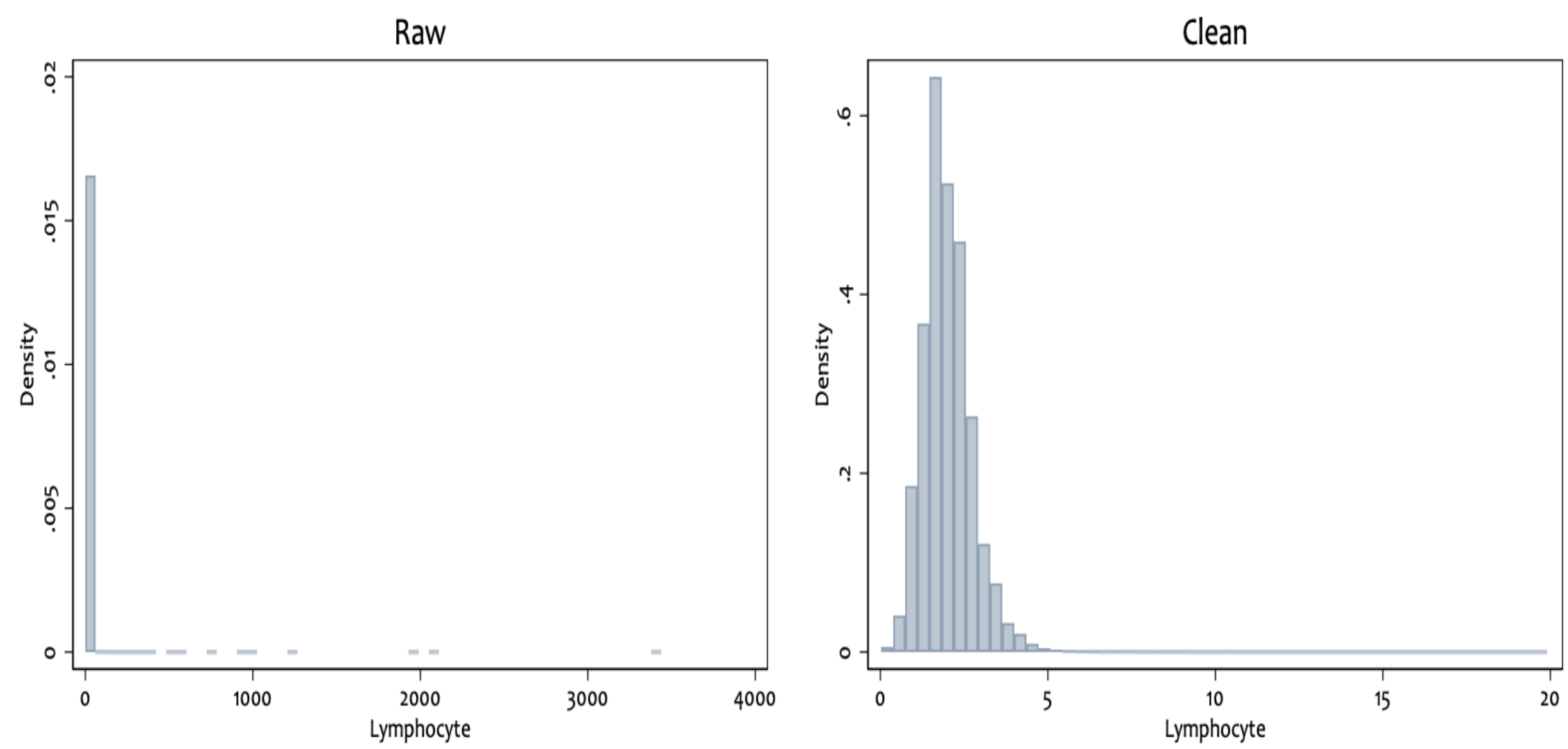

Monocytes

After cleaning, 315246 (37%) of patients have a measurement of monocytes in the 1 year prior to index

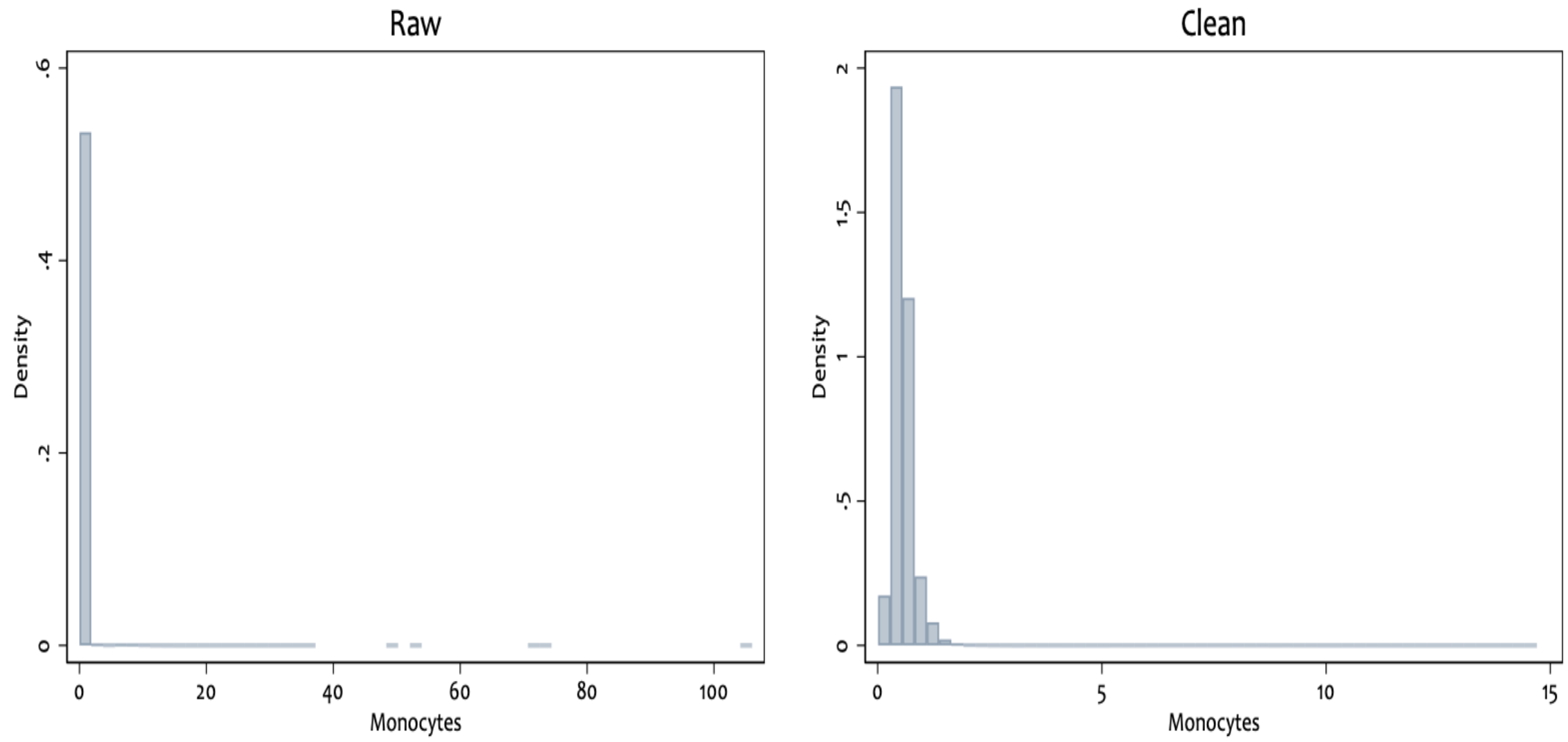

MCV

After cleaning, 334898 (39%) of patients have a measurement of mcv in the 1 year prior to index

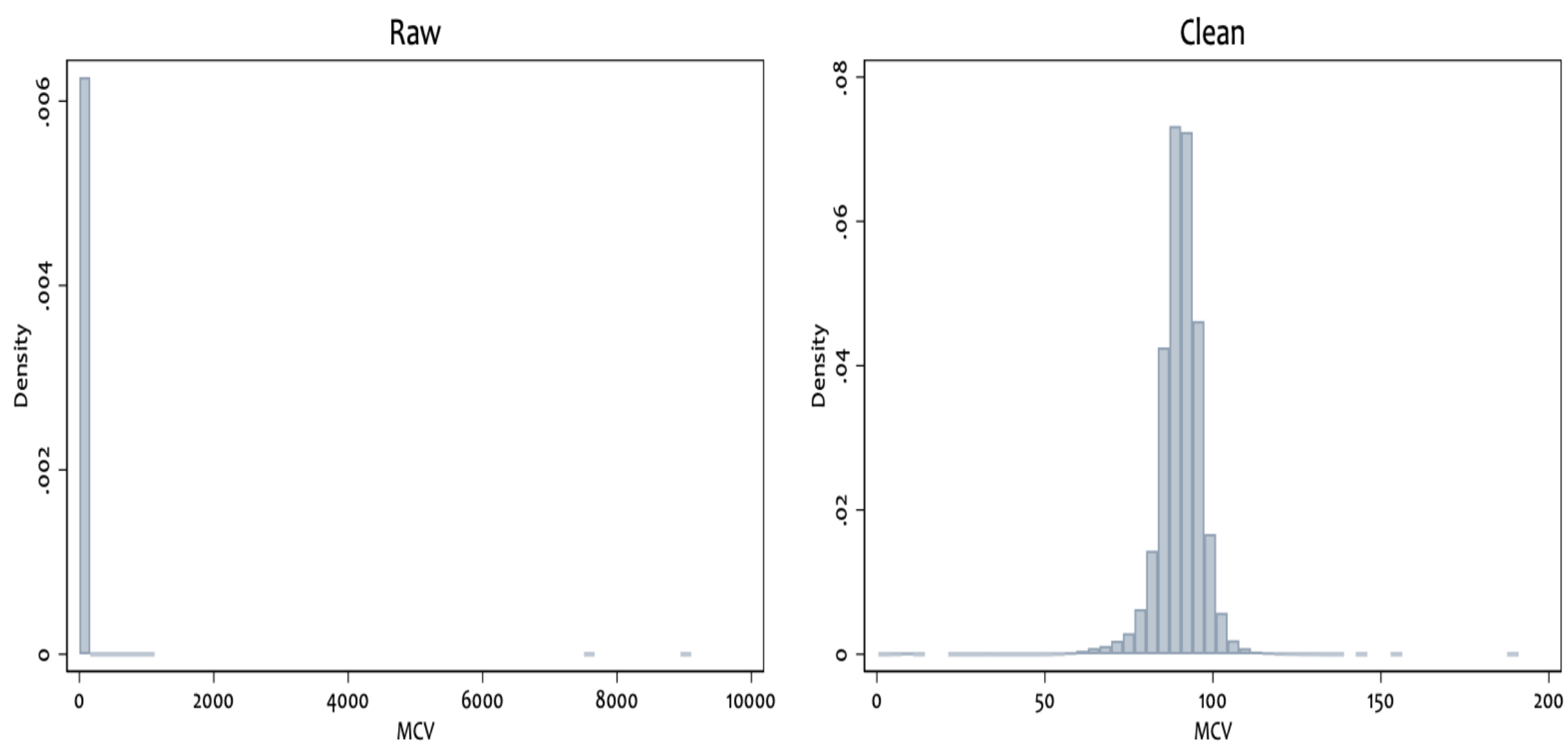

MCH

After cleaning, 311387 (36%) of patients have a measurement of mch in the 1 year prior to index

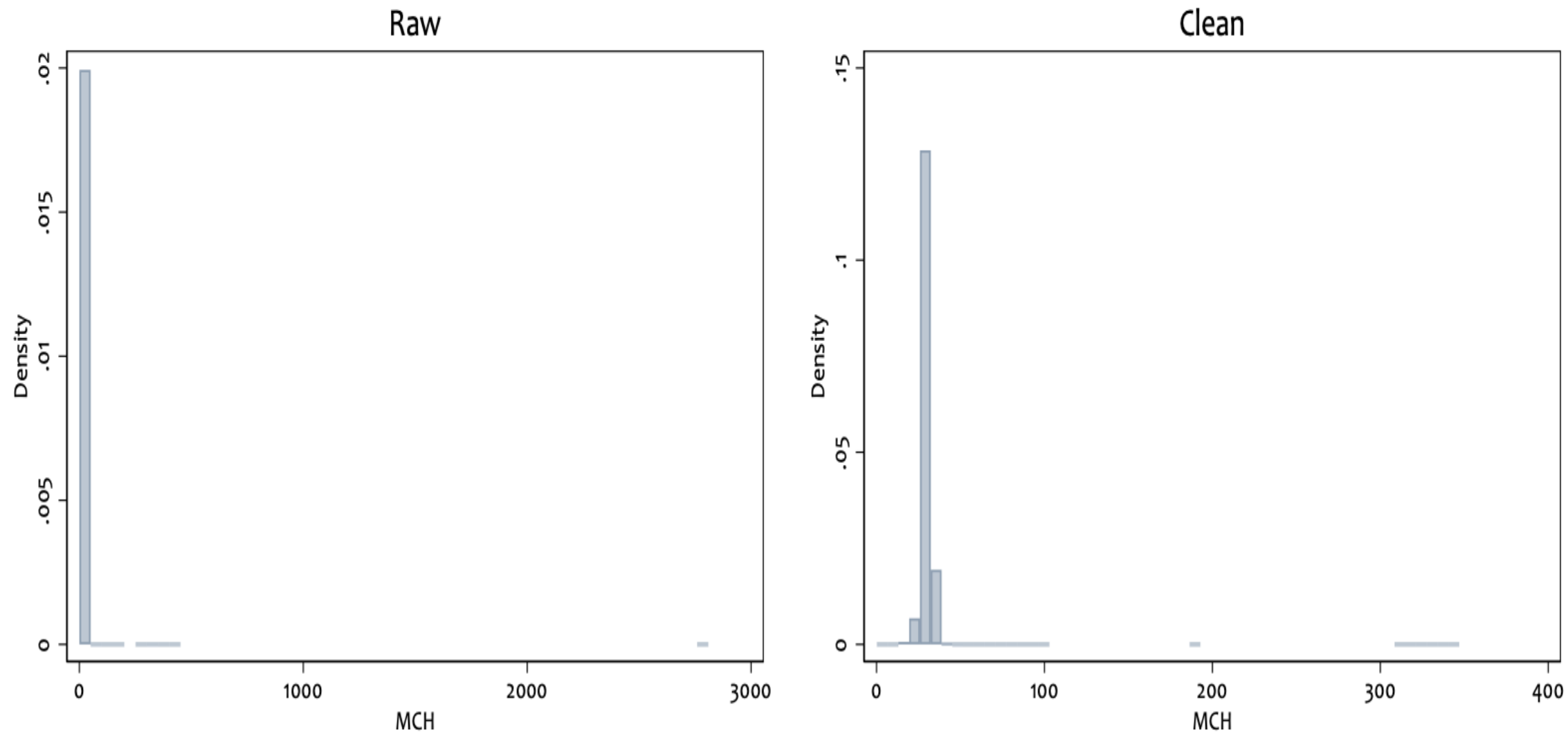

Platelets

After cleaning, 334552 (39%) of patients have a measurement of platelets in the 1 year prior to index

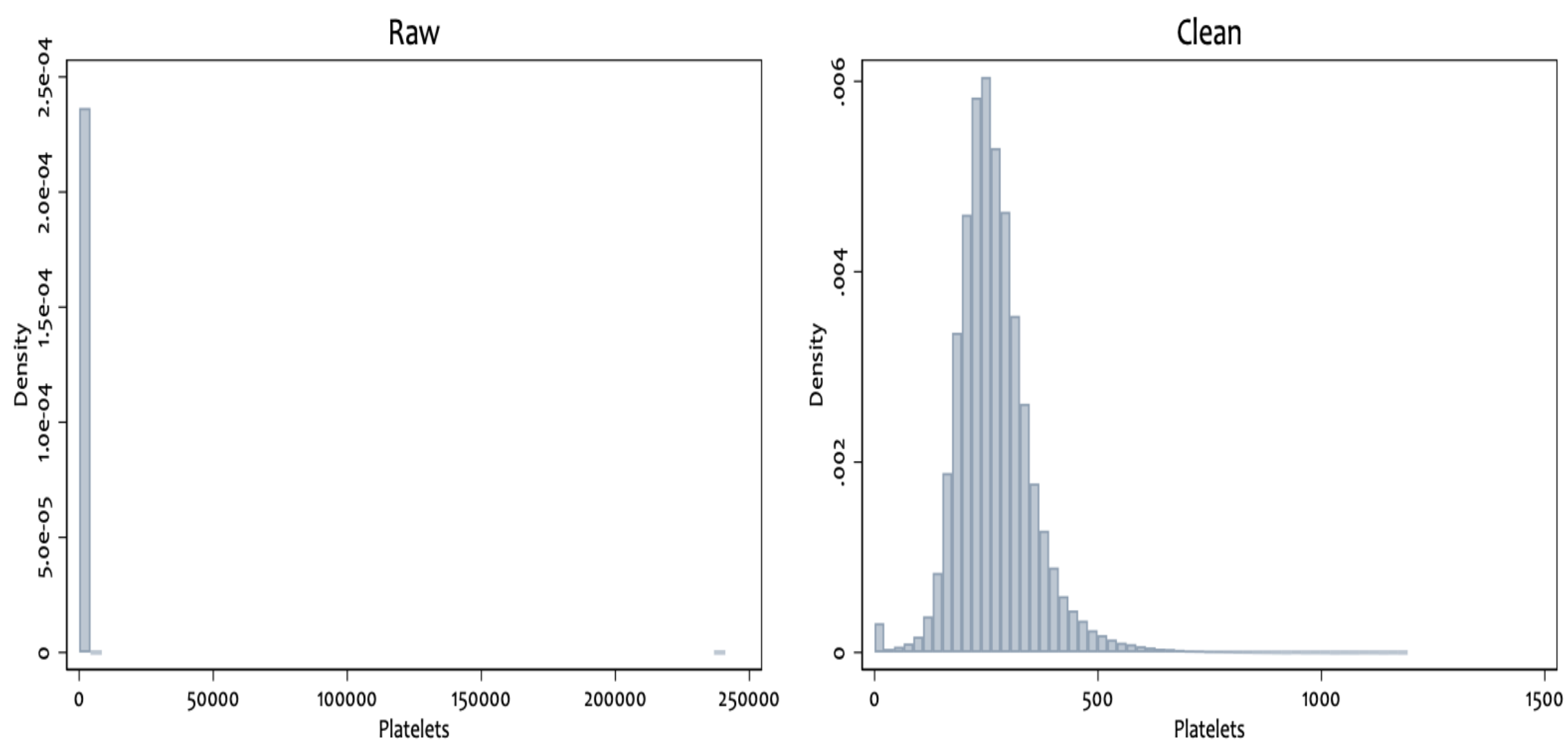

RBC

After cleaning, 7017 (1%) of patients have a measurement of rbc in the 1 year prior to index

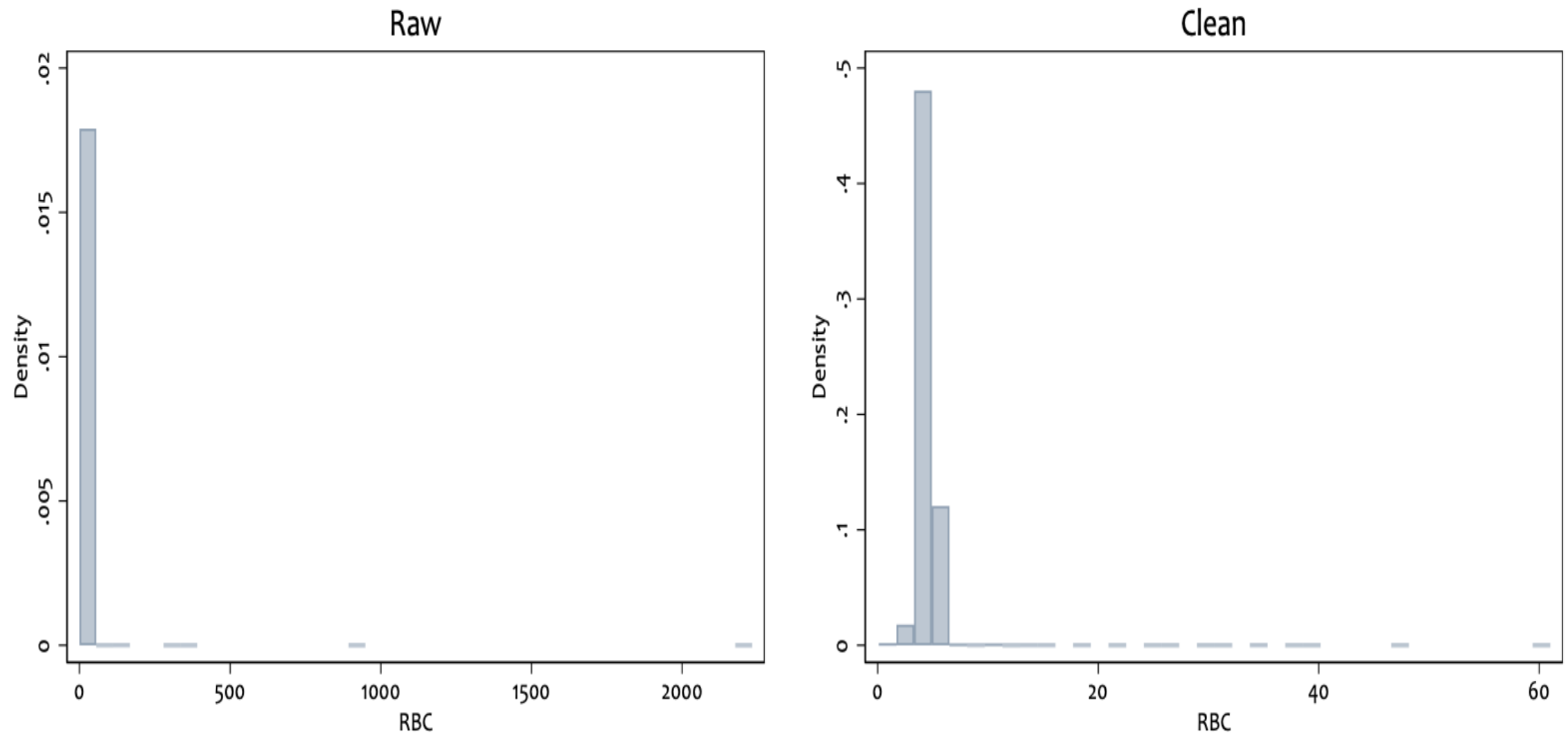

WBC

After cleaning, 330253 (38%) of patients have a measurement of wbc in the 1 year prior to index

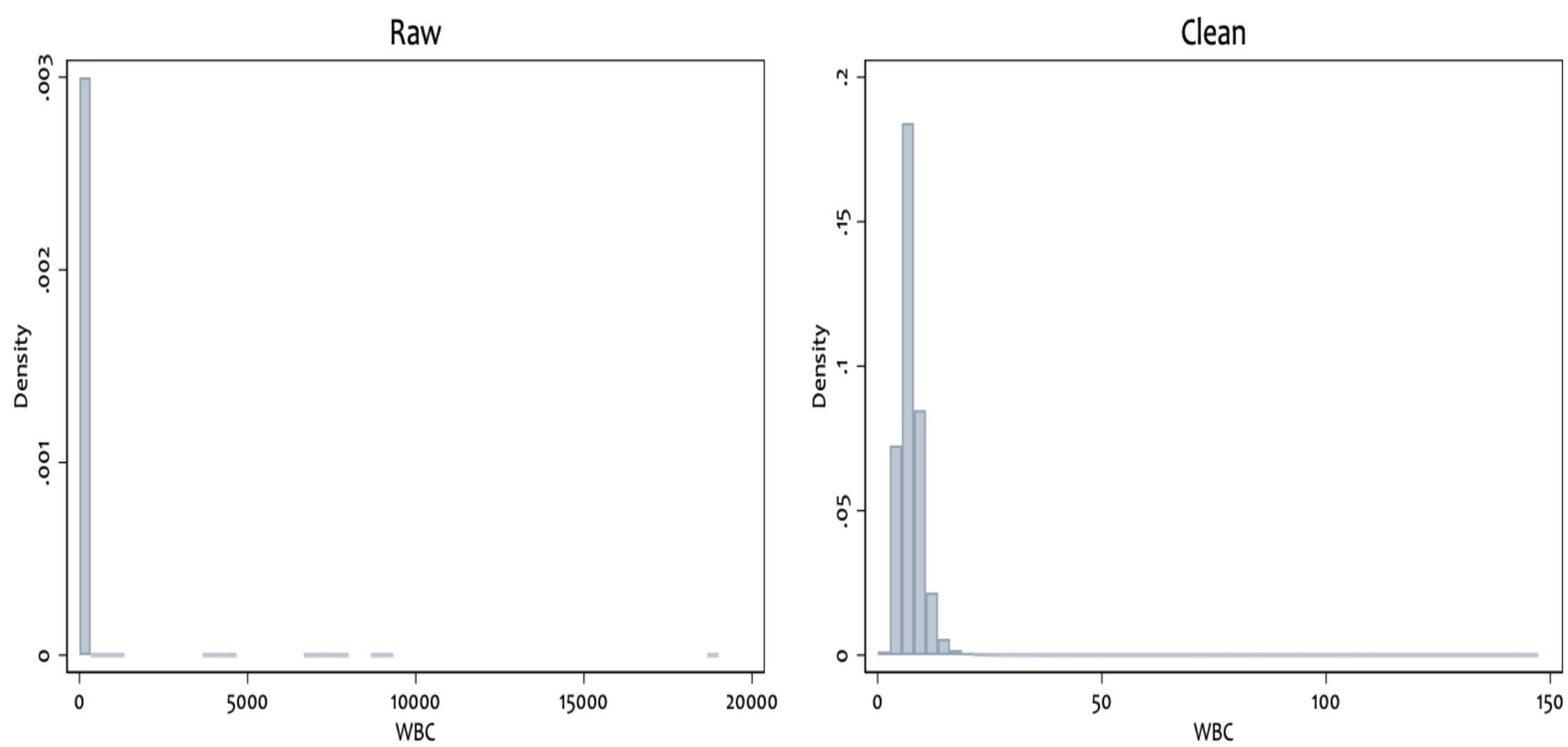

Glucose

After cleaning, 191916 (22%) of patients have a measurement of glucose in the 1 year prior to index

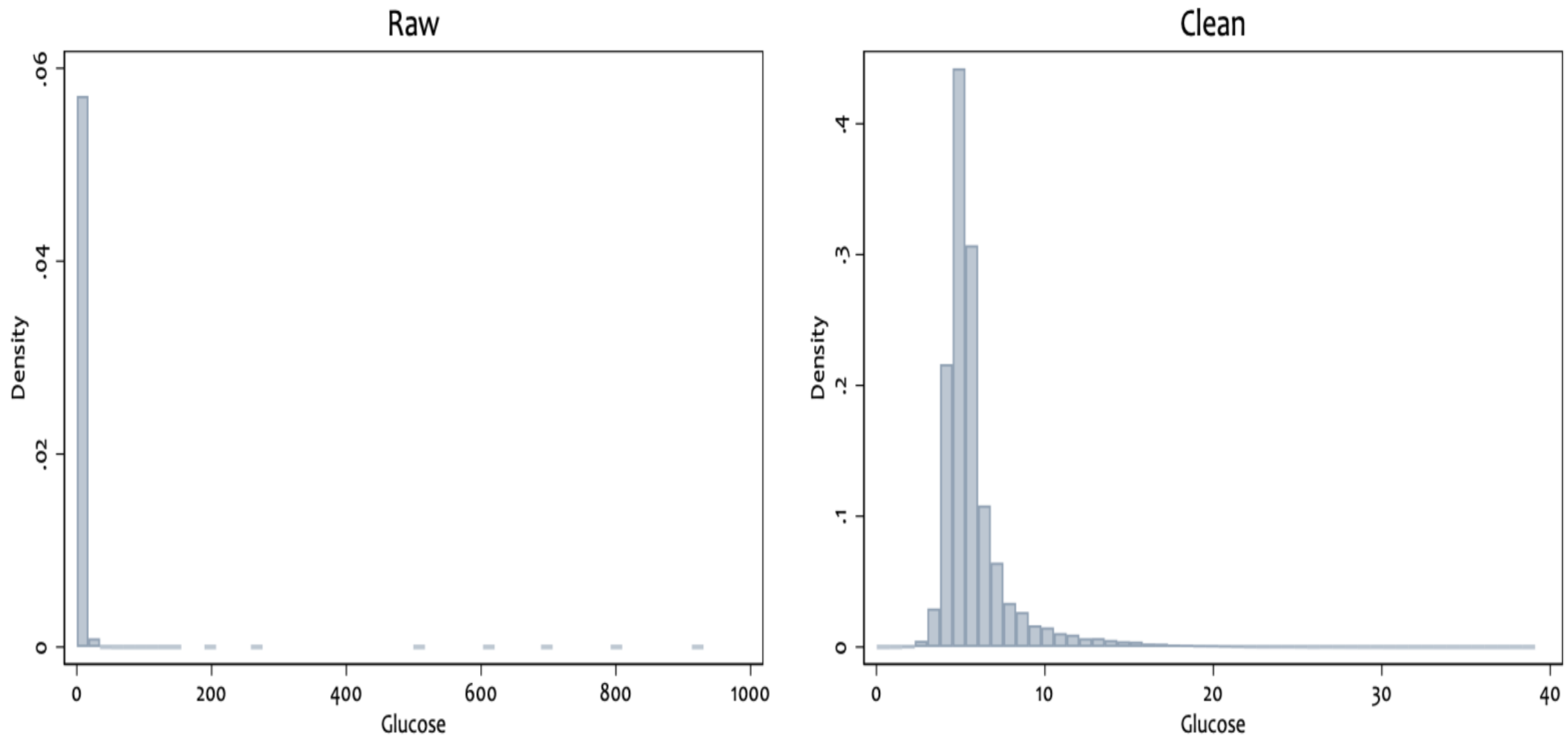

Glucosefasting

After cleaning, 71298 (8%) of patients have a measurement of glucosefasting in the 1 year prior to index

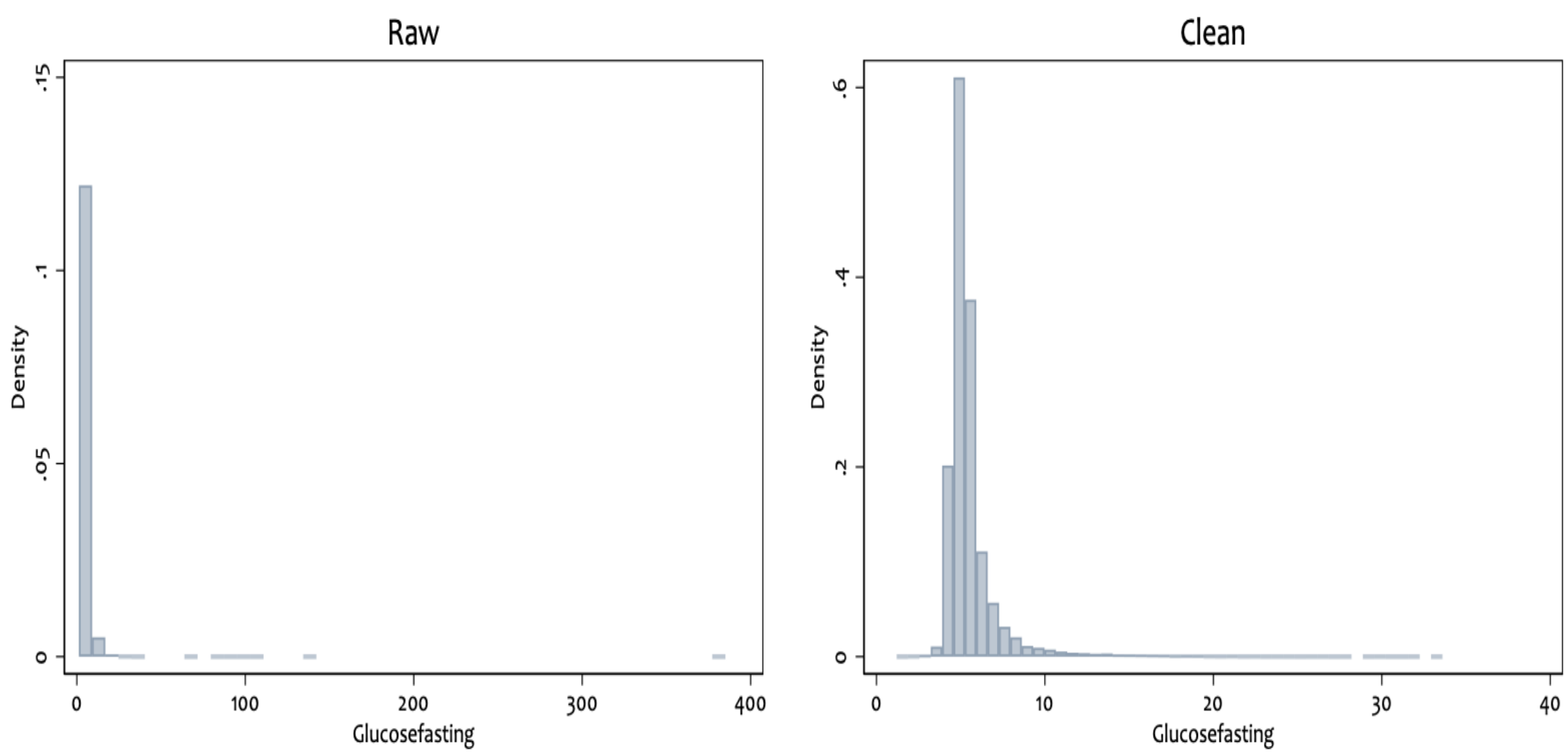

Cholesterol

After cleaning, 251515 (29%) of patients have a measurement of cholesterol in the 1 year prior to index

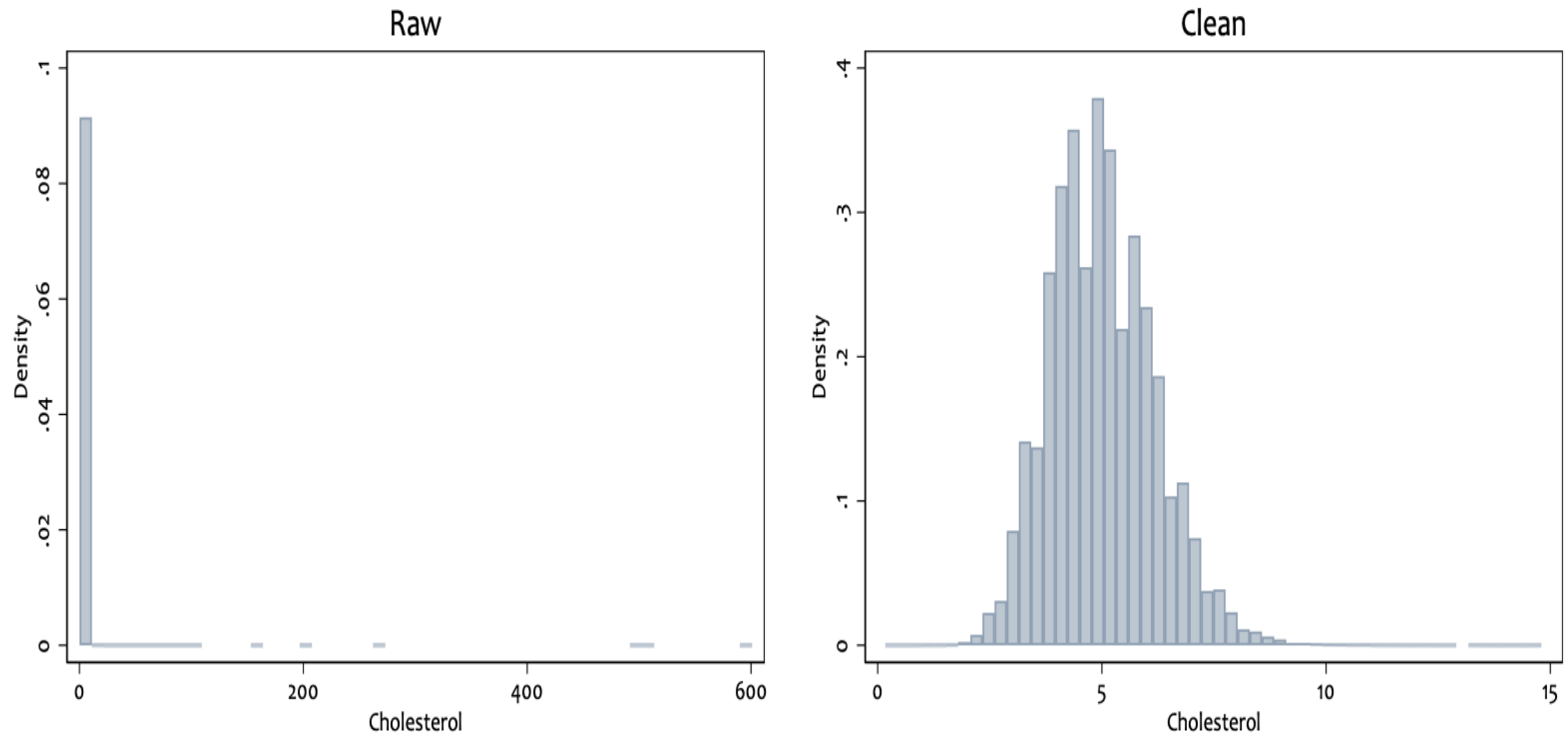

HDL

After cleaning, 201155 (23%) of patients have a measurement of hdl in the 1 year prior to index

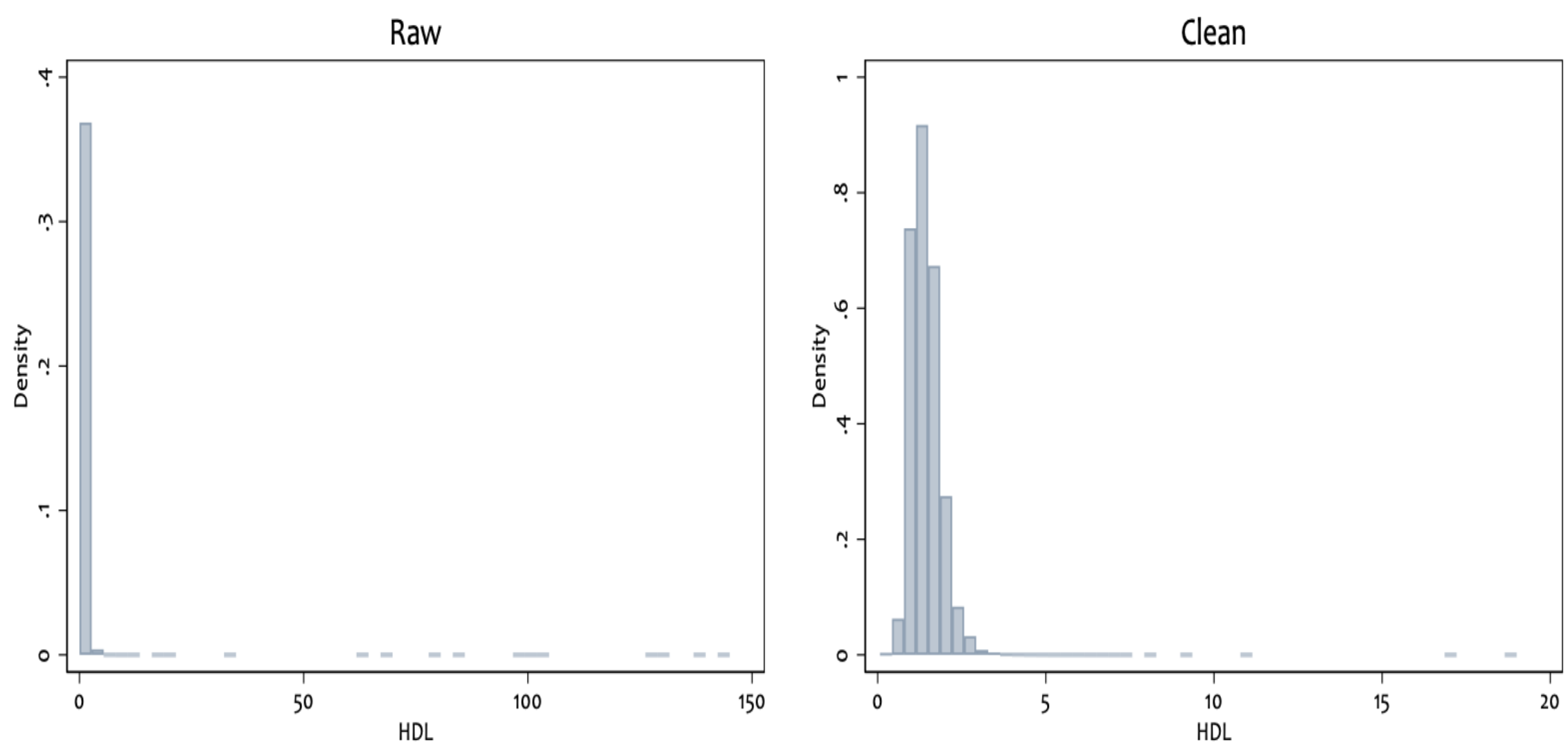

LDL

After cleaning, 161444 (19%) of patients have a measurement of ldl in the 1 year prior to index

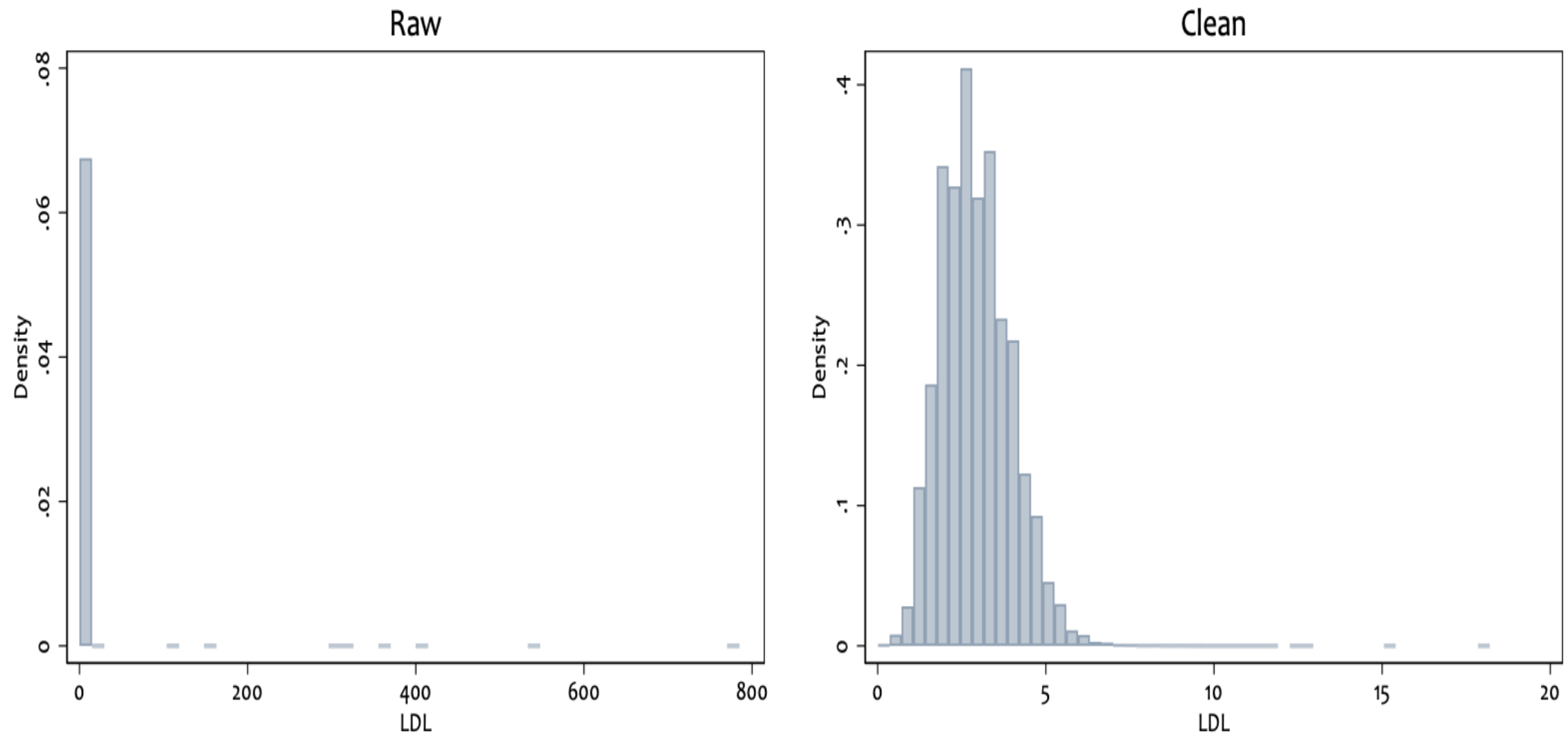

Triglycerides

After cleaning, 187138 (22%) of patients have a measurement of triglycerides in the 1 year prior to index

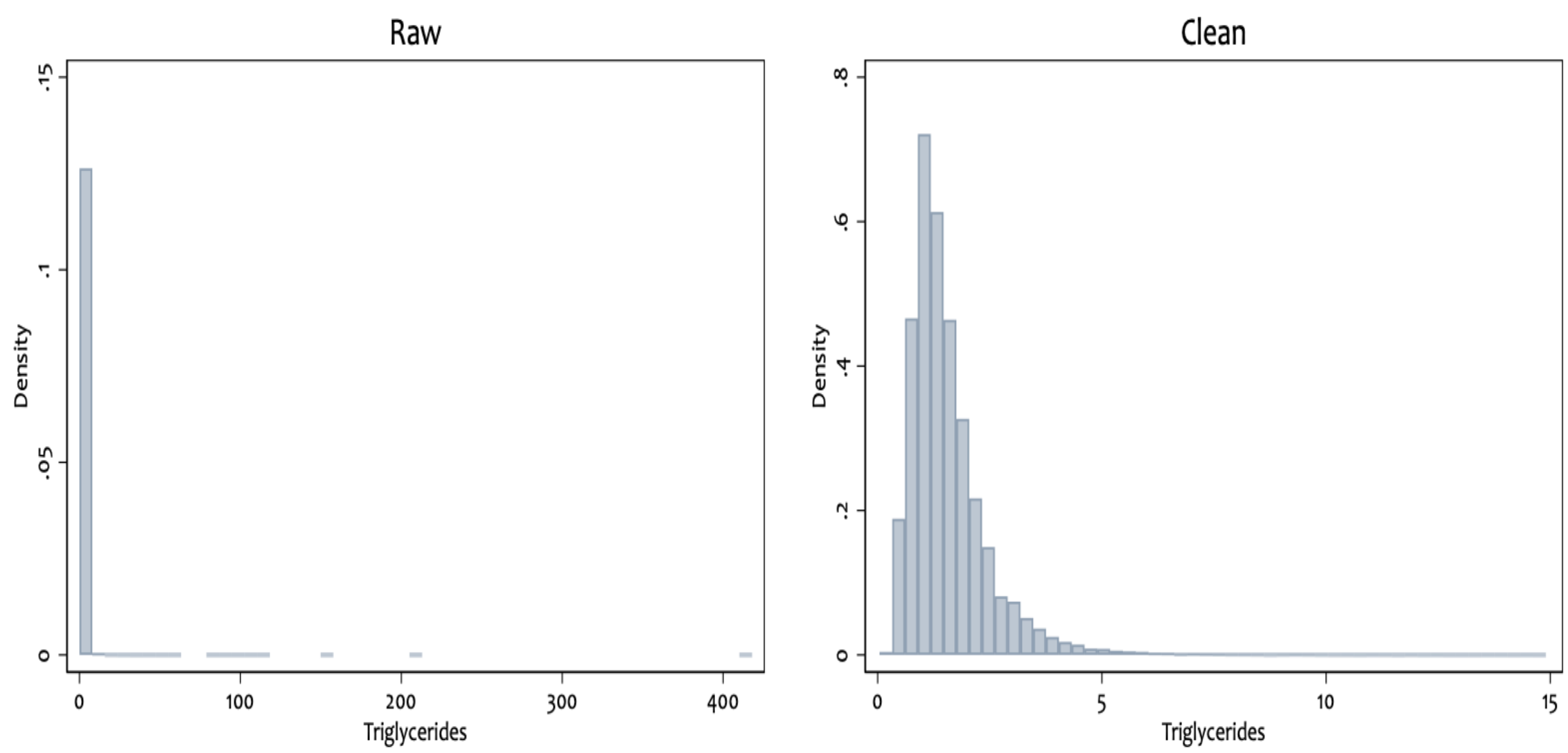

Albumin

After cleaning, 315127 (37%) of patients have a measurement of albumin in the 1 year prior to index

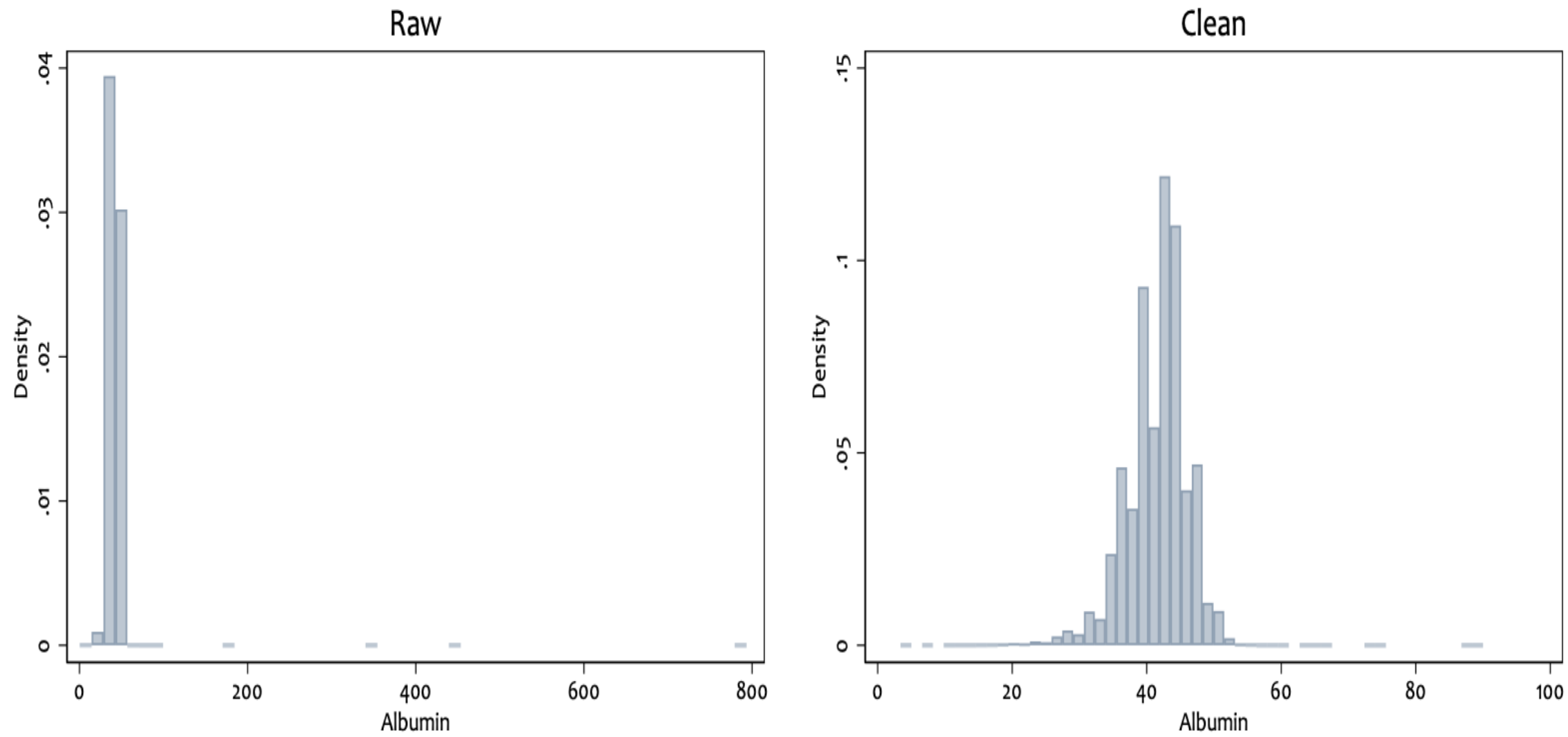

AST

After cleaning, 68153 (8%) of patients have a measurement of ast in the 1 year prior to index

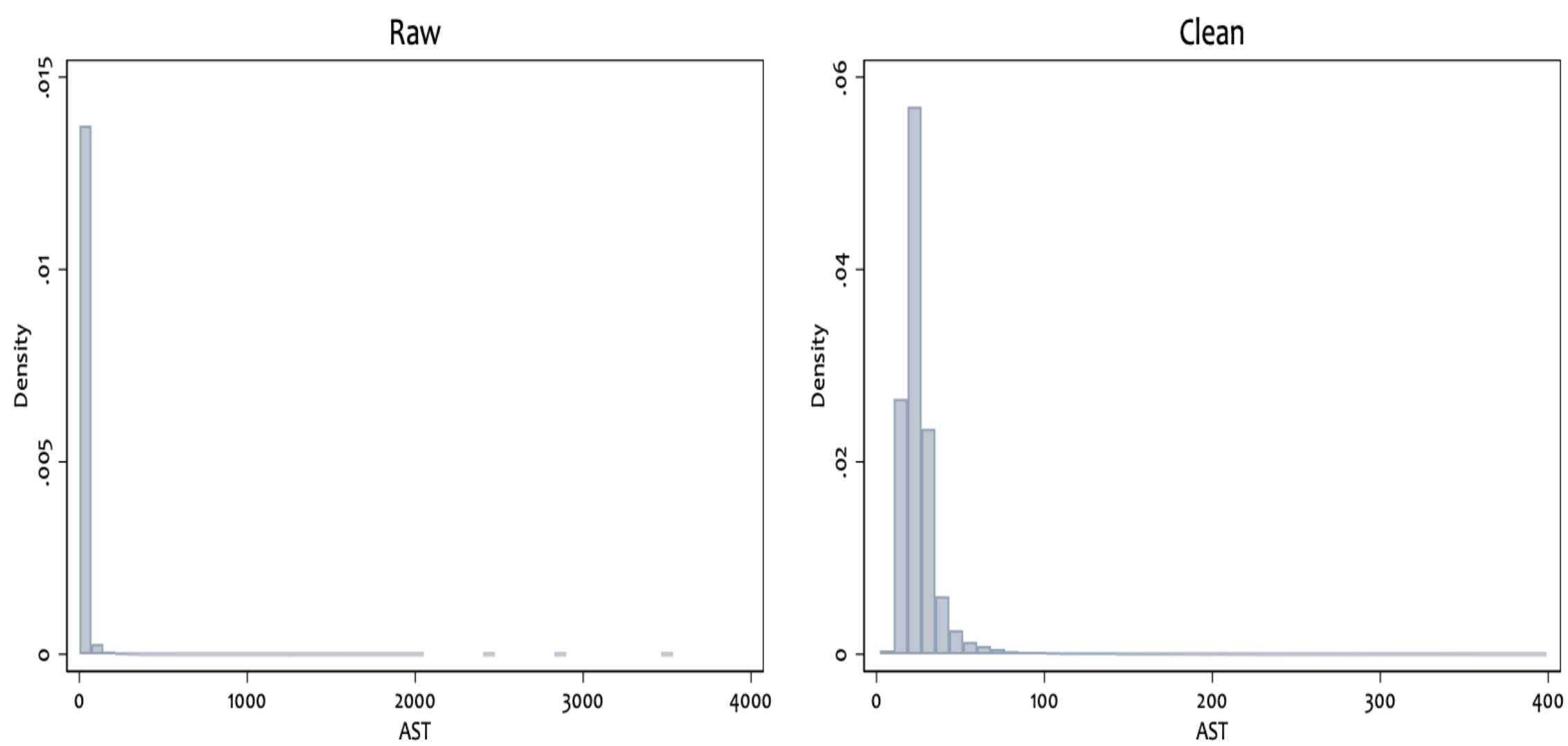

ALT

After cleaning, 262611 (31%) of patients have a measurement of alt in the 1 year prior to index

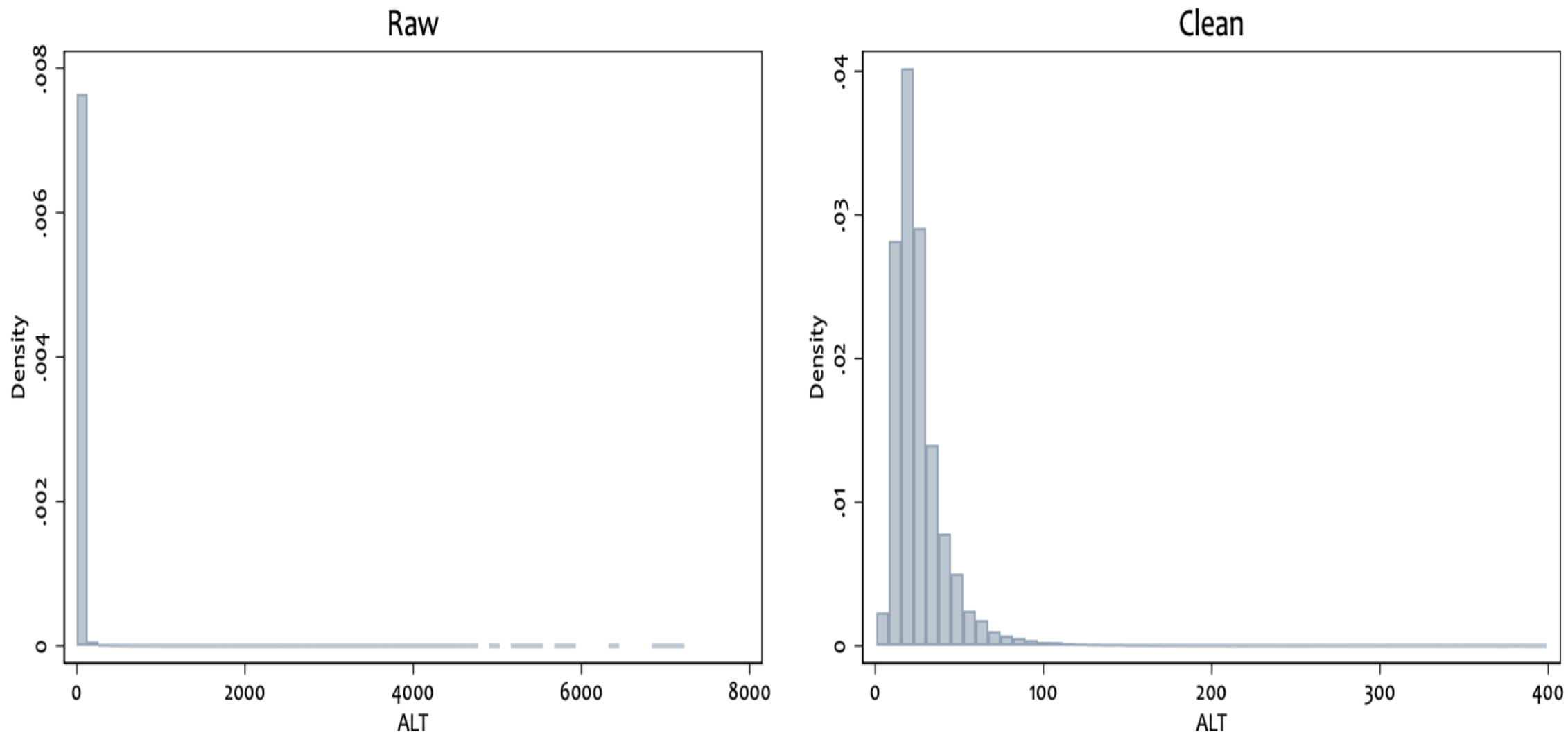

ALP

After cleaning, 265843 (31%) of patients have a measurement of alp in the 1 year prior to index

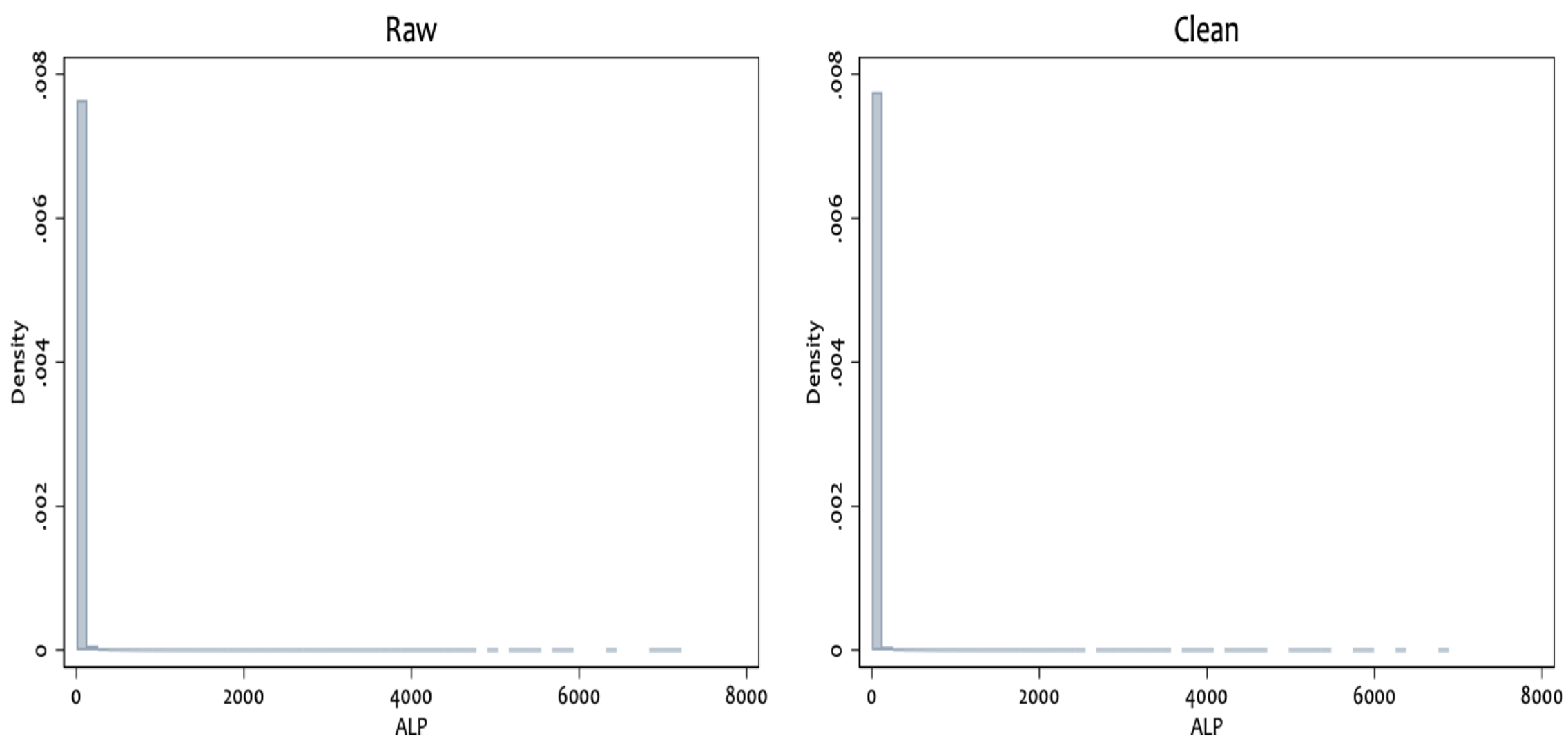

AKP

After cleaning, 313093 (36%) of patients have a measurement of akp in the 1 year prior to index

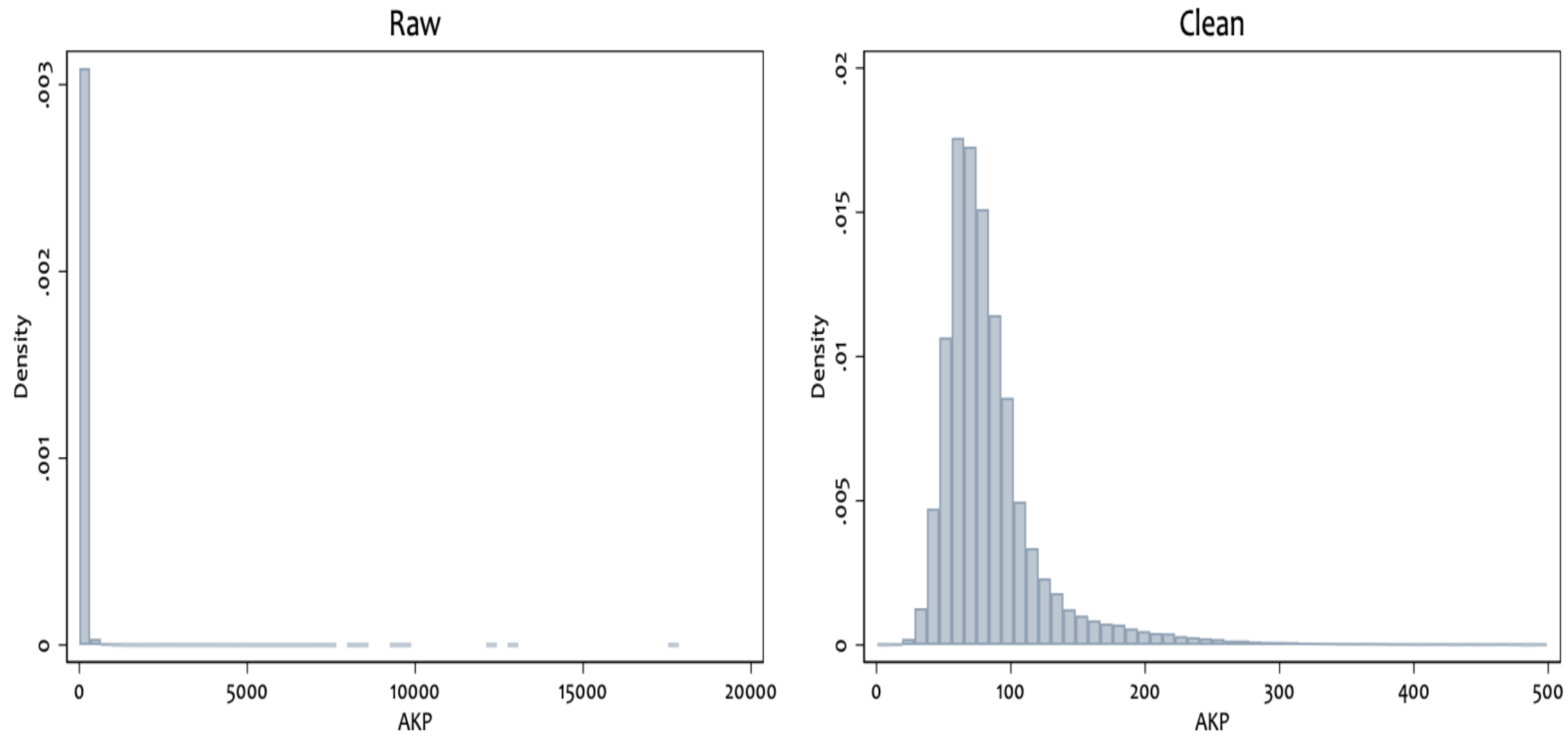

<u>Bilirubin</u>

After cleaning, 313489 (37%) of patients have a measurement of bilirubin in the 1 year prior to index

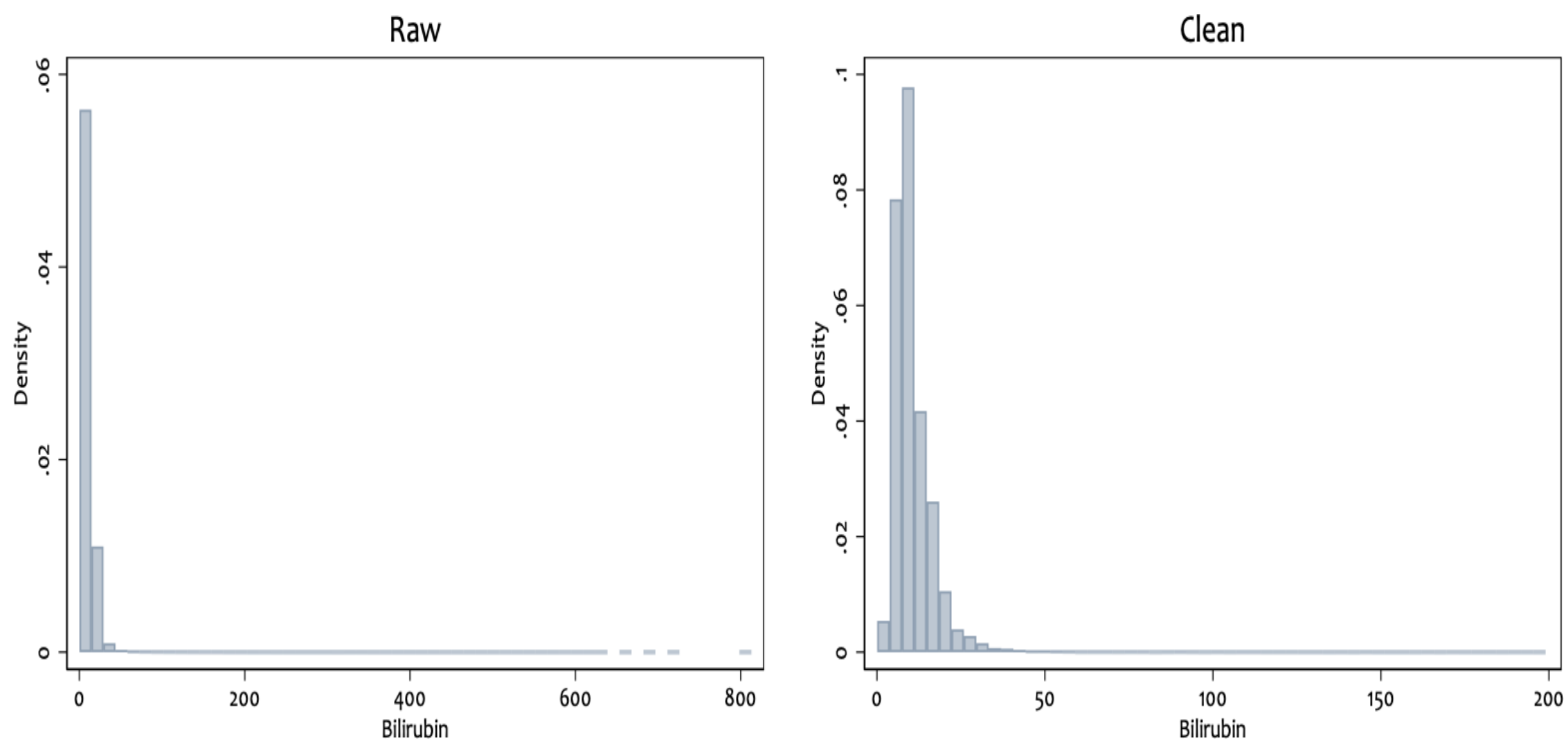

<u>Creatinine</u>

After cleaning, 365707 (43%) of patients have a measurement of creatinine in the 1 year prior to index

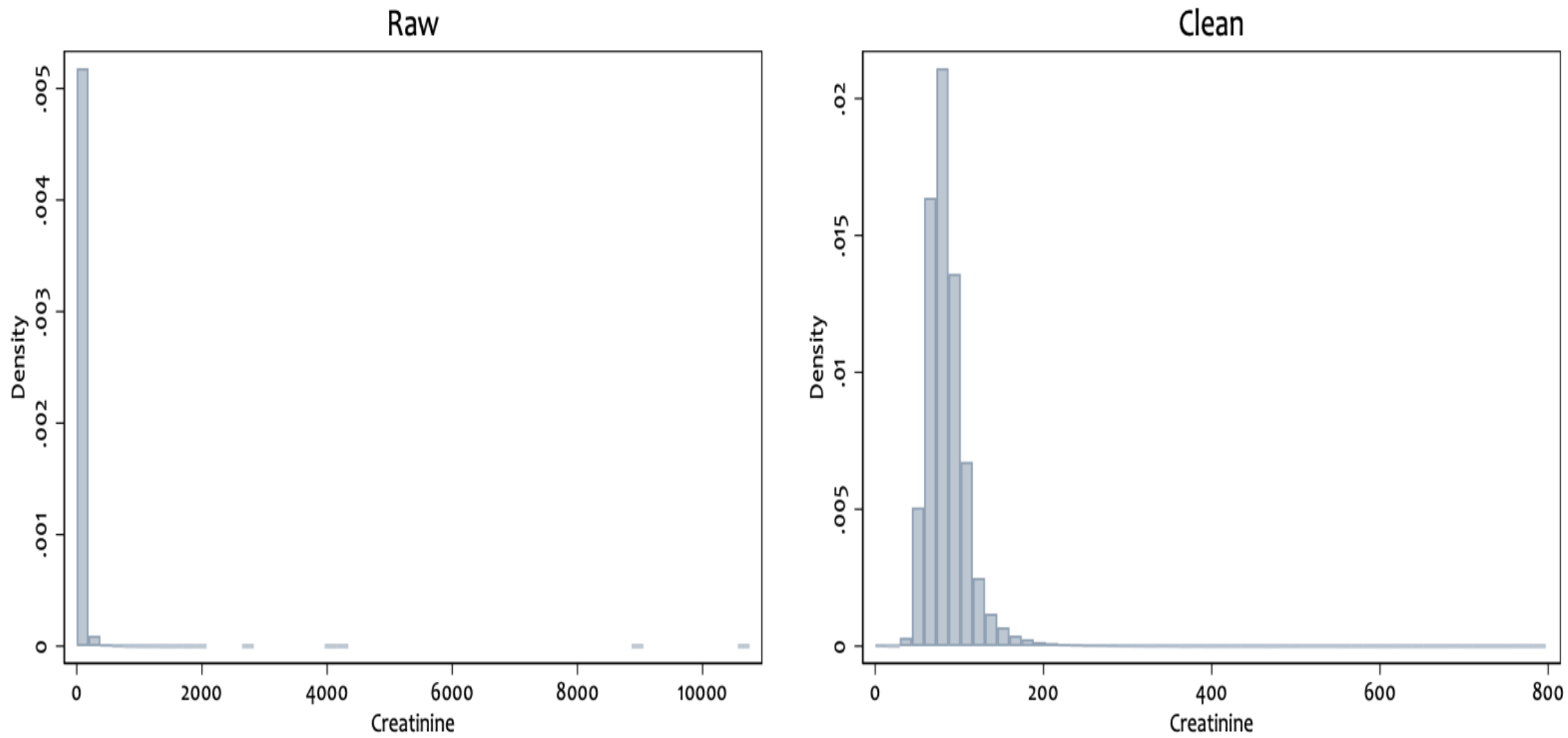

Potassium

After cleaning, 357462 (42%) of patients have a measurement of potassium in the 1 year prior to index

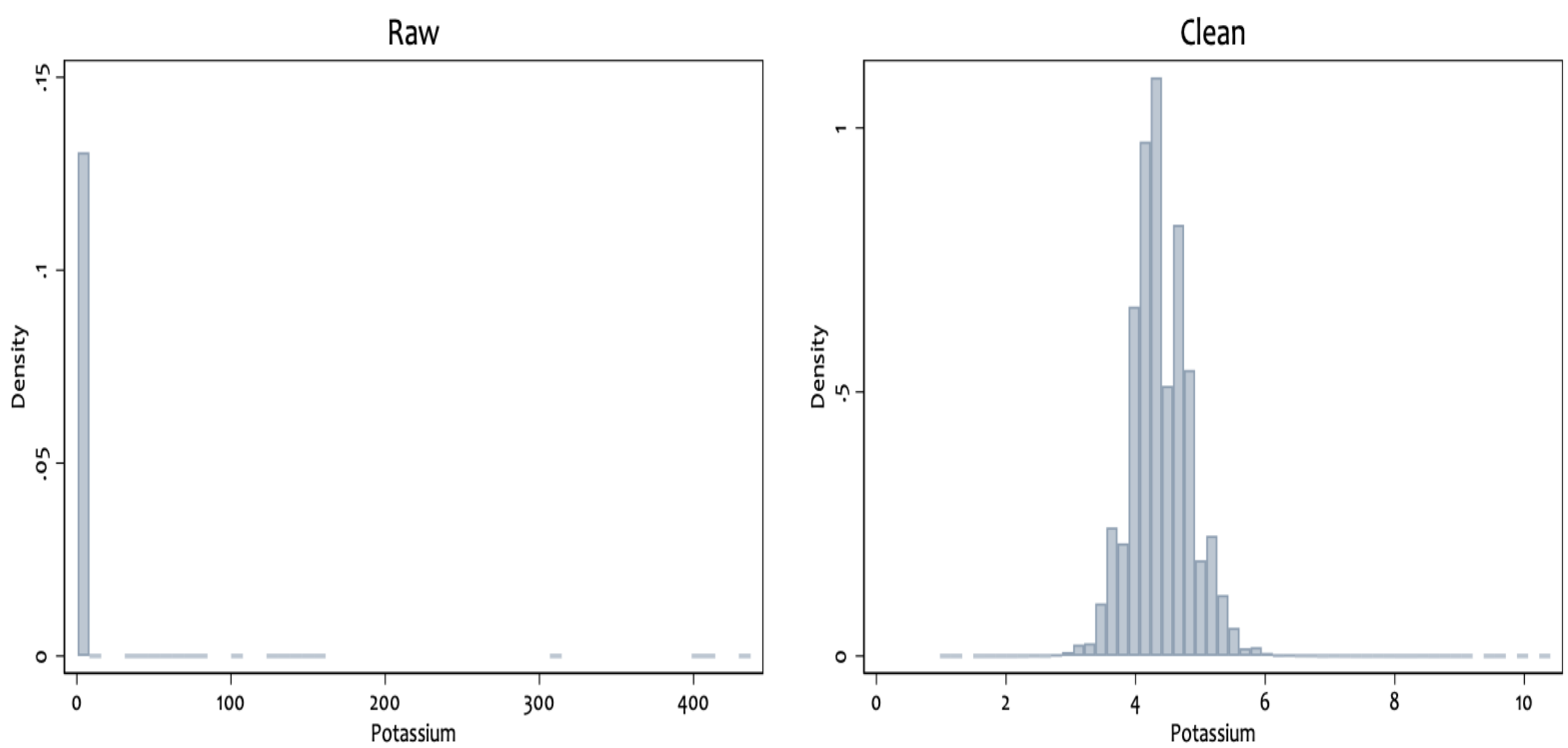

Sodium

After cleaning, 359888 (42%) of patients have a measurement of sodium in the 1 year prior to index

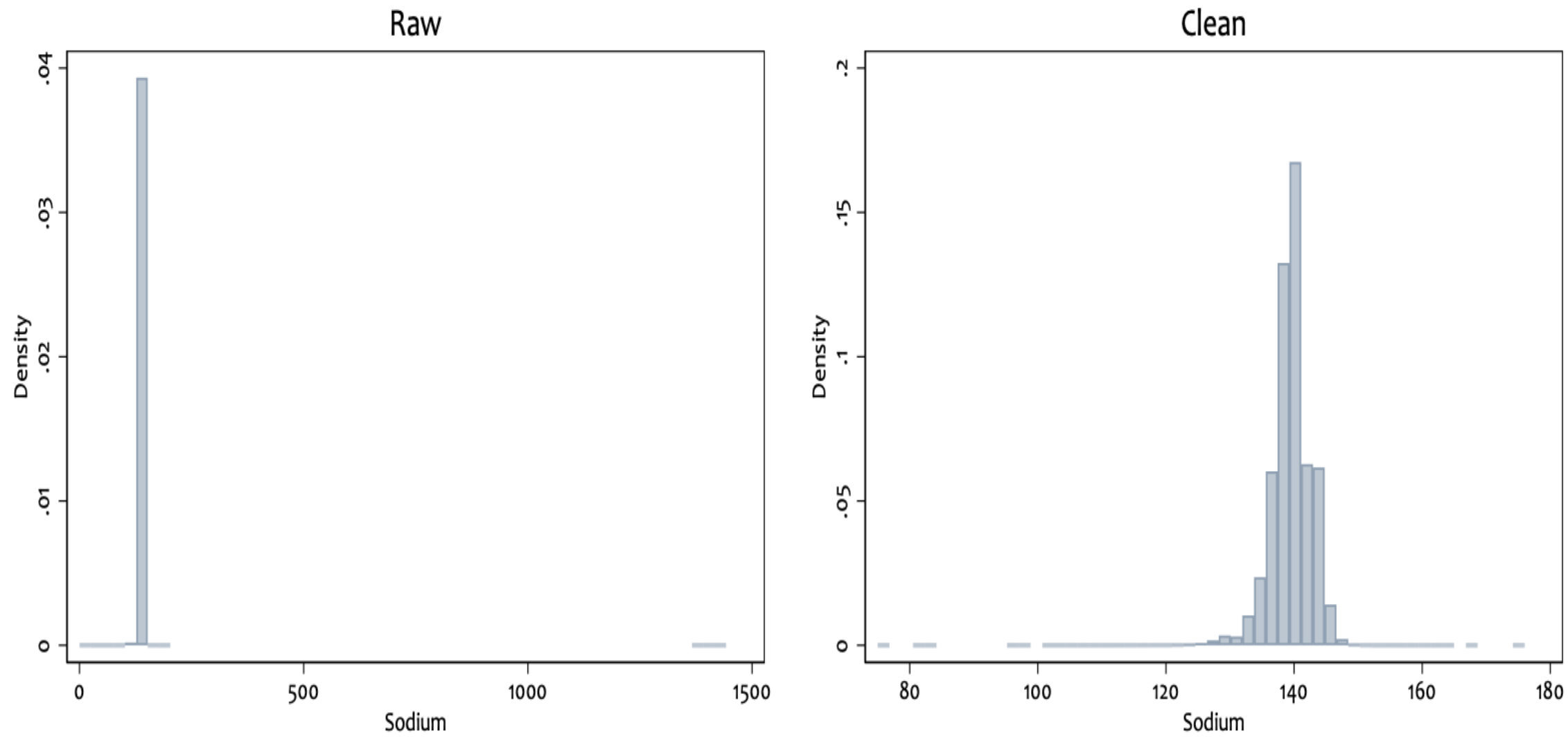

Urea

After cleaning, 289881 (34%) of patients have a measurement of urea in the 1 year prior to index

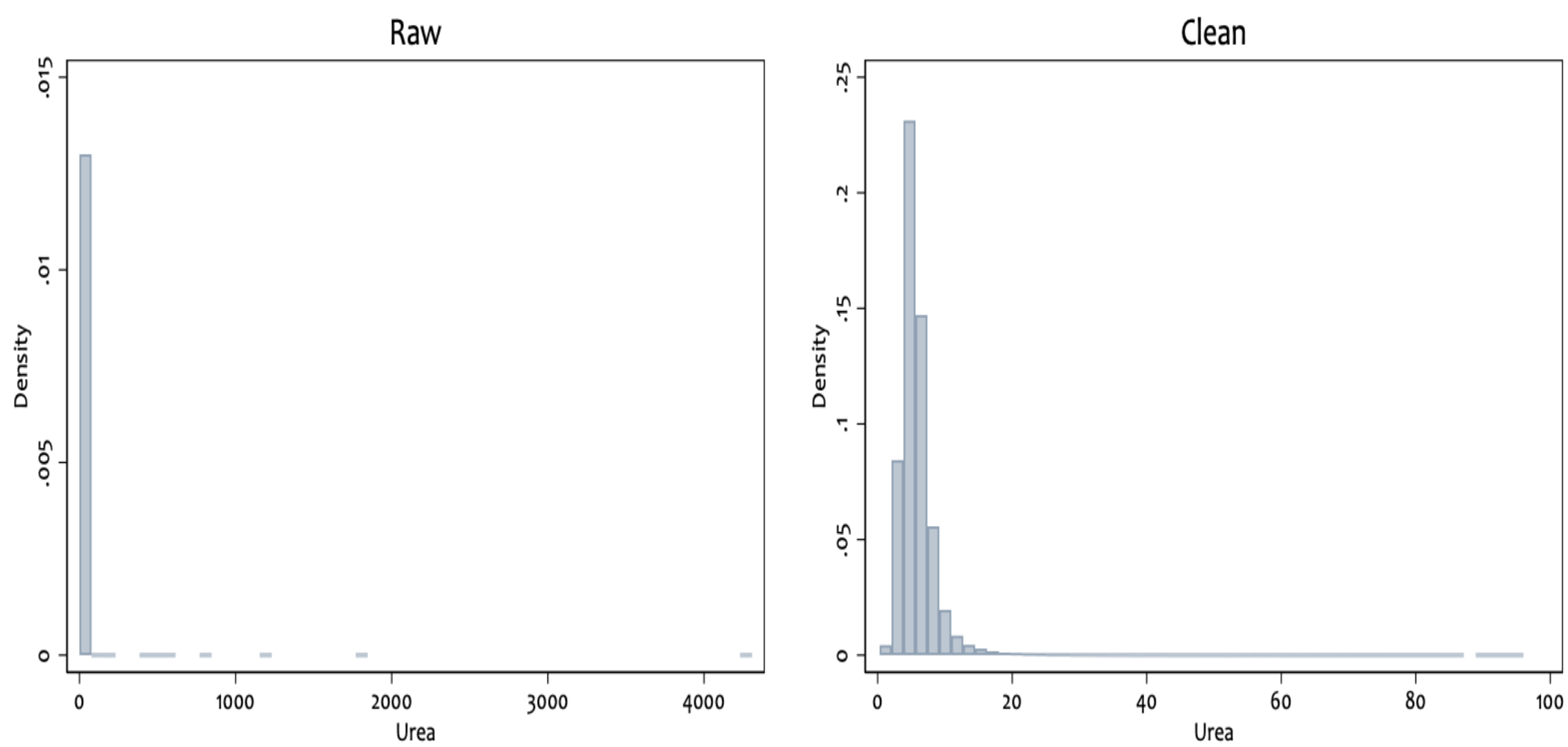

GFR

After cleaning, 159477 (19%) of patients have a measurement of gfr in the 1 year prior to index

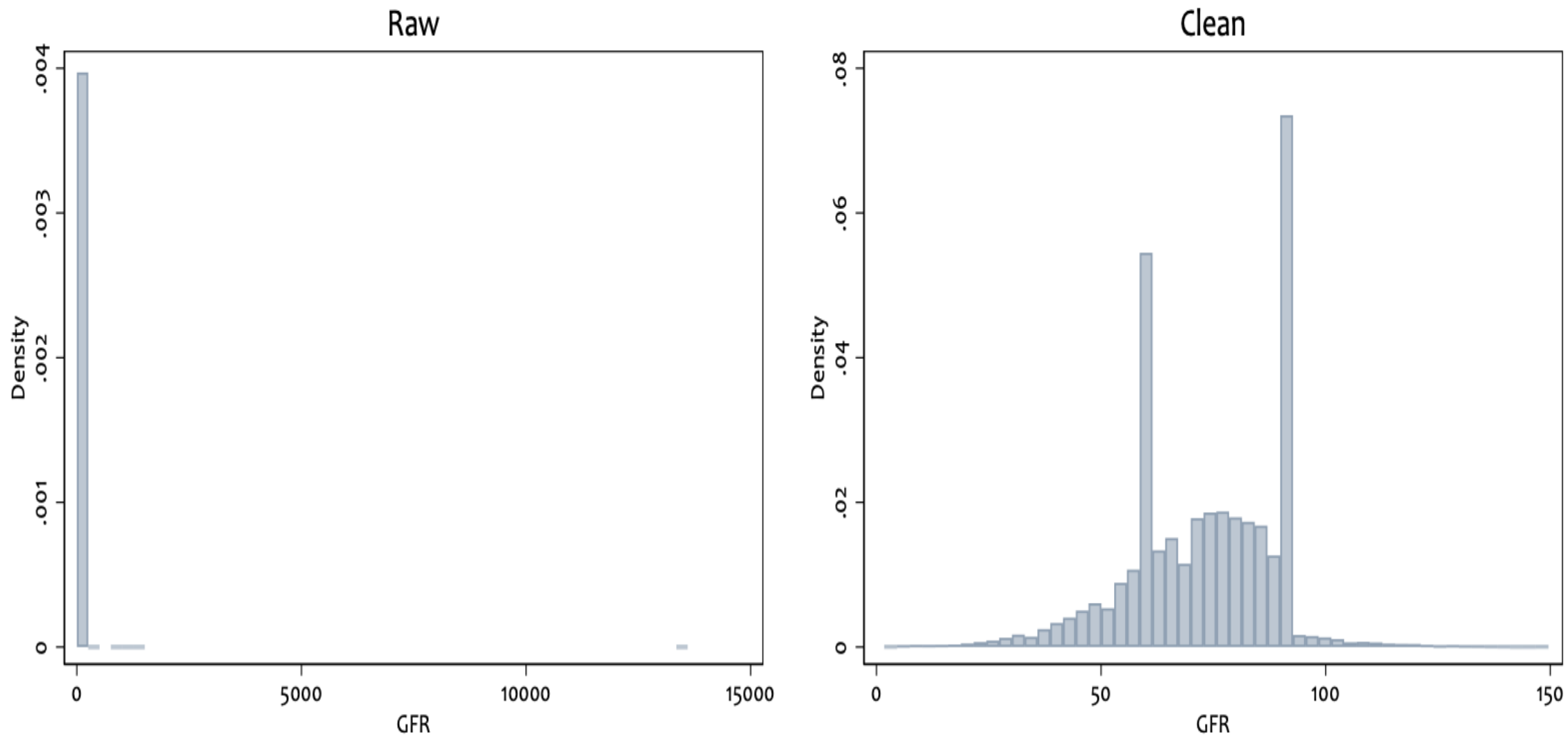

CRP

After cleaning, 94259 (11%) of patients have a measurement of crp in the 1 year prior to index

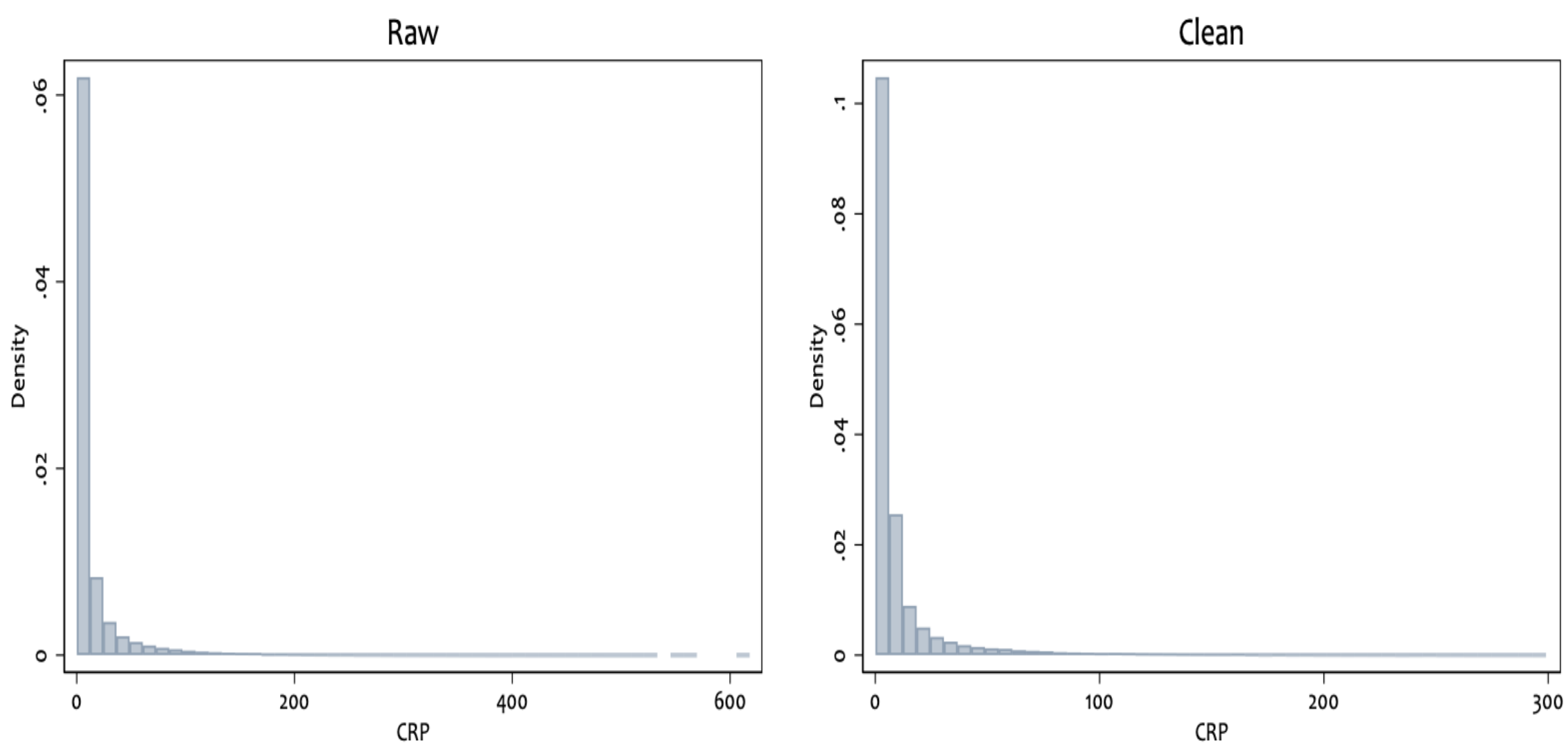

HbA1c

After cleaning, 72543 (8%) of patients have a measurement of hba1c in the 1 year prior to index

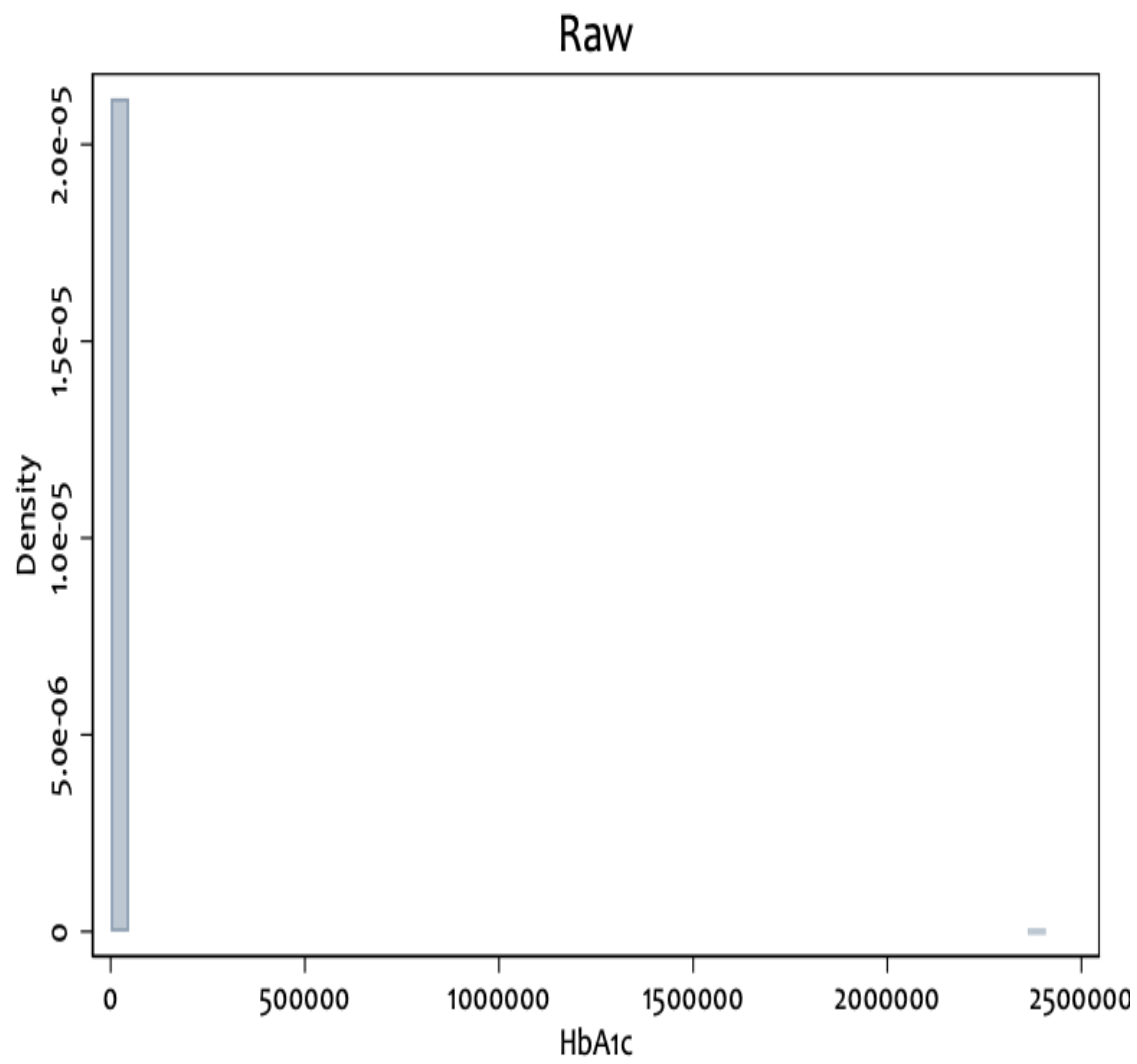

Total Protein

After cleaning, 204440 (24%) of patients have a measurement of totalprot in the 1 year prior to index

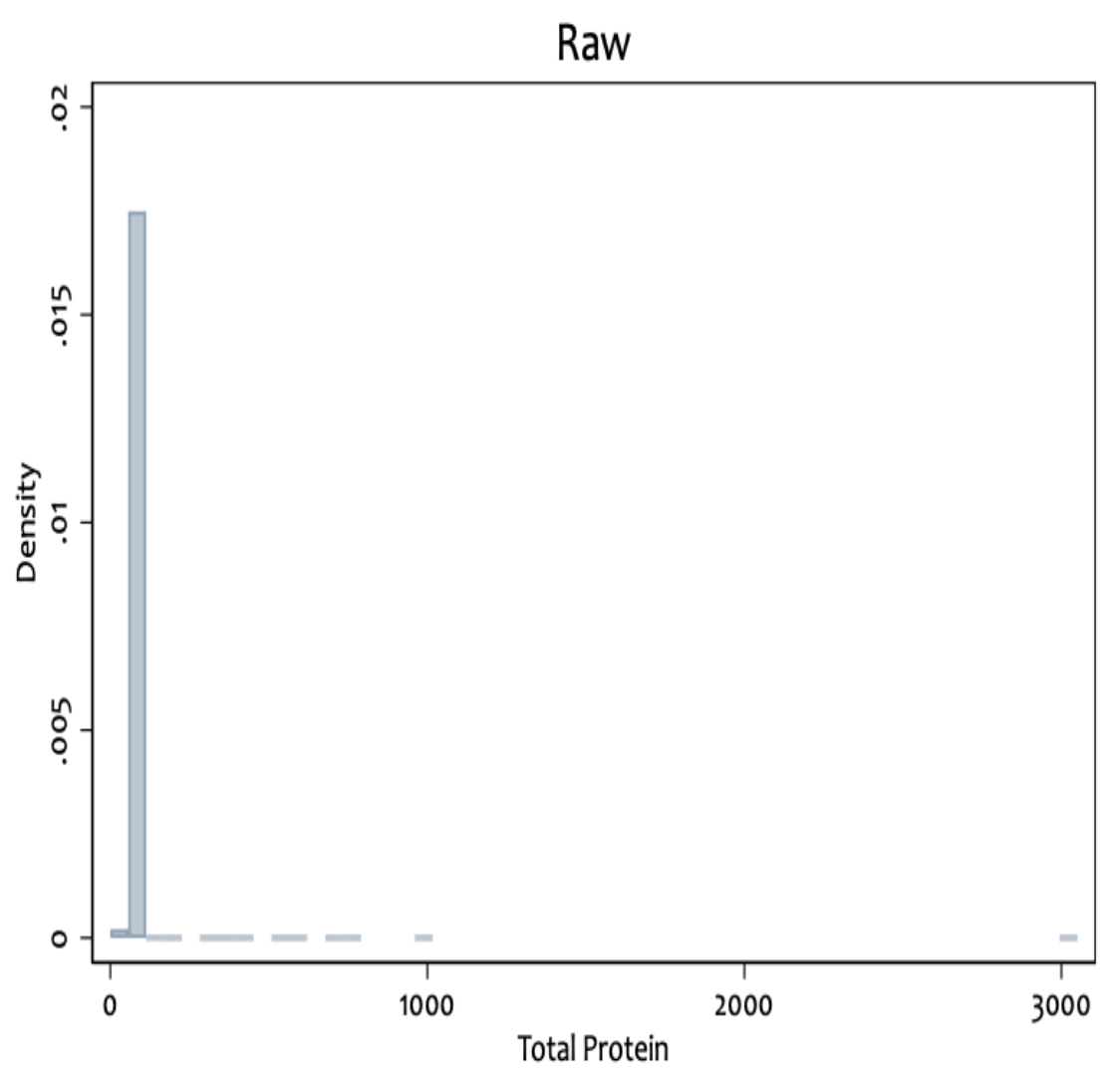

Urate

After cleaning, 11824 (1%) of patients have a measurement of urate in the 1 year prior to index

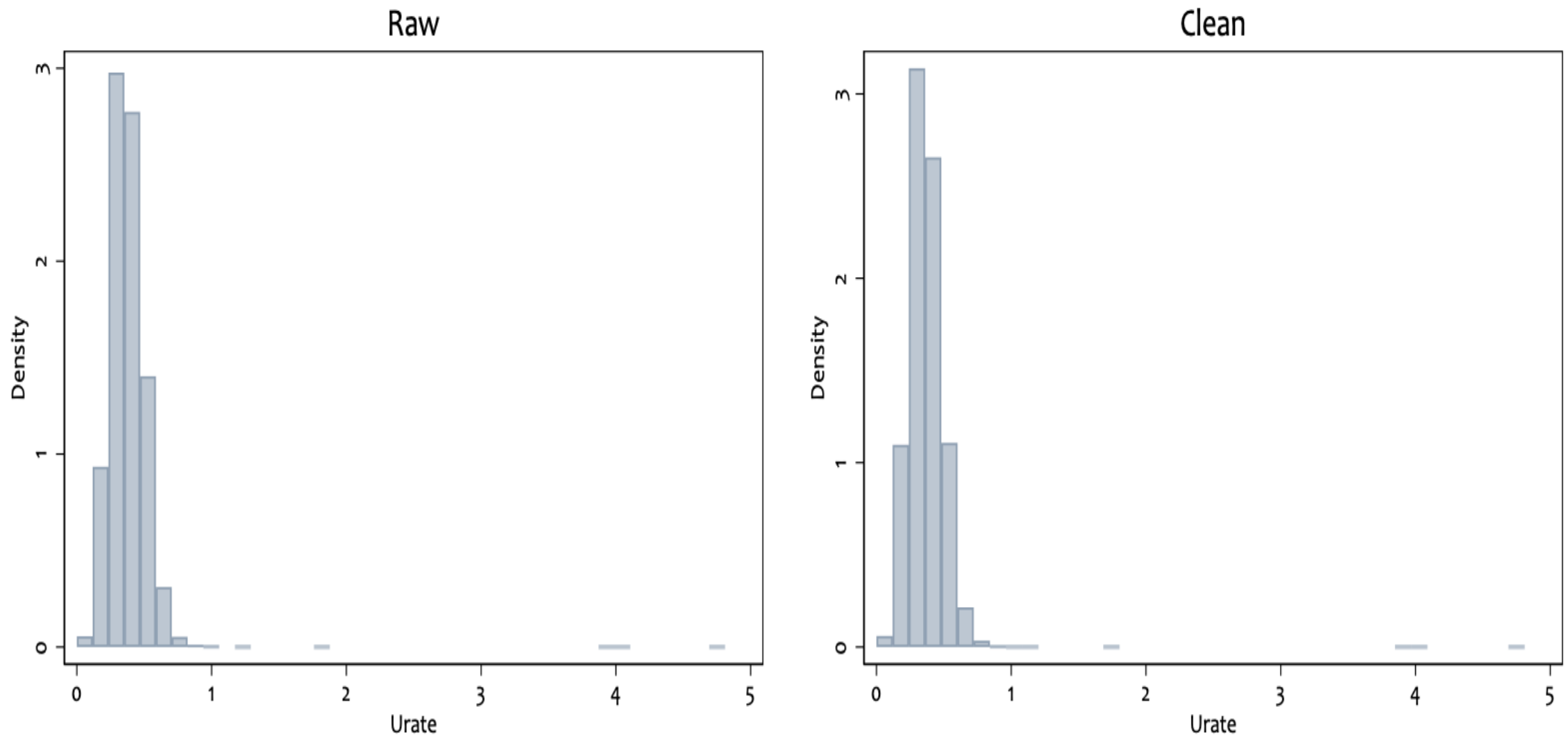
Raw
Density
Urate
0
1
2
3
4
5
Clean
Density
Urate

Test results in the year prior to index

SBP

After cleaning, 816076 (95.08%) of patients have a measurement of sbp in the 1 year prior to index

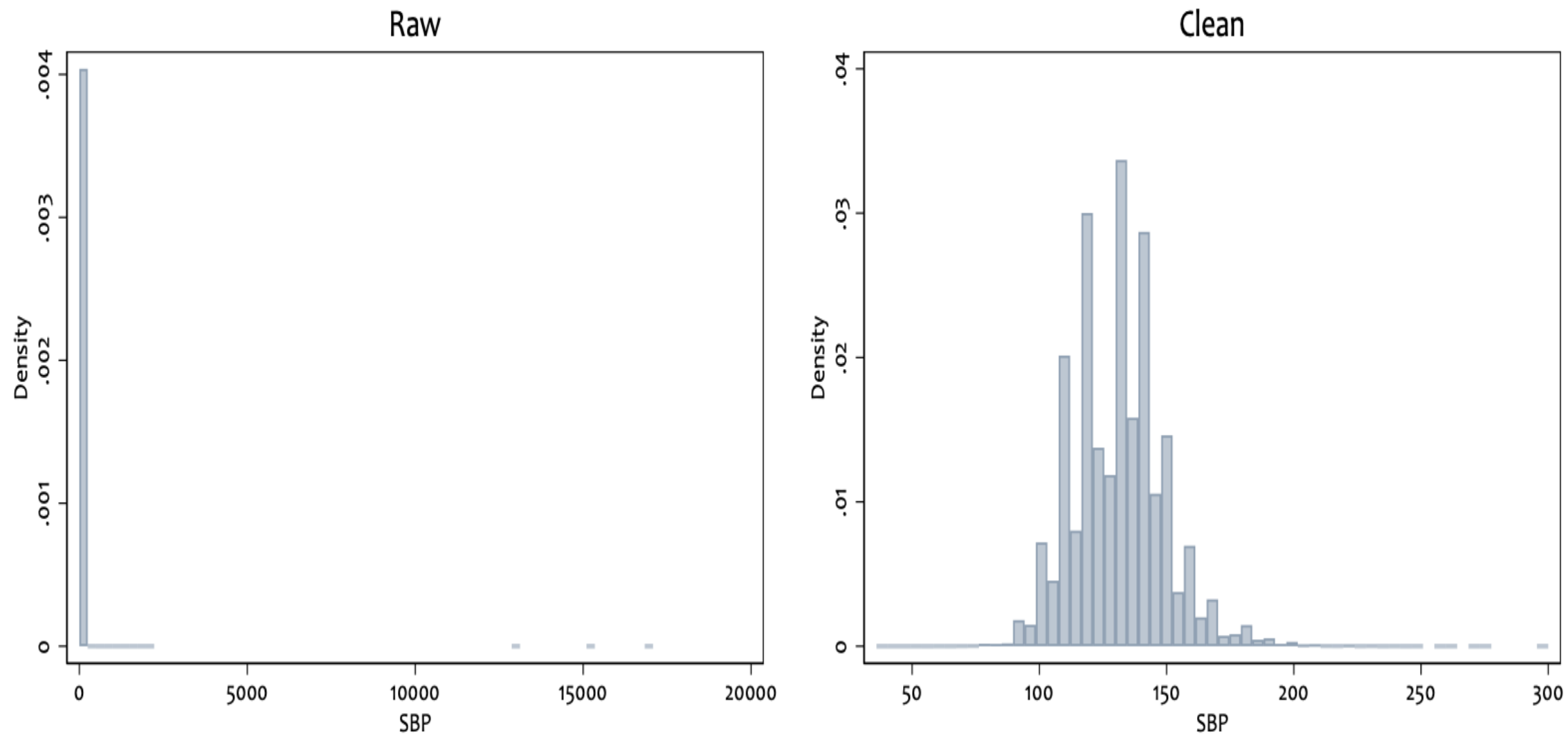

DBP

After cleaning, 816076 (95.08%) of patients have a measurement of dbp in the 1 year prior to index

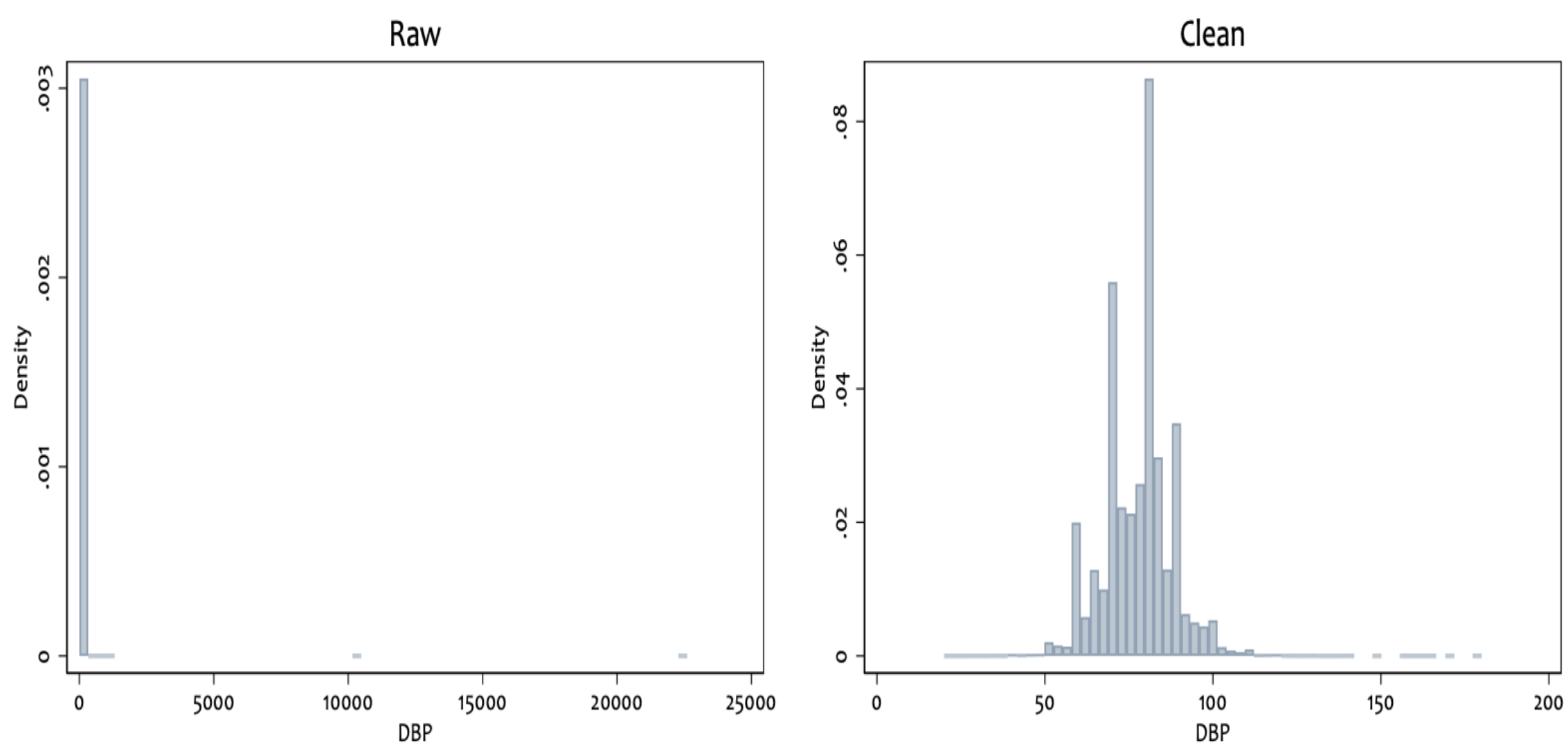

Appendix 3: Continuous blood test results incorporated

The 19 continuous variables incorporated for the final analysis are as follows:

Potassium, Sodium, Bilirubin, Alkaline Phosphatase (AKP), Creatinine, Alkaline Phosphatase Level (ALP), Alanine Aminotransferase (ALT), Lymphocyte, Urea, Mean Corpsucular Volume (MCV), Platelets, Haemoglobin, White Blood Count (WBC), Eosinophil, Mean Corpsucular Haemoglobin (MCH), Cholesterol, Total Protein, Diastolic Blood Pressure, and Albumin

Appendix 4: Propensity score distributions

Figure A1: Estimated propensity score distribution for the investigator-specified analysis

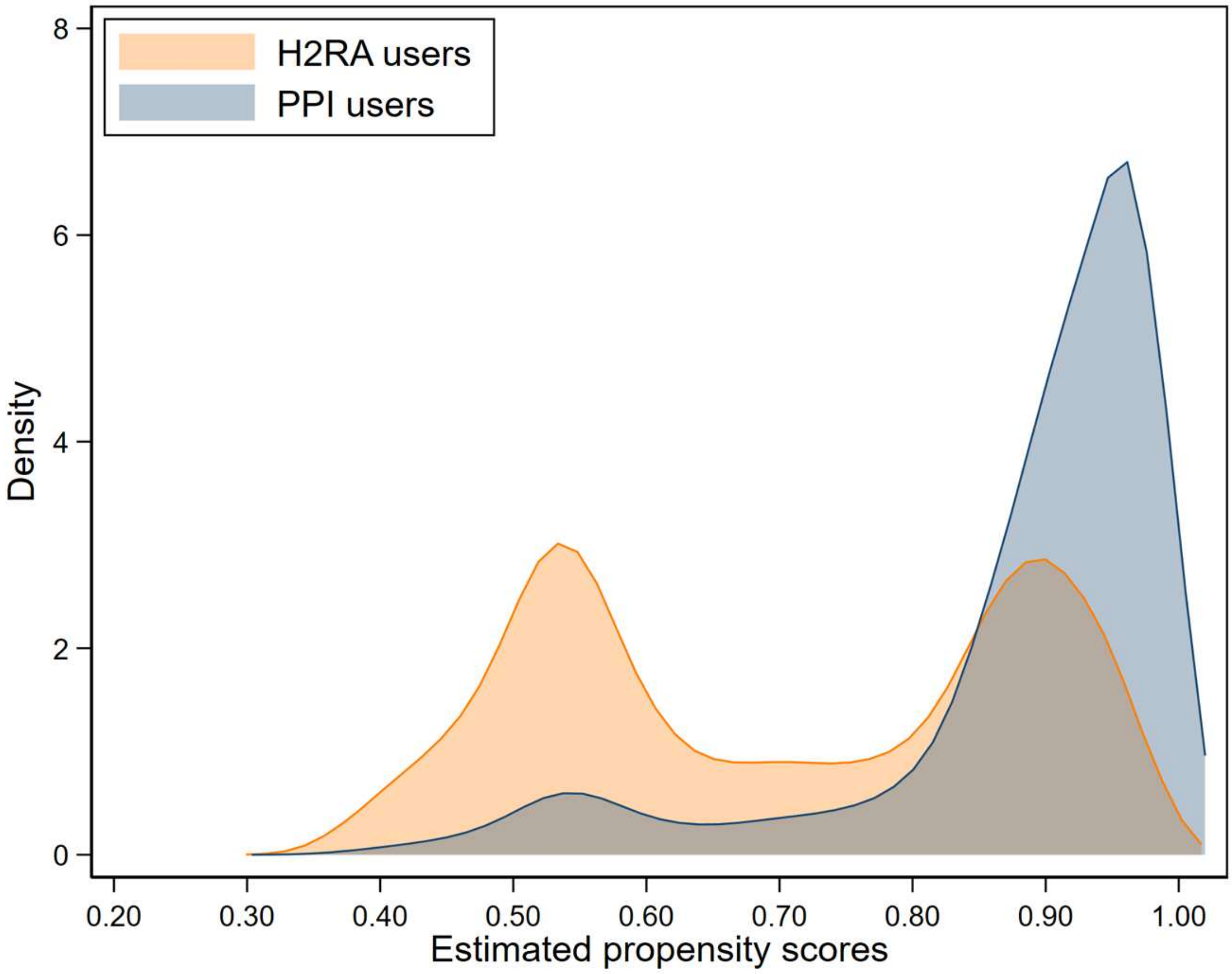

Figure A2: Estimated propensity score distributions for the high-dimensional propensity score analyses containing clinical, referral and therapy dimensions.

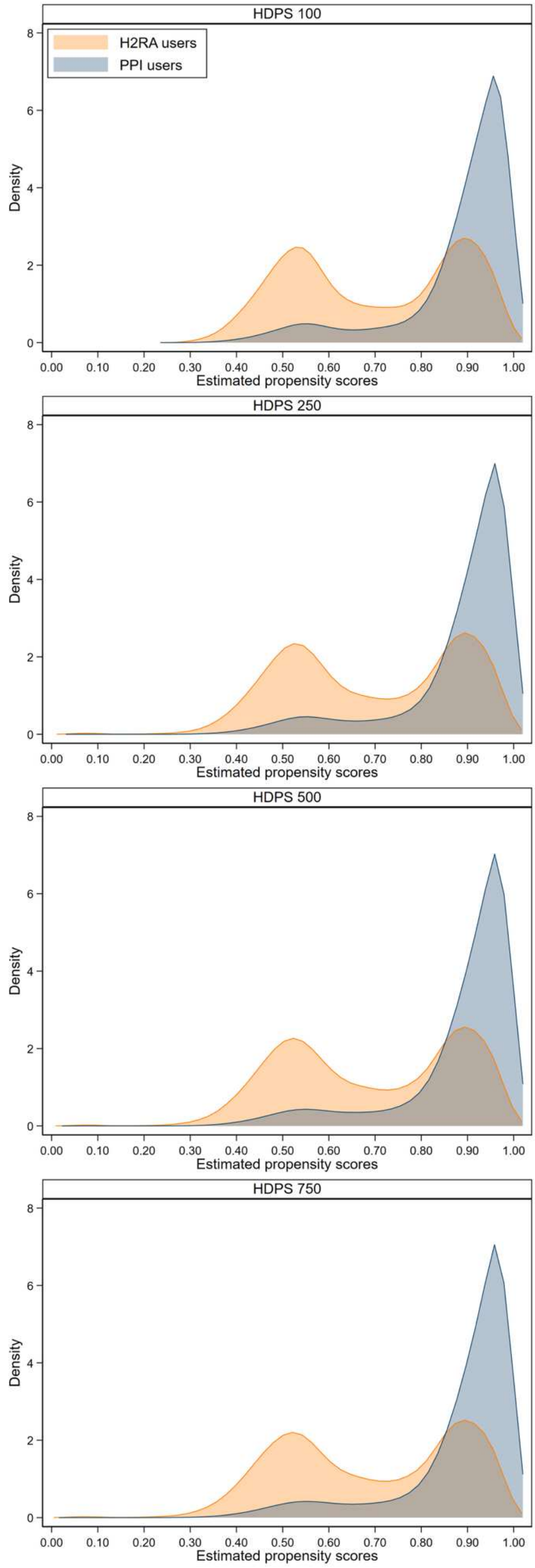

Figure A3: Estimated propensity score distributions for the high-dimensional propensity score analyses containing clinical, referral, therapy and test-requested dimensions.

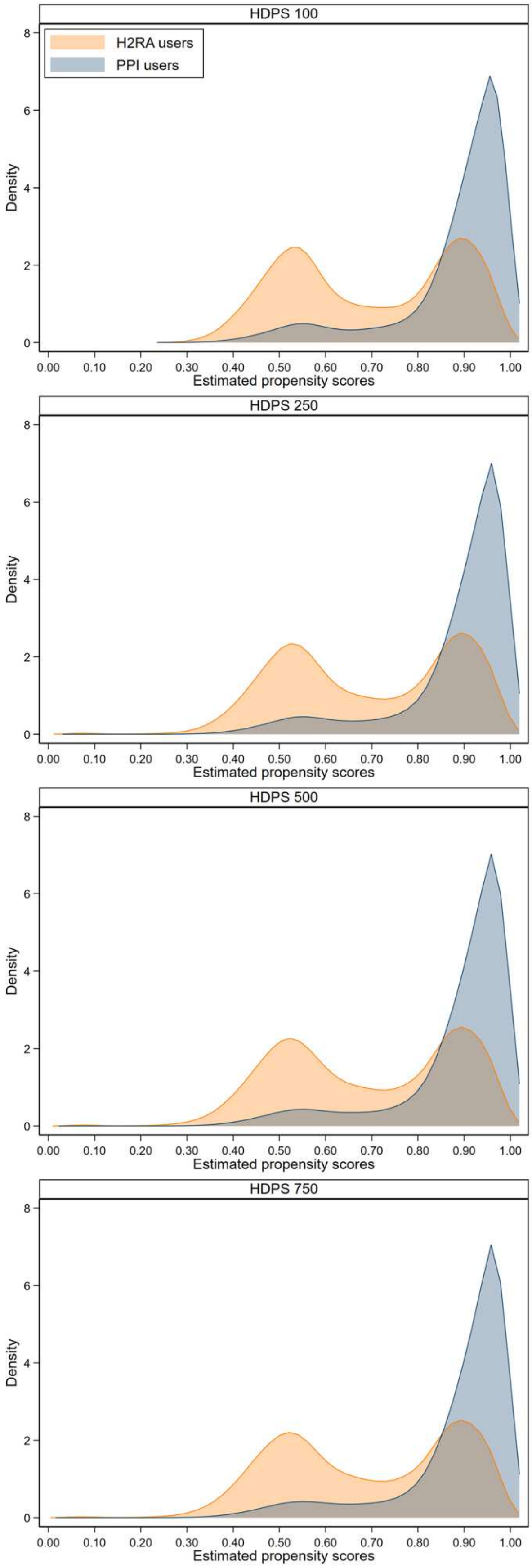

Figure A4: Estimated propensity score distributions for the high-dimensional propensity score analyses containing clinical, referral, therapy, test-requested and test cut-off dimensions.

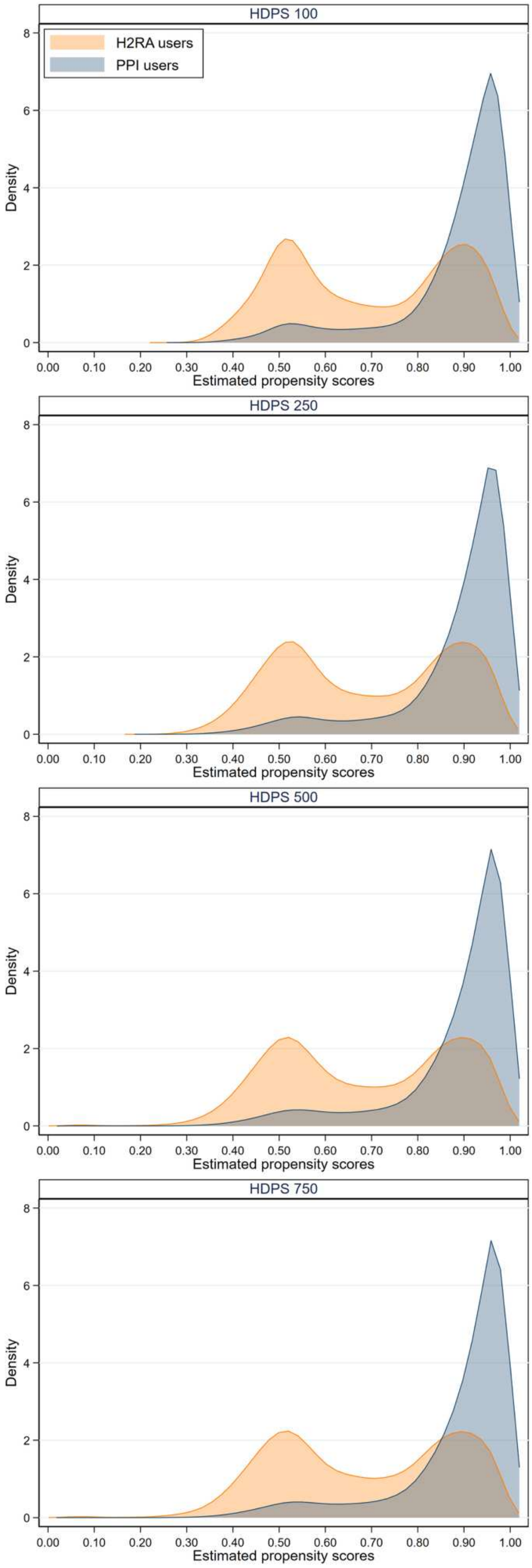

Figure A5: Estimated propensity score distributions for the high-dimensional propensity score analyses containing clinical, referral, therapy, test-requested and test cut-off dimensions. Corresponding continuous test variables included for any test cut-off variable appearing in top 100 covariates.

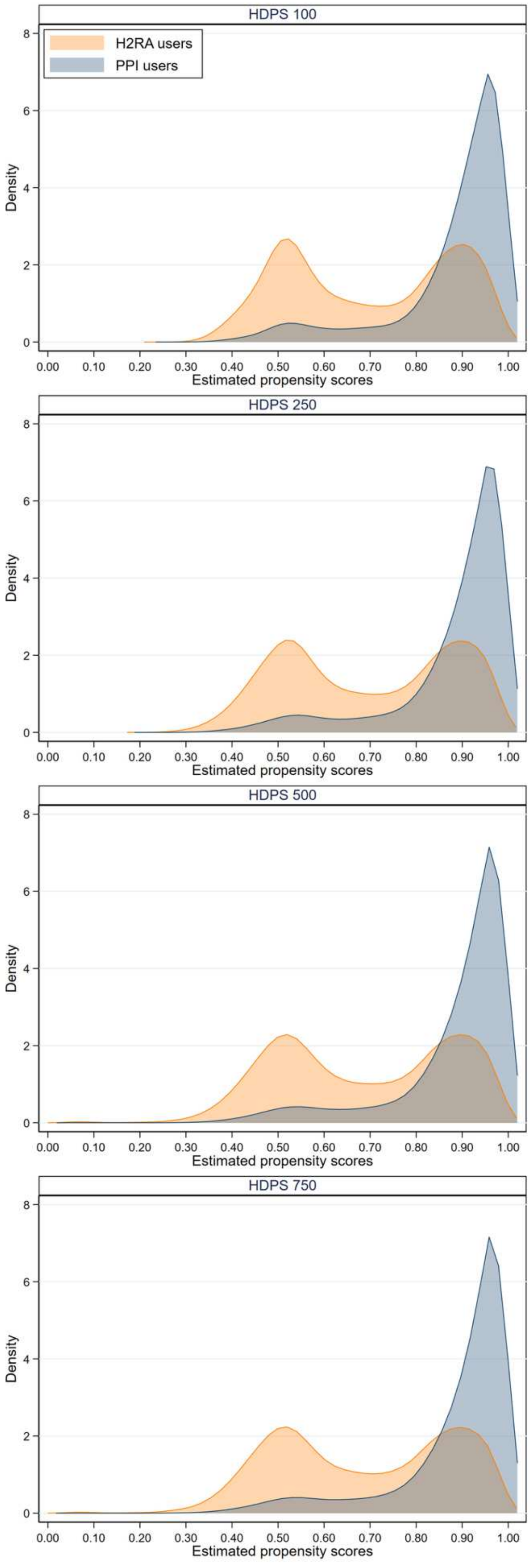

Appendix 5: Standardised mean differences

Figure A6: Comparison of absolute standardised differences (ASDs) between unweighted and weighted samples in the investigator-specified analysis. Dashed lines indicate ASDs of 10%.

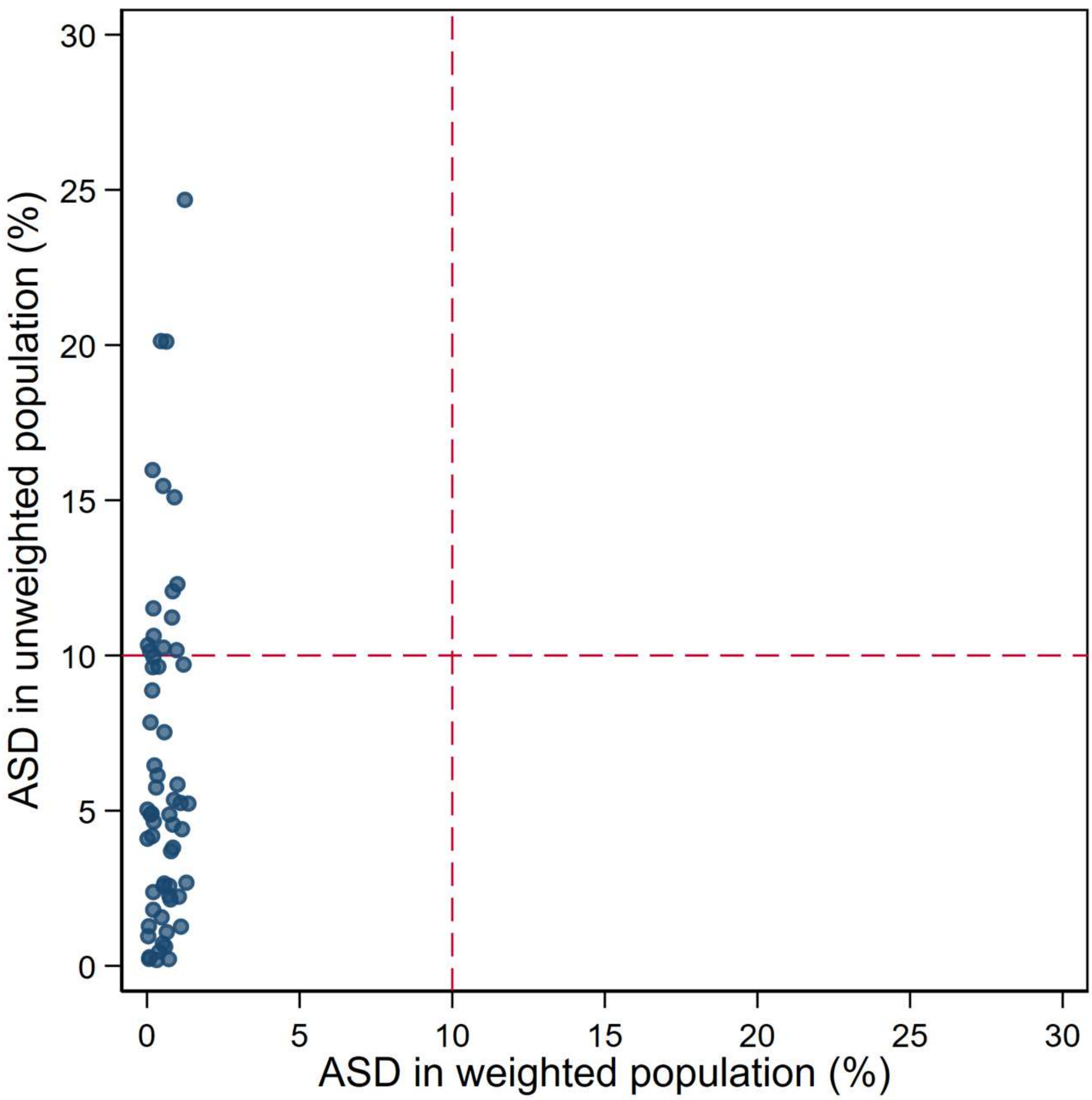

Figure A7: Comparison of absolute standardised differences (ASDs) between unweighted and weighted samples in the high-dimensional propensity score analyses considering clinical, referral and therapy dimensions. Dashed lines indicate ASDs of 10%.

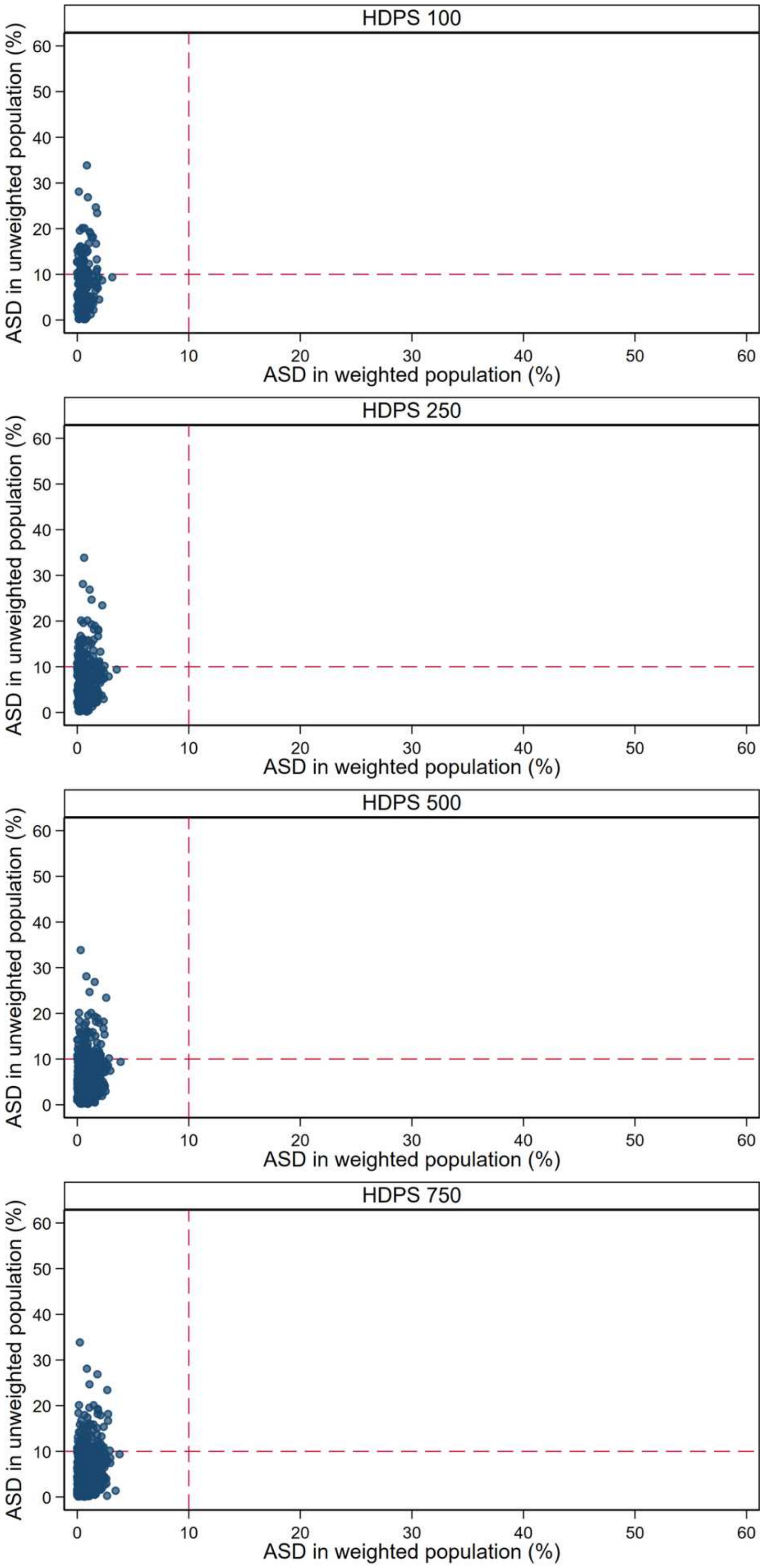

Figure A8: Comparison of absolute standardised differences (ASDs) between unweighted and weighted samples in the high-dimensional propensity score analyses considering clinical, referral, therapy and test-requested dimensions. Dashed lines indicate ASDs of 10%.

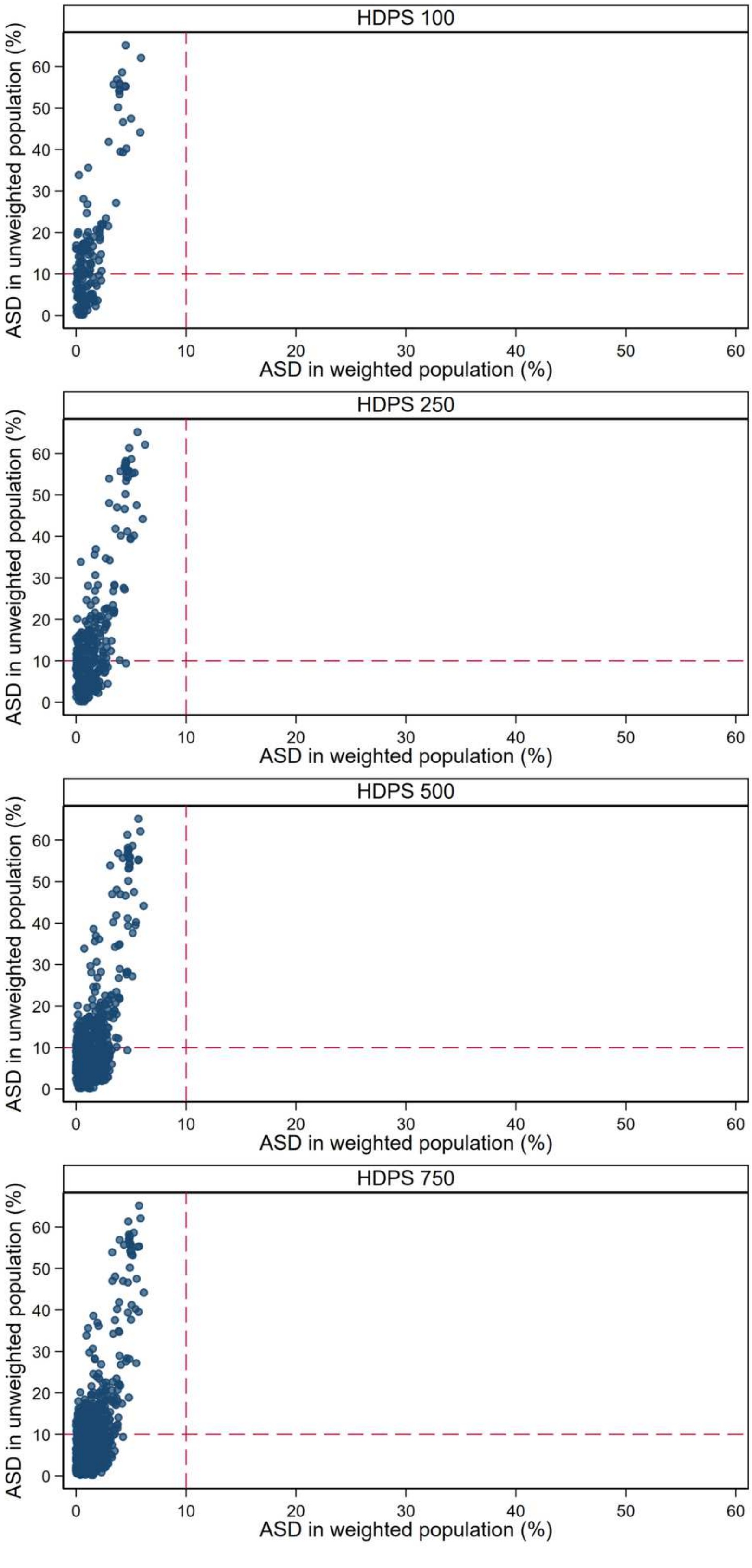

Figure A9: Comparison of absolute standardised differences (ASDs) between unweighted and weighted samples in the high-dimensional propensity score analyses considering clinical, referral, therapy, test-requested and test cut-off dimensions. Dashed lines indicate ASDs of 10%.

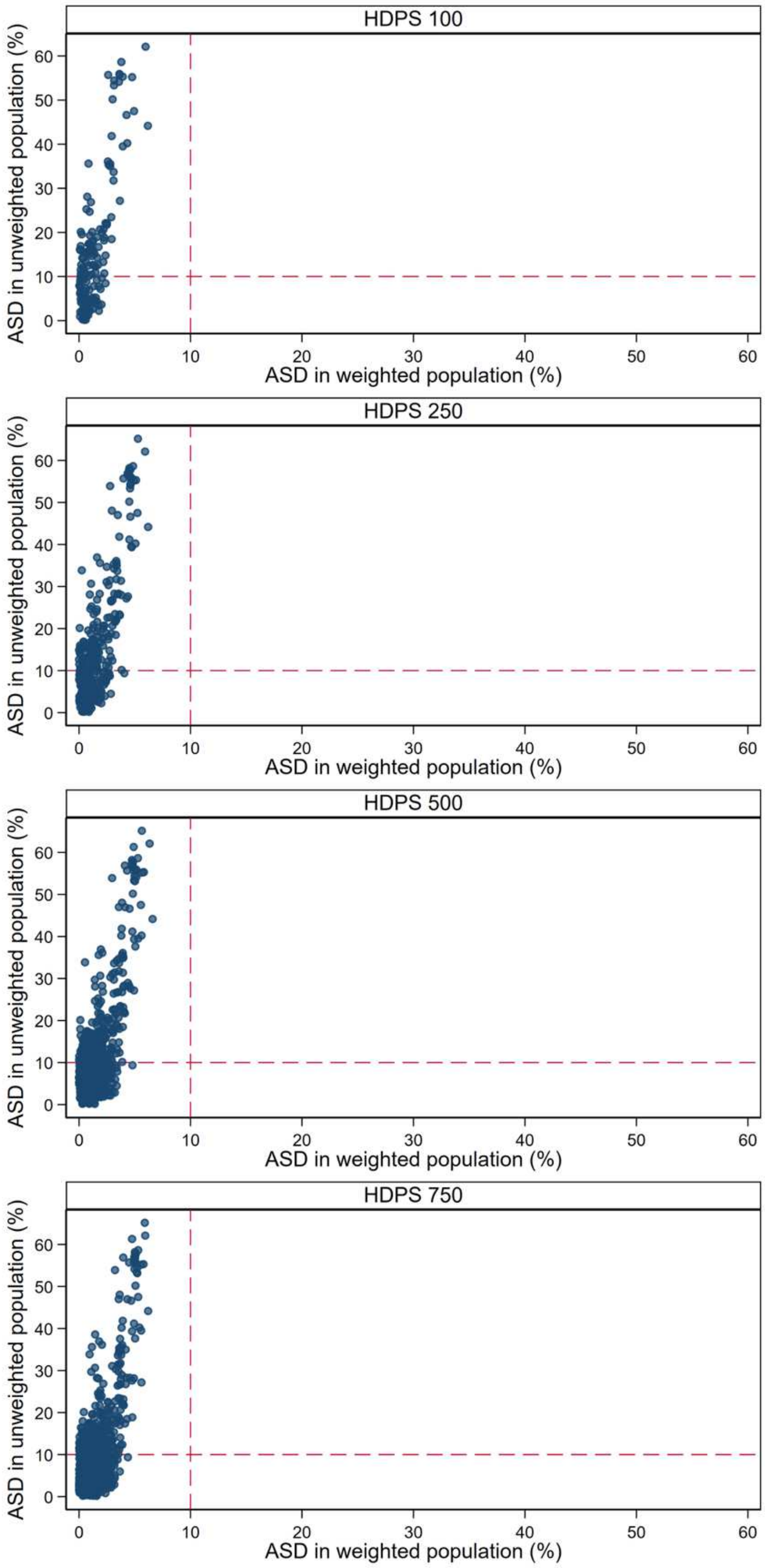

Figure A10: Comparison of absolute standardised differences (ASDs) between unweighted and weighted samples in the high-dimensional propensity score analyses considering clinical, referral, therapy, test-requested and test cut-off dimensions. Corresponding continuous test variables included for any test cut-off variable appearing in top 100 covariates. Dashed lines indicate ASDs of 10%.

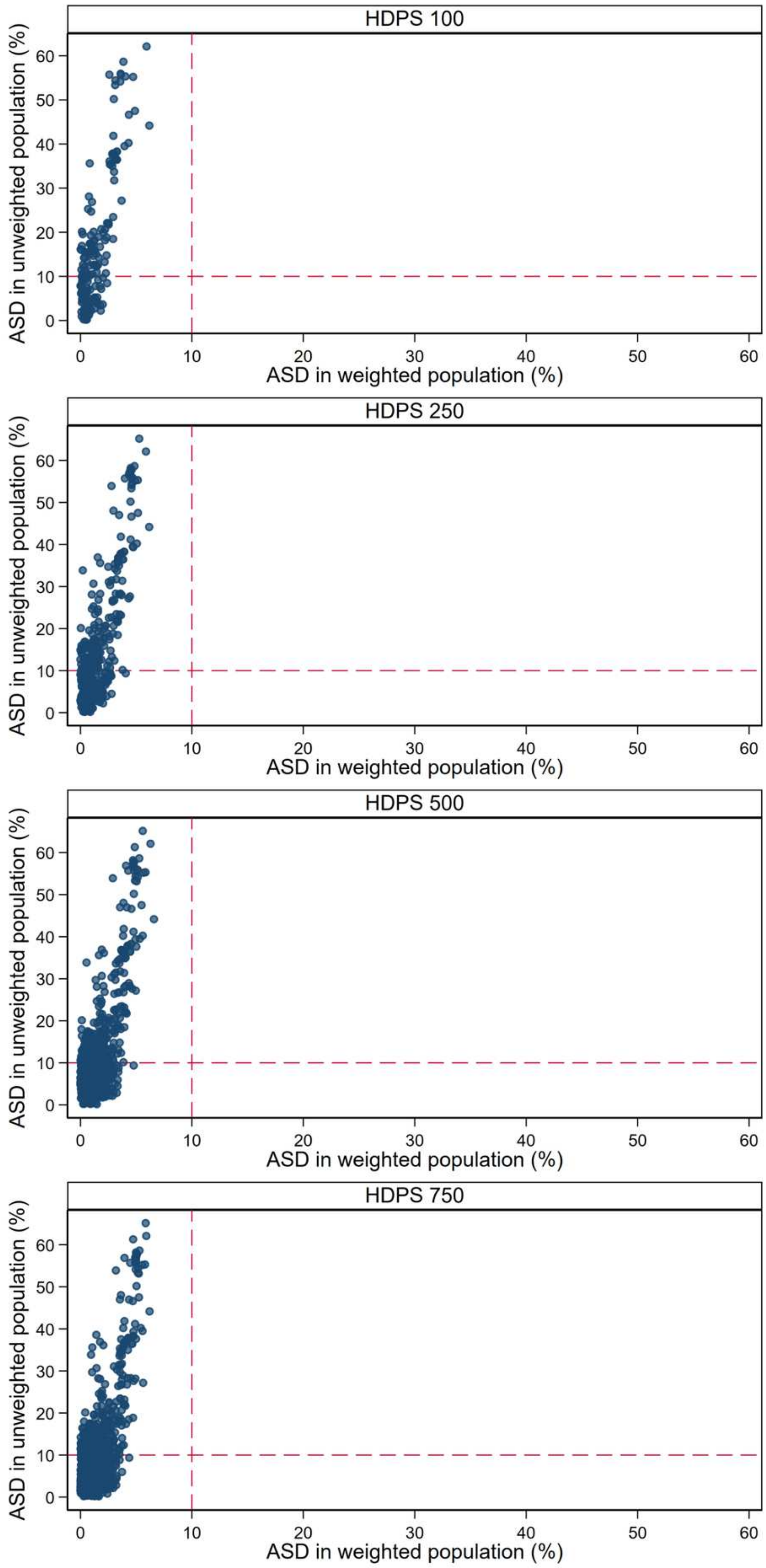

Supplement: Supplementary file 1 [file 12874_2026_2926_MOESM1_ESM.pdf]
